# Simple activation by acid of latent Ru-NHC-based metathesis initiators bearing 8-quinolinolate co-ligands

**DOI:** 10.3762/bjoc.12.17

**Published:** 2016-01-28

**Authors:** Julia Wappel, Roland C Fischer, Luigi Cavallo, Christian Slugovc, Albert Poater

**Affiliations:** 1 Institute for Chemistry and Technology of Materials, Graz University of Technology, NAWI Graz, Stremayrgasse 9, 8010 Graz, Austria; 2 Institute of Inorganic Chemistry, Graz University of Technology, Stremayrgasse 9, 8010 Graz, Austria; 3 KAUST Catalysis Center, Physical Sciences and Engineering Division, King Abdullah University of Science and Technology, Thuwal 23955-6900, Saudi Arabia; 4 Institut de Química Computacional i Catàlisi, Departament de Química, Universitat de Girona, Campus de Montilivi, E-17071 Girona, Spain

**Keywords:** acid, activation by acid, metathesis, polymer, quinolin, ruthenium, triggerable

## Abstract

A straightforward synthesis utilizing the ring-opening metathesis polymerization (ROMP) reaction is described for acid-triggered N,O-chelating ruthenium-based pre-catalysts bearing one or two 8-quinolinolate ligands. The innovative pre-catalysts were tested regarding their behavior in ROMP and especially for their use in the synthesis of poly(dicyclopentadiene) (pDCPD). Bearing either the common phosphine leaving ligand in the first and second Grubbs olefin metathesis catalysts, or the Ru–O bond cleavage for the next Hoveyda-type catalysts, this work is a step forward towards the control of polymer functionalization and living or switchable polymerizations.

## Introduction

The modulation of the activity of enzymes by chemical triggers, e.g., by allosteric binding is ubiquitous in nature [1–2], but exploiting similar strategies for synthetic catalysts is still in its infancy [3–5]. Prominent examples in catalytic polymerization [6–7] comprise the regulation of molecular weight by allosteric effects [8–9] or influencing the polymers’ microstructure upon changing the monomer pressure [10]. Such kind of regulation of the catalysts activity does not only comprise a switching on of a particular feature but also the switching off of this feature upon another (different) stimulus. A simpler but related concept is to turn a latent catalyst [11] or initiator (i.e., ideally a completely inactive pre-catalyst/initiator) into an active form by an external trigger [12]. This principle is well and long known and frequently exploited in polymer chemistry where thermally or photochemically switchable initiators are the key for many applications of, e.g., radically or cationically prepared polymers [13–15].

Focusing on the origin of organic synthesis, basically based on reactions that drive to the formation of carbon–carbon bonds [16], olefin metathesis turns out to be one potential route to get unsaturated molecules bearing C–C double bonds [17–21], thus by extension polymers, as well. Olefin metathesis polymerizations are transition metal-mediated processes which emerged as powerful alternatives to these conventional polymerization methods [22–23]. Thus, it is not surprising that a series of latent but triggerable initiators have been disclosed in the last years [24–26]. The latent initiators are ideal if they have the capacity of storage in combination with the monomer for a long period [27]. Then the reaction only initiates once an appropriate exogenous stimulus is exerted [28–29]. Amongst this important property, the latent initiator should be as insensitive as possible to any other potentially present chemical, most importantly oxygen and water [30]. Particularly for the latter reason ruthenium based latent initiators play the most important role in literature [24,31–33].

Last but not least, bearing in mind changes in activity or levels of transforming growth factor-beta (TGF-beta) are associated with a broad variety of diseases and that TGF-beta is biologically inert when takes part of the complex that bears its corresponding peptide [34]. From this latter latent complex, most available immunoassays require controlled activation by acid to release the TGF-beta. On the other hand, myostatin belongs to the transforming growth factor 13 superfamily, known because it decreases the skeletal muscle mass. Bearing the fact that experiments have shown that myostatin activity is detected only after activation by acid [35], myostatin demonstrates to be a latent complex, and can be transported more easily. Once described the latter successful practical application of the acid triggered activation of an enzyme, here, at a chemical molecular level, we unravel the performance of the acid triggered N,O-chelating ruthenium based pre-catalysts [36] bearing one or two 8-quinolinolate ligands.

## Results and Discussion

### Synthesis and characterization

Herein we investigate 8-hydroxyquinoline derivatives as the chelating, “pacifying” ligands. 8-Hydroxyquinoline and its derivatives are known to be excellent ligands for many transition metals [37]. They can be readily electronically modified and many derivatives are commercially available. Furthermore, 8-hydroxyquinoline ligands are generally very cheap. Surprisingly this class of ligands is not frequently used in transition metal chemistry [38–40].

**Scheme 1:**
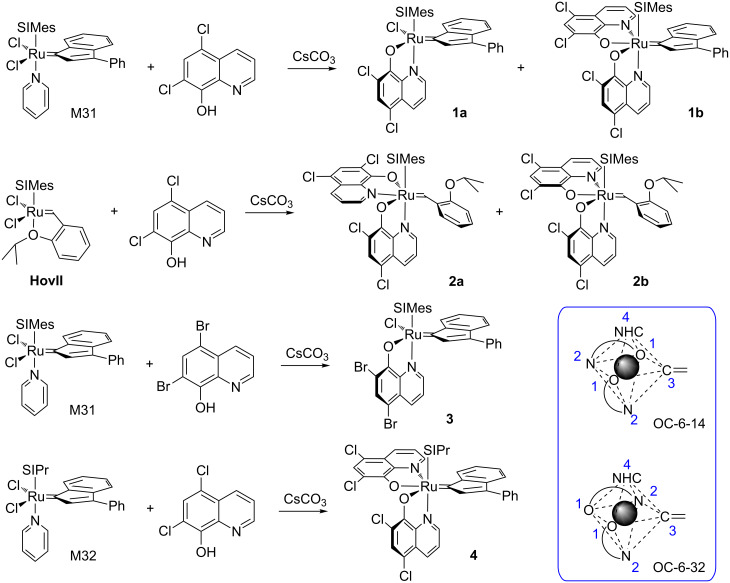
Synthesis of **1**-**4**; only the isolated and characterized complexes are shown.

The synthesis involved the reaction of N-heterocyclic carbene bearing precursor complexes **M31**, **HovII** or **M32** with excess of 5,7-dichloro-8-hydroxyquinoline or 5,7-dibromo-8-hydroxyquinoline in the presence of excess Cs_2_CO_3_ as the base (see Scheme 1). The silver-free method [41] resulted in any case in the formation of at least two new products (as evidenced by thin-layer chromatography) which were separated by means of column chromatography. All obtained complexes are very stable in the solid state and can be stored for several days in solution in the presence of oxygen without any sign of decomposition. Most striking, all complexes possess an outstanding solubility in nonpolar solvents such as *n*-pentane as well as in nonpolar substrates such as dicylopentadiene (DCPD). Both properties are desired properties for employing the compounds as initiators in solvent free polymerizations.

**Figure 1:**
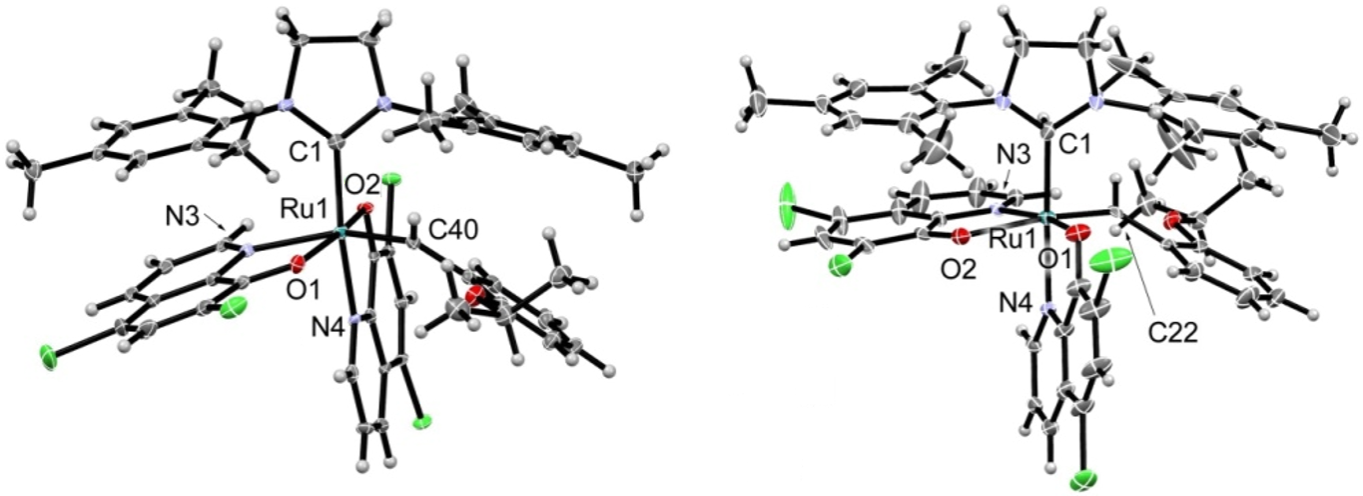
Solid state structure of complexes **2a** and **2b** as retrieved from single crystal X-ray diffraction.

In case of the reaction of **M31** with 5,7-dichloro-8-hydroxyquinoline two products could be isolated and characterized. The major product **1a** resulted from the exchange of one chloride ligand from **M31** for the oxygen of the quinolinolate and exchange of pyridine for the nitrogen of the incoming ligand. Thus the chelating quinolinolate is assumed to bind as it is expected from a series of published N,O-chelating ligands [42]. The minor isolated product **1b** featured two 5,7-dichloro-8-quinolinolate ligands and was identified as the OC-6-32 isomer (see Scheme 1). Complexes **1a** and **1b** account for 90% of the theoretical yield. In case of **HovII** as the starting complex again two products, **2a** and **2b**, were isolated. In this case both complexes featured, according to NMR analysis, two 5,7-dichloro-8-quinolinolate ligands. The overall yield amounted to 83% in this case. Single crystal X-ray structure analyses elucidated the solid state structure of **2a** and **2b** (see Figure 1). The minor isomer **2a** (28% yield) was identified as the OC-6-14 diastereomer featuring the two oxygen atoms in *trans* disposition (and the nitrogen atom *trans* to the benzylidene ligand). The major isomer **2b** (57% yield) is the OC-6-32 diastereomer bearing the two oxygen ligands in *cis* disposition (and one oxygen atom is coordinated *trans* to the benzylidene ligand).

Elemental analysis confirmed the proposed stoichiometry of the complexes. Characteristic ^1^H NMR signals comprise the protons in position 2 of the quinolinolate moieties. These protons resonate in the low-field (between 9.00 ppm in **2b** and 7.90 ppm in **3**) when the N-heterocyclic carbene ligand is situated *trans* to the N-atom of the quinolinolate. The corresponding proton of the second quinolinolate ligand (with the N-atom situated *cis* to the NHC) is high-field shifted and resonates at 5.48 (in **1b**), 5.32 (in **2b**) and 5.9 (in **4**). The OC-6-14 derivative **2a** is characterized by a pronounced low-field shift of the latter signal to about 6.1 ppm (signal superimposed by other resonances). Based on these observations an OC-6-32 stereochemistry is tentatively assigned to complex **1b**. Decoordination of the isopropoxy group during formation of **2a** and **2b** is readily evident from the benzylidene proton chemical shifts of 19.10 and 18.24 ppm, which are distinctly low-field shifted in comparison to the same signal in **HovII** (16.56 ppm). From single crystal X-ray structure analyses of **2a** and **2b** it became evident that, in general, bond lengths and angles are very similar to each other. For example, the Ru–NHC bond is in both complexes 2.05 ± 0.01 Å and the Ru–benzylidene bonds measure 1.89 ± 0.01 Å. Of interest are the different bond lengths of the two quinolinolate ligands. While the first quinolinolate in the OC-6-14 derivative **2a** (N *trans* to the NHC and O trans to O of the second quinolinolate moiety) exhibits a Ru–N bond length of 2.11 Å and a Ru–O bond lengths of 2.04 Å, the second quinolinolate ligand (with N *trans* to the benzylidene) show distinctly longer Ru–O (2.09 Å) and Ru–N (2.20 Å) bonds. In the OC-6-32 derivative **2b**, bond lengths of the first quinolinolate are similar as in **2a** (Ru–N 2.12 Å and Ru–O 2.04 Å). The coordination of the second quinolinolate in characterized by a Ru–N bond of 2.08 Å and a Ru–O bond length of 2.17 Å. In contrast to related complexes described by Grubbs et al. no isomerization of **2a** into **2b** or the other way round was observed upon heating at 80 °C for 48 h [43].

### Catalytic activity

The polymerization activity of the complexes was initially tested using dimethyl bicyclo[2.2.1]hept-5-ene-2,3-dicarboxylate (**5**) as the benchmark monomer. Monomer **5** was used because its polymers are not prone to backbiting. Therefore the average number molecular weight (*M*_n_) can be used to characterize the ratio of initiation rate to propagation rate (*k*_i_/*k*_p_) of a given initiator to monomer combination [28,44–46]. Under the applied conditions (prolonged reaction time, reaction temperature up to 100 °C, UV-irradiation) none of the initiators was able to convert the monomer to a polymer. Therefore, efforts to activate the initiators via acid have been made. Upon addition of HCl aq complexes **1** to **4** became active and initiated the ROMP reaction of **5**. The activation process is accompanied by a colour change from deep red, to brownish to dark green in complexes **1**–**3** and from brownish to red to yellow in **4**. The reaction progress of the polymerization reaction was monitored using thin-layer chromatography. After complete consumption of the monomer, the reaction was stopped with an excess of ethyl vinyl ether, precipitated in vigorously stirred methanol and dried. Noteworthy, hydrochloric acid is the only acid which activates the preinitiators under investigation. Addition of other acids (or acid liberating reagents) such as acetyl chloride or trifluoroacetic acid, were not able to promote the polymerization. Also the addition of chloride containing salts, e.g., triethanolamine hydrochloride failed in promoting the metathesis reaction. Therefore, initiators **1**–**4** were benchmarked in the polymerizations of **5** using 50 equiv HCl. The reactions were carried with a [initiator]:[**5**] ratio of 1:300, at room temperature in CH_2_Cl_2_.

Table 1 summarizes the results of the polymerization data, bearing substrate **5** (see Supporting Information File 1 for further details). Generally it can be concluded, that except of **4**, the initiators show a relatively slow initiation compared to their propagation. The high molecular weights indicate a slow activation process or incomplete activation. In contrast preinitiator **4** yields polymers with low *M*_n_ and PDI values indicating an almost full activation of the preinitiator by the acid. The PDI’s are slightly increased compared to the values of known living initiators such as **M31** [47].

**Table 1:** Polymerization of **5** by preinitiators **1**–**4** (eth = ethereal; aq = aqueous; molecular weights (*M*_n_) and the corresponding polydispersity indices (PDI) were determined using gel permeation chromatography (GPC) against polystyrene standards).

Complex	Temperature [°C]	Activation	Time [h]	Conversion [%]	Isol. yield [%]	*M*_n_ [kg/mol]	PDI

**1**–**4**	20	–	24	–	–	–	–
**1**–**4**	80	–	24	–	–	–	–
**1**–**4**	20	UV light	24	–	–	–	–
**1a**	20	HCl eth	6.25	100	75	413	2.0
**1b**	20	HCl eth	2	100	78	181	1.9
**2a**	20	HCl eth	23	65	42	254	2.2
**2b**	20	HCl eth	4	100	85	148	2.4
**3**	20	HCl eth	24	76	23	278	2.1
**4**	20	HCl eth	2.15	100	45	48	1.3
**1a**	20	HCl aq	3	95	80	392	2.0
**1b**	20	HCl aq	1.25	100	84	196	1.7
**2a**	20	HCl aq	23	44	20	296	1.8
**2b**	20	HCl aq	4.5	100	78	266	1.8
**3**	20	HCl aq	23	66	20	275	1.8
**4**	20	HCl aq	2	100	58	52	1.4
**1a**	80	HCl aq	2.25	100	84	411	2.1
**1b**	80	HCl aq	1	100	88	159	1.9
**2a**	80	HCl aq	24	77	46	132	2.3
**2b**	80	HCl aq	1	100	83	418	2.2
**3**	80	HCl aq	1.25	100	81	142	1.7
**4**	80	HCl aq	0.75	100	75	52	1.6

A striking observation is that **2a** and **3**, even if according to the demonstrated activation mechanism should form the same active species as **1a**, **1b** and **2b** they show different polymerization behaviour. Bearing the fact that **3** has only one quinolinate ligand when compared to **1a**, and differentiated by the size of the substituents in the α-position to the oxygen group, hypothetically, more energy for the dissociation of the quinolinolate ligands is required maybe caused by the different geometric arrangement of the ligands around the ruthenium centre. This speculation is supported by the reactions performed at 80 °C where **3** (which possess just one quinolinolate ligand) reaches full conversion after the approximately same time as the other initiators.

**Figure 2:**
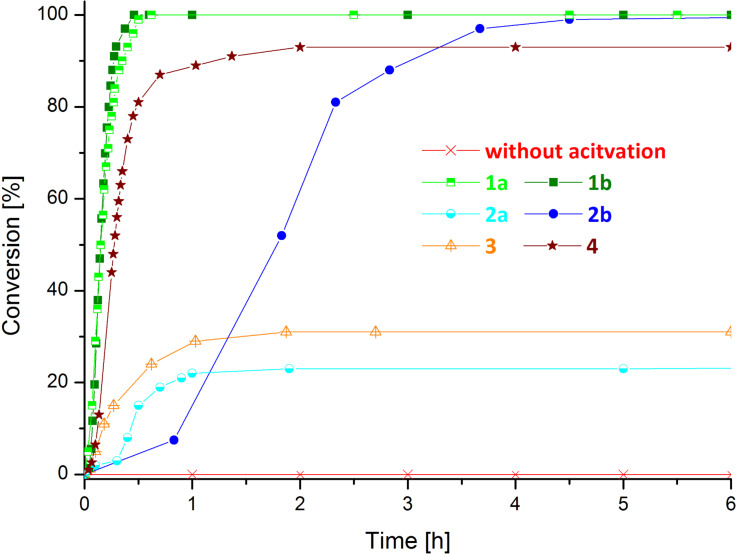
Time/conversion plot for the polymerization of **5** by preinitiators **1**–**4** in the presence of HCl ([**5**]:[HCl]:[I] = 50:25:1; [**5**] = 0.1 mol/L; solvent:CDCl_3_).

Additionally time/conversion plots were acquired via arrayed ^1^H NMR measurements of the polymerization of **5** initiated by **1**–**4** and HCl (see Figure 2 and Supporting Information File 1 for further details) in the presence of air. The fastest polymerizations show the indenylidene derivatives bearing dichloroquinolinolate ligand(s) (**1a**, **1b** and **4**). Preinitiator **2b** is slower in converting **5** but still satisfying conversion is obtained after about 4.5 hours. In contrast, preinitiators **2a** and **3** provided distinctly worse conversions. It is noteworthy that **2a** as well as **3** perform better in the Schlenk experiments (which were performed under nitrogen atmosphere) presented above.

**Figure 3:**
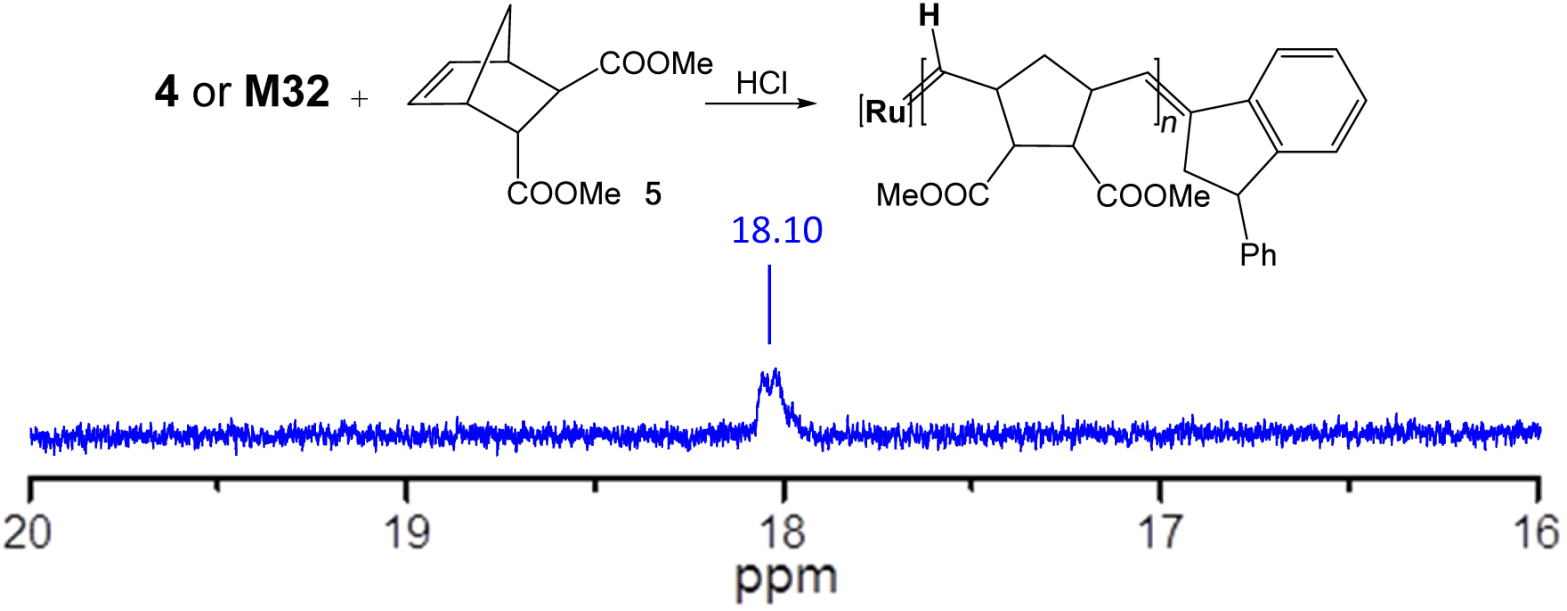
^1^H NMR spectrum in the low-field region of the active species for complexes **4** and **M32**.

To elucidate the activation mechanism of complexes **1**–**4** upon HCl addition, the actual active species of the initiators was investigated. For that purpose, **4** was mixed with 5 equiv of monomer **5** in CDCl_3_ and activated it with 5 equiv of etherical HCl in a NMR tube. After few minutes, the characteristic carbene peak for propagating alkylidenes at 18.1 ppm appeared (see Figure 3). To identify this carbene peak, the same experiment was repeated reacting **M32** with **5**, leading to the same characteristic carbene peak. We assume that, as proposed by Grubbs and co-workers [43], the hydrochloric acid protonates both ligands which are subsequently exchanged by chlorides forming the same active 14-electron species as the original starting complex **M32** does. Trapping the active species of the SIMes analogues failed.

**Scheme 2:**
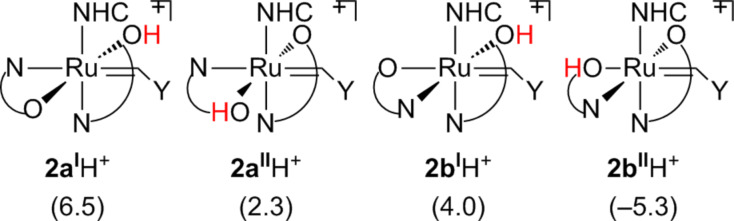
Energetics of **2a** and **2b** protonation in kcal/mol.

To shed light about the different behavior of complexes **2a** and **2b** we envisaged DFT calculations. The optimized geometry of **2b** is in perfect agreement with the X-ray structure [48] (rmsd = 0.032 Å and 0.9° for the selected main distances and angles) [49–50]. In agreement with experiments that indicated **2b** as the most stable isomer, calculations estimate that **2b** is 2.0 kcal/mol more stable than **2a**. To rationalize the different reactivity of **2a** and **2b** we compared the basicity of the four O atoms by calculating the energy of the acid–base equilibrium (see Scheme 2), where [Ru] is **2a** or **2b**, [Ru]H^+^ is **2a** or **2b** with a protonated O atom. The energy of [Ru] and [Ru]H^+^ is calculated in CH_2_Cl_2_ using the protocol described in the computational details sections, while for the aqueous solvation free energy of the proton we assumed the value of −262.2 kcal/mol from the literature [51–52]. It is assumed that the protonated Ru species remains in the organic phase. The energetics for the four oxygen protonated species of **2a** and **2b** is reported in Scheme 2.

According to the number reported, protonation of one O atom of **2a**, leading to species **2a^I^H^+^** and **2a^II^H^+^**, is unfavored, as well as protonation of the O atom of **2b** *cis* to the NHC ligand, leading to **2b^I^H^+^**. The only O atom presenting favorable protonation energy is the *trans* one to the Ru–alkylidene bond of **2b**, leading to **2b^II^H^+^**. The accuracy of the absolute protonation energies are difficult to estimate, since they can vary with the computational protocol (i.e*.*, functional, basis set and solvation model) and they also depend on experimental considerations, bearing the assumption that all the Ru species are in the organic phase, while HCl is dissociated in the aqueous phase. For this reason the absolute value of the protonation energies is not stressed further. However, the relative trend in the protonation energies is in agreement with the general idea that protonation of the O atom *trans* to the Ru–alkylidene bond should be favored, since this leads to a much softer –OH ligand *trans* to the Ru–alkylidene bond. Consistently, the protonated Ru–O distance increases by roughly 0.11 Å in **2a^I^H^+^**, **2^II^H^+^**, and **2b^I^H^+^**, whereas it increases by 0.18 Å in **2b^II^H^+^**.

**Figure 4:**
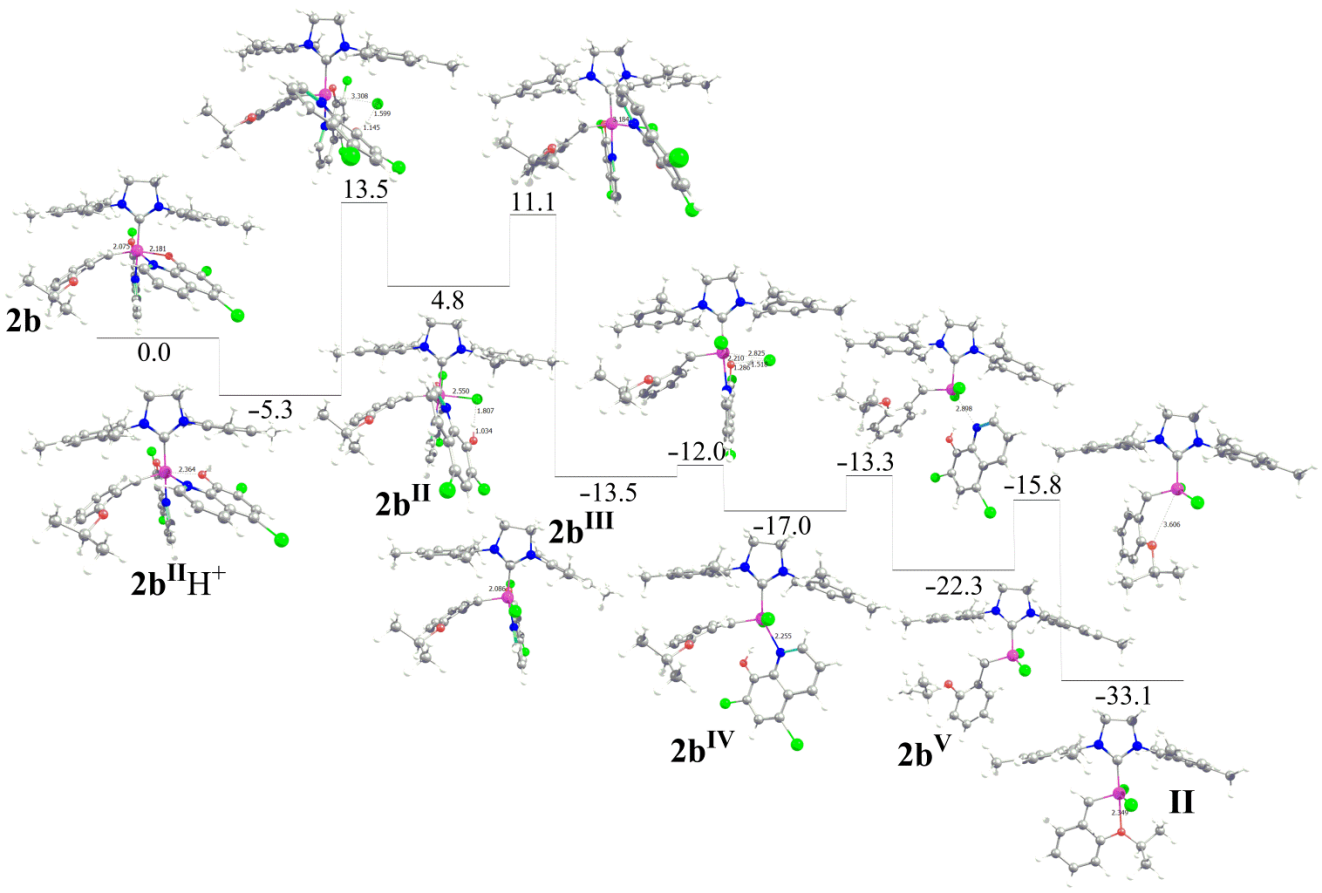
Reaction pathway of the transformation of **2b** to **HovII** (energies in kcal/mol; main distances in Å).

Having established a possible entry point to the activation of **2b** by HCl the whole reaction pathway leading to the conversion of **2b** to a classical Hoveyda type complex was investigated (see Figure 4). After protonation of the O atom *trans* to the alkylidene ligand, a chloride anion could dissociate the Ru–OH bond through transition state **2b^II^H^+^ → 2b^II^**, with displacement of the –OH group and coordination of the chloride *trans* to the alkylidene group. The next step corresponds to a rotation of the chloride ligand from the coordination position *trans* to alkylidene ligand to a coordination position *cis* to both the alkylidene and the SIMes ligands. This rearrangement requires dissociation of the quinolinolate N atom and the complete release of a neutral quinolinolate type ligand, leading to **2b^III^**, which is 13.5 kcal/mol below in energy compared to **2b**.

Protonation of the second O atom is disfavored by 7 kcal/mol. For this reason, the search for a transition state in which the protonation of **2b^III^** occurs by a HCl molecule through a concerted transition state in which the proton of HCl protonates the oxygen of the quinolinolate while the chloride coordinates *trans* to the ylidene group was emphasized. This concerted transition state costs only 1.5 kcal/mol and thus is favored over protonation followed by Cl^−^ coordination. The final product is **2b^IV^**, 17.0 kcal/mol below **2b**, with the attacking Cl atom *trans* to the Ru–alkylidene bond. A direct HCl attack to **2b** via a concerted transition state is not possible, since the Ru center of **2b** has no vacant coordination position. Back to the second protonation step, **2b^IV^** evolves to **2b^V^** through a shift of the second Cl atom from the coordination position *trans* to the Ru–alkylidene bond to reach a geometry with a *trans* disposition of the two Ru–Cl bonds. Species **2b^V^** corresponds to a 14 e^−^ species that can be formed by dissociation of the isopropoxy group from the classical Hoveyda catalyst **II**, which can be obtained by **2b^V^** through coordination of the isopropoxy group. The overall energy balance for the transformation of **2b** to **II** is 33.1 kcal/mol down in energy. Similar energy profiles, corresponding to the transformation of **2a^I^H^+^**, **2a^II^H^+^**, and **2b^I^H^+^** into **II** are reported in Supporting Information File 1, where also the protonation of the N atom of the quinolinolate ligand are explored.

Due to the latent character of the pre-initiators, they may be suitable candidates for the polymerization of very active strained monomers such as DCPD. Another benefit for their use in the polymerization of DCPD is their outstanding solubility in the neat monomer. The main challenge of the polymerization of DCPD is to guarantee an adequate mixing of monomer and catalyst to obtain a homogenous reaction mixture and moreover a steady polymerization product. DCPD is also prone to undergo a retro-Diels–Alder reaction at higher temperatures, causing a mass loss during polymerization if higher temperatures are applied for the reaction.

To test the pre-catalysts regarding their performance in the polymerization of DCPD, two different test-reactions were carried out: a) STA measurements to gain an insight into the course of polymerization and b) tensile strength tests to characterize the obtained polymers.

**Figure 5:**
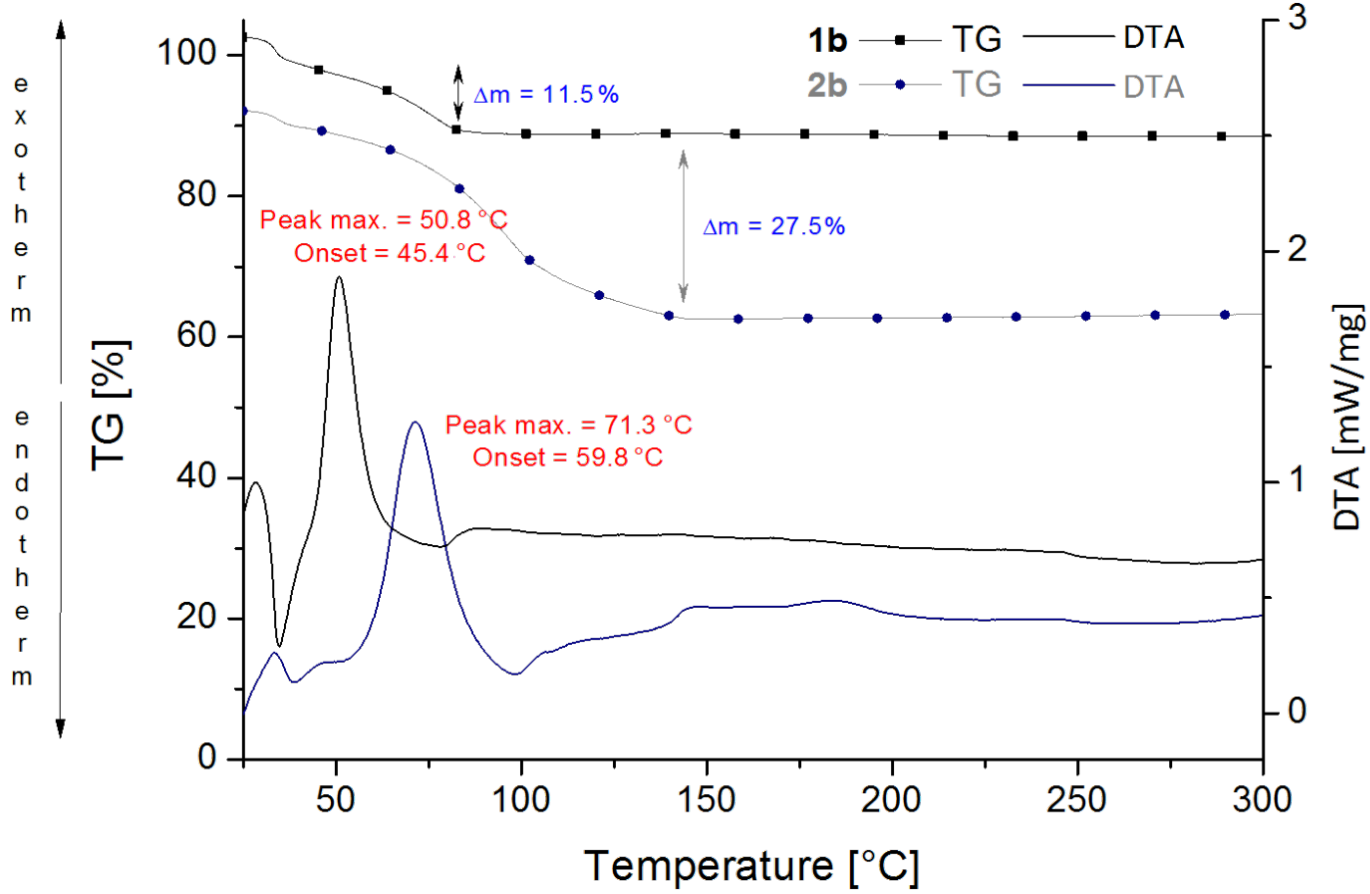
DTA-TGA measurements for polymerizations of DCPD with catalysts **1b** and **2b**; Reaction conditions: [catalyst]:[DCPD]:[HCl]: 1:10.000:25; Temperature program: 3 °C/min.

In the STA plot (see Supporting Information File 1 for further details), representative examples for a successful, a partly successful and an unsuccessful DCPD polymerization are shown. **2a** and **3** totally fail the polymerization of DCPD. The monomer decomposes completely before any curing occurs. On the other hand, **1b** and **2b** polymerize DCPD which can be seen by the exothermic peaks on the left hand side of Figure 5. **2b** requires more time to start the polymerization which causes a higher mass loss of the monomer. **1a** shows some curing, but the main part of the monomer decomposes before it can be polymerized. The exothermic peak of the polymerization merges into the endothermic peak of the monomer decomposition.

Additionally, pDCPD shoulder test bars were made and trialled in a Tensile Strength Test. Tensile strength values (*R*_m_) and the Young’s modulus (ε) were determined and used for comparison with literature data. The most reactive complexes **2b** and **4** exhibit the highest *R*_m_ and Young’s Modulus values (see Table 2), which exceed data taken from literature. This indicates a higher cross linking density of the test specimens.

**Table 2:** Tensile test values for shoulder test bars initiated with complexes **1**–**4**.

Initiator	*E* [MPa]	*R*_m_ [MPa]

**1a**	2137	25.2
**1b**^a^	–	–
**2a**	–	
**2b**	2635	43.8
**3**	1277	20.7
**4**	2664	52.3
comparative example [53]	1870–1980	43.0–46.8

^a^Unexpectedly all attempts to produce shoulder test bars failed.

## Conclusion

In conclusion we were able to synthesize a new family of N,O-chelating initiators bearing 5,7-dihalide-hydroxyquinoline co-ligands. We showed that it is possible to synthesize these initiators using starting complexes bearing different carbene- and different NHC ligands. At the example of initiators **2a** and **2b** it was shown that the arrangement of the ligands around the ruthenium centre can drastically influence the activity of metathesis initiators. Just by changing the positioning of one of the two quinolinolate ligands the catalytic activity is decreased manifoldly.

Even though since the 1980s thousands of papers have presented and described the olefin metathesis catalysis [54], neither the location of a right catalyst for any metathesis reaction [55–56], nor the recipe to rationalize the behaviour of a given catalyst have been fulfilled [57–59]. Thus this study opens a door to find out new families of olefin metathesis catalysts [60], overcoming the issue of bearing a phosphine or to break a Ru–O bond in the precatalysts [55,61].

The initiators exhibit an excellent stability in the solid state as well as in solution and an outstanding latency towards cyclic olefins. The new pre-catalysts can be triggered using HCl. Due to their extremely good solubility in apolar solvents and substrates they are useful candidates for solvent-free polymerizations. Their latency also makes them very suitable for the polymerization of strained monomers such as DCPD.

## Experimental

Unless otherwise noted, all reactions were carried out under nitrogen atmosphere in pre-dried glass ware using Schlenk technique. Materials were purchased from commercially available sources such as Aldrich, Fluka or Alfa Aesar and used without further purification. Complexes **M31** and **M32** were obtained from Umicore. Complex **HovII** was prepared according to literature [46]. Monomer **5** (dimethyl bicyclo[2.2.1]hept-5-ene-2,3-dicarboxylate) was synthesized according to literature [62]. CH_2_Cl_2_ was degassed with nitrogen. Column chromatography was performed on Merck silica gel 60, 230–400 mesh. TLC was performed on aluminum sheets, Merck 60F 254. NMR (^1^H, ^13^C) spectra were recorded on a Bruker Avance 300 MHz or an INOVA 500 MHz spectrometer, in CDCl_3_ as the solvent. The solvent peak of residual CHCl_3_ was used for referencing the NMR spectra to 7.26 (^1^H) and 77.16 ppm (^13^C), respectively. Gel permeation chromatography was used to determine molecular weights and the polydispersity index (PDI). The measurements were run in THF against a polystyrene standard using following arrangement: a Merck Hitachi L6000 pump, separation columns of Polymer Standards Service (5 µm grade size) and a refractive-index detector from Wyatt Technology. X-ray measurements were performed on a Bruker AXS Kappa APEX II diffractometer. The structure was solved by direct methods using SHELXS and refined with SHELXL. The absorption correction was performed using the program SADABS. DTA/TG measurements were done using a NETZSCH STA 449 C with a temperature program of 3 °C/min. The TGA is operated with a helium flow rate of 50 mL/min used in combination with a protective flow of 8 mL/min. Tensile stress tests were performed using a Shimadzu tensile stress test machine. The strain rate for the analysis was set with 1 mm/min. The tested shoulder test bars had a diameter of 37.4 mm and a length of 80 mm.

### General procedure for the preparation of Ru complexes

In a Schlenk flask the corresponding starting material (1 equiv) was dissolved in degassed CH_2_Cl_2_. 5,7-Dihalide-8-hydroxyquinoline (20 equiv) and Cs_2_CO_3_ (20 equiv) were added. The reaction mixture was stirred under an atmosphere of argon for 12 h at 25 °C. Insoluble components were removed by filtration over celite. Column chromatography (silica gel) using cyclohexane/ethylacetate = 10/1 (v/v) yielded the corresponding complexes. The synthesis of the following Ru-based complexes belongs to a patent application [63].

**Chloro-(κ^2^-(*N*,*O*)-5,7-dichloro-8-quinolinolate)-(3-phenyl-1-indenylidene)-(1,3-bis(2,4,6-trimethylphenyl)-4,5-dihydroimidazol-2-ylidene)ruthenium** (**1a**). Complex **1a** was prepared according to the general procedure given above, using **M31** (142 mg, 0.189 mmol), 5,7-dichloro-8-hydroxyquinoline (810 mg, 3.785 mmol) and Cs_2_CO_3_ (1.24 g, 3.815 mmol) as the starting materials. CH_2_Cl_2_ (18 mL) was used as the solvent. Chromatographic work-up gave **1a** in pure form. Yield: 117 mg (70%). Anal. calcd for C_45_H_40_Cl_3_N_3_ORu: C, 63.87; H, 4.76; N, 4.97; found: C, 64.01; H, 4.89; N, 5.01; ^1^H NMR (δ, 20 °C, CDCl_3_, 300 MHz) 8.0 (d, *J* = 8.1 Hz, 1H), 7.97 (d, *J* = 4.8 Hz, 1H), 7.74 (d, *J* = 8.1 Hz), 7.30 (dd, 2H), 7.23 (d, 2H), 7.12 (d, *J* = 7.14 Hz, 1H), 7.05 (s, 2H), 7.03 (m, 1H), 6.44 (s, 3H), 6.23 (s, 2H), 6.20 (d, 1H), 3.87 (s, 4H), 2,32, 2.08, 1.92 (s, 18H); ^13^C NMR (δ, 20 °C, CDCl_3_, 75 MHz) Ru=C and Ru-C not observed, 167.2, 164.6, 146.7, 143.8, 143.2, 142.8, 141.8, 138.0, 136.9, 136.8, 136.5, 133.2, 132.7, 129.1, 129.1, 128.7, 128.0, 127.9, 127.6, 126.0, 125.9, 125.7, 121.8, 121.0, 118.9, 118.5, 117.6, 111.7, 109.3, 51.6, 20.9, 18.1.

**(OC-6-32)-Bis(κ^2^-(N,O)-5,7-dichloro-8-quinolinolate)-(3-phenyl-1-indenylidene)-(1,3-bis(2,4,6-trimethylphenyl)-4,5-dihydroimidazol-2-ylidene)ruthenium** (**1b**). Complex **1b** was isolated from the same experiment than **1a** using column chromatography. Yield 8.4 mg (20%). Anal. calcd for C_54_H_44_Cl_4_N_4_O_2_Ru: C, 63.35; H, 4.33; N, 5.47; found: C, 63.45; H, 4.56; N, 5.76; ^1^H NMR (δ, 20 °C, CDCl_3_, 300 MHz) 8.15 (d, *J* = 4.8 Hz, 1H), 7.99 (dd, *J* = 8.4 Hz, 1H), 7.9 (3H), 7.60 (1H), 7.52 (1H), 7.47 (1H), 7.31 (s, 1H), 7.24 (s, 1H), 7.2 (m, 2H), 6.81 (m, 1H), 6.65 (t, 1H), 6.53 (s, 2H), 6.50 (dd, 1H), 6.35 (s, 2H), 6.28 (d, *J* = 7.2 Hz), 5.48 (dd, *J* = 4.8 Hz, 1H), 3.89 (s, 4H), 2.36, 2.28, 2.08 (s, 18H); ^13^C NMR (δ, 20 °C, CDCl_3_, 75 MHz) Ru=C not observed, 204.3 (1C, Ru-NHC), 166.3, 161.2, 150.0, 145.8, 145.1, 143.1, 142.0, 141.7, 140.4, 137.9, 137.5, 137.3, 136.8, 136.4, 136.3, 136.4, 136.3, 136.2, 133.3, 133.2, 130.0, 129.7, 129.6, 129.4, 129.3, 129.1, 128.5, 128.2, 128.1, 126.6, 126.0, 125.9, 125.8, 125.4, 120.9, 120.7, 120.1, 118.5, 118.2, 112.2, 108.2, 53.3, 20.9, 20.3, 19.5.

**(OC-6-14)-Bis(κ^2^-(N,O)-5,7-dichloro-8-quinolinolate)-(2-isopropylbenzylidene)-(1,3-bis(2,6-diisopropylphenyl)-4,5-dihydroimidazol-2-ylidene)ruthenium** (**2a**). Complex **2a** was prepared according to the general procedure given above, using **HovII** (106 mg, 0.169 mmol), 5,7-dichloro-8-hydroxyquinoline (707 mg, 3.303 mmol) and Cs_2_CO_3_ (881 mg, 2.704 mmol) as the starting materials. CH_2_Cl_2_ (18 mL) was used as the solvent. Chromatographic work-up gave **2a** in pure form. Yield: 46.5 mg (28%). ^1^H NMR (δ, 20 °C, CDCl_3_, 300 MHz) 19.10 (s, 1H), 8.09 (d, *J* = 4.04 Hz, 1H), 7.95 (d, *J* = 8.56 Hz, *J* = 1.43 Hz, 1H), 7.68 (d, *J* = 8.43 Hz, *J* = 1.30 Hz, 1H), 7.49 (s, 1H), 7.17 (s, 1H), 7.05 (m, 2H), 6.56 (d, *J* = 8.04 Hz, 1H), 6.48 (s, 2H), 6.43 6,39 (m, 2H), 6.14 (s, 2H), 6.06 (m, 2H), 3.97 (m, 5H), 2.45 (s, 6H), 2.27 (s, 6H), 1.90 (s, 6H), 1.43 (d, 3H), 1.05 (d, 3H); ^13^C NMR (δ, 20 °C, CDCl_3_, 75 MHz) 315.5 (1C, Ru=CH), Ru-NHC not observed, 162.6, 161.3, 149.7, 149.4, 149.0, 144.2, 143.2, 142.4, 142.3, 138.1, 136.9, 136.6 135.8, 132.3, 131.7, 129.3, 129.2, 128.7, 127.7, 126.2, 125.8, 125.7, 122.2, 121.6, 121.0, 119.5, 118.9, 112.0, 109.2, 76.2, 51.6, 23.1, 21.5, 20.8, 18.8, 18.5.

**(OC-6-32)-Bis(κ^2^-(N,O)-5,7-dichloro-8-quinolinolate)-(2-isopropylbenzylidene)-(1,3-bis(2,6-diisopropylphenyl)-4,5-dihydroimidazol-2-ylidene)ruthenium** (**2b**). Complex **2b** was isolated from the same experiment than **2a** using column chromatography. Yield 91 mg (57%). Anal. calcd for C_54_H_44_Cl_4_N_4_O_2_Ru: C, 63.51; H, 4.25; N, 5.48; found: C, 60.19; H, 4.86; N, 5.88; ^1^H NMR (δ, 20 °C, CDCl_3_, 300 MHz) 18.24 (bs, 1H), 9.00 (d, *J* = 4.67 Hz, 1H), 8.09 (d, *J* = 8.56 Hz, 1H), 7.83 (d, *J* = 8.30 Hz, 1H) 7.57 (s, 1H), 7.12 (s, 1H), 7.06 (m, 1H), 6.94 (m, 1H), 6.59 (s, 2H), 6.39 (d, 1H), 6.26 (s, 2H), (d, 1H), (m, 1H), 5.98 (m, 1H), 5.32 (d, *J* = 4.54 Hz), 4.54 (m, 1H), 3.92 (q, 4H), 2.57 (s, 6H), 2.04 (s, 6H), 1.91 (s, 6H), 1.53 (d, 3H), 1.31 (d, 3H); ^13^C NMR (δ, 20 °C, CDCl_3_, 75 MHz) Ru=C not observed, 209.5 (1C, Ru-NHC), 166.4, 160.9, 147.7, 146.7, 147.1, 146.7, 164.5, 146.5, 144.9, 141.2, 137.1, 137.0, 136.7, 136.5, 119.3, 125.8, 132.7, 132.2, 129.2, 129.1, 129.0, 128.6, 127.9, 126.4, 120.7, 120.1, 119.7, 118.0, 111.3, 110.5, 106.4, 68.7, 51.7, 22.7, 22.3, 20.9, 18.9, 18.1.

**Chloro-(κ^2^-(N,O)-5,7-dibromo-8-quinolinolate)-(3-phenyl-1-indenylidene)-(1,3-bis(2,4,6-trimethylphenyl)-4,5-dihydroimidazol-2-ylidene)ruthenium** (**3**). Complex **3** was prepared according to the general procedure given above, using **M31** (160 mg, 0.214 mmol), 5,7-dibromo-8-hydroxyquinoline (960 mg, 3.169 mmol) and Cs_2_CO_3_ (1.00 g, 3.077 mmol) as the starting materials. Diethyl ether (20 mL) was used as the solvent. Chromatographic work-up gave **3** in pure form. Yield: 50 mg (25%). Anal. calcd for C_45_H_40_Br_2_ClN_3_ORu: C, 57.80; H, 4.31; N, 4.49; found: C, 57.89; H, 4.32; N, 4.72; ^1^H NMR (δ, 20 °C, CDCl_3_, 300 MHz) 7.97 (d, *J* = 8.52, 1H), 7.90 (d, *J* = 4.26 Hz, 1H), 7.81 (s, 1H), 7.71 (d, *J* = 8.33 Hz, 1H), 7.57 (s, 1H), 7.53 (bs, 3H), 7.34 (bs, 3H, CH), 7.11 (d, *J* = 7.00 Hz, 1H), 7.03 (q, 1H, CH), 6.49 (bs, 1H), 6.42 (s, 3H), 6.27 (s, 2H), 6.18 (1H, bs), 3.89 (s, 4H), 2.30 (s, 6H), 2.15 (bs, 6H), 1.93 (s, 6H); ^13^C NMR (δ, 20 °C, CDCl_3_, 75 MHz) Ru=C not observed, 241.9 (Ru-NHC), 168.8, 144.5, 144.1, 143.7, 143.0, 137.8, 137.0, 136.9, 136.6, 135.6, 135.2, 134.2, 133.4, 129.2, 129.1, 128.2, 127.8, 127.6, 127.5, 125.9, 122.6, 121.6, 117.4, 108.7, 108.3, 98.2, 51.5 (2C), 21.0, 19.2 (6C).

**(OC-6-32)-Bis(κ^2^-(N,O)-5,7-dichloro-8-quinolinolate)-(3-phenyl-1-indenylidene)-(1,3-bis(2,6-diisopropylphenyl)-4,5-dihydroimidazol-2-ylidene)ruthenium** (**4**). Complex **4** was prepared according to the general procedure given above, using **M32** (58 mg, 0.070 mmol), 5,7-dichloro-8-hydroxyquinoline (136 mg, 0.636 mmol) and Cs_2_CO_3_ (300 mg, 0.923 mmol) as the starting materials. CH_2_Cl_2_ (6 mL) was used as the solvent. Chromatographic work-up gave **4** in pure form. Yield: 40.3 mg (52%). Anal. calcd for C_60_H_56_Cl_4_N_4_O_2_Ru: C, 65.04; H, 5.09; N, 5.06; found: C, 64.95; H, 4.87; N, 5.04; ^1^H NMR (δ, 20 °C, CDCl_3_, 300 MHz) 8.03 (m, 3H), 7.91 (m, 2H), 7.67 (m, 1H), 7.53 (m, 2H), 7.41 (m, 3H), 7.30 (m, 2H), 7.21 (s, 1H), 7.16 (m, 2H), 6.79 (m, 2H), 6.65 (m, 2H), 6.47 (m, 1H), 6.26 (m, 2H), 5.9 (d, *J* = 4.51 Hz, 1H), 4.66, 4.17, 3.9, 3.76, 3.48 (8H), 1.65, 1.34, 1.27, 1.17, 0.91, 0.61, 0.45 (24H); ^13^C NMR (δ, 20 °C, CDCl_3_, 75 MHz) 286.2 (Ru=C), 206.6 (1C, Ru-NHC), 165.4, 162.0, 151.0, 147.4, 146.4, 145.8, 145.6, 145.3, 145.2, 145.1, 144.7, 144.0, 141.2, 141.1, 139.3, 138.4, 137.0, 133.8, 132.6, 130.0, 129.8, 129.7, 128.9, 127.8, 127.7, 127.6, 126.0, 125.8, 125.3, 125.2, 124.8, 124.4, 124.3, 123.1, 121.5, 120.4, 120.0, 117.9, 117.7, 112.6, 108.4, 58.7, 55.9, 29.7, 28.6, 28.5, 27.3, 26.9, 26.1, 25.6, 24.8, 24.6, 22.0, 21.9, 21.2.

### General polymerization procedure

Defined solutions of pre-initators **1**–**4** and **5** (300 equiv, 0.01 mmol/mL) were prepared. The reactions were performed in CH_2_Cl_2_ at room temperature and in toluene for 80 °C polymerizations. Ethereal HCl (50 equiv; HCl relative to ruthenium) was added to activate the reaction. The reaction was followed by TLC (cyclohexane/ethyl acetate = 3:1) and after complete conversion stopped with an excess of ethyl vinyl ether. The Polymer was precipitated in vigorously stirred methanol (approx. 25 mL for 100 mg polymer), and the white to yellowish precipitate was sampled and dried in vacuum.

### Computational details

All calculations were performed with the Gaussian 09 package [64], Revision A.1, at the BP86 GGA level [65–67] using the SDD ECP on Ru [68–70] and the split-valence plus one polarization function SVP basis set on all main group atoms during geometry optimizations [71]. Furthermore diffuse basis sets have been incorporated for O and Cl [72]. The reported energies have been optimized via single point calculations on the BP86 geometries with triple-ζ valence plus polarization (TZVP keyword in Gaussian) using the M06 functional [73]. Solvent effects, dichloromethane, were calculated with the PCM model [74–75], and non-electrostatic terms were also included. The geometry optimizations were performed without symmetry constraints, and the nature of the extrema was checked by analytical frequency calculations.

## Supporting Information

Crystallographic data for complexes **2a** and **2b** have also been deposited with the CCDC, nos. 1439204 and 1439205, and can be obtained free of charge from http://www.ccdc.cam.ac.uk.

File 1 Experimental data, energies, Cartesian coordinates, and 3D view for all DFT optimized species discussed in this work.
