# Supplementary material for: Simple activation by acid of latent Ru-NHC-based metathesis initiators bearing 8-quinolinolate co-ligands
Source: Beilstein J Org Chem. 2016 Jan 28;12:154–65. doi: 10.3762/bjoc.12.17 (PMC4734425; doi:10.3762/bjoc.12.17)
Supplement: File 1 — Experimental data, energies, Cartesian coordinates, and 3D view for all DFT optimized species discussed in this work. [file Beilstein_J_Org_Chem-12-154-s001.pdf]

Supporting Information

for

Simple activation by acid of latent Ru-NHC-based metathesis initiators bearing 8-quinolinolate co-ligands

Julia Wappel[1], Roland C. Fischer[2], Luigi Cavallo[3], Christian Slugovc[1] and Albert Poater[3,4,*]

Address: [1]Institute for Chemistry and Technology of Materials, Graz University of Technology, NAWI Graz, Stremayrgasse 9, 8010 Graz, Austria, [2]Institute of Inorganic Chemistry, Graz University of Technology, Stremayrgasse 9, 8010 Graz, Austria, [3]KAUST Catalysis Center, Physical Sciences and Engineering Division, King Abdullah University of Science and Technology, Thuwal 23955-6900, Saudi Arabia and [4]Institut de Química Computacional i Catàlisi, Departament de Química, Universitat de Girona, Campus de Montilivi, E-17071 Girona, Spain.

Email: Albert Poater - albert.poater@udg.edu

*Corresponding author

Experimental data, energies, Cartesian coordinates, and 3D view for all DFT optimized species discussed in this work

1. Complex synthesis and characterization

Synthesis way A

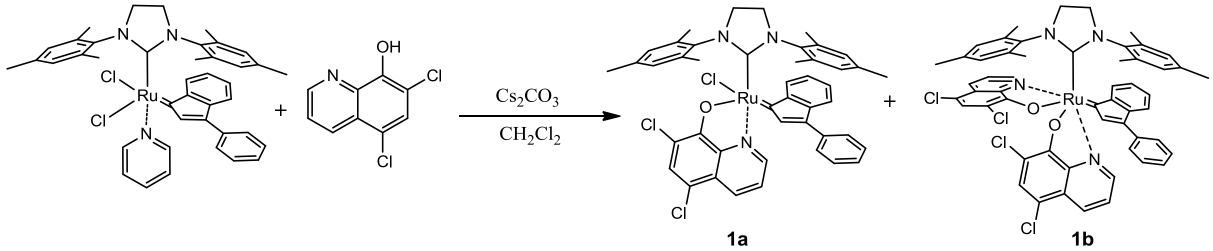

In a Schlenk flask **M31** (Py)(H_2IMes)(Cl_2)Ru(3-phenyl-1*H*-inden-1-ylidene) (142 mg, 0.189 mmol, 1 equiv) was dissolved in degassed CH_2Cl_2 (≈18 mL). 5,7-Dichloro-8-hydroxyquinoline (810 mg, 3.785 mmol, 20 equiv) and Cs_2CO_3 (1,24 g, 3.815 mmol, 20 equiv) were added. The reaction mixture was stirred in a Schlenk flask under argon atmosphere overnight. The insoluble residue was filtered over celite. According to a TLC (CH/EE 5:1) two derivatives were formed. The two catalysts were separated via column chromatography (CH/EE 10:1) and fully characterized by NMR. Yield = 90% (117 mg **1** and 33 mg **2**).

1a: **^{1}H-NMR** (δ, 20°C, $CDCl_3$, 300 MHz): 8.0 (d j= 8.1 Hz, 1H, $CH^{hq\ 3}$), 7.97 (d j= 4.8 Hz, 1H, $CH^{hq\ 1}$), 7.74 (d j= 8.1 Hz, CH), 7.30 (t, 2H, CH), 7.05 (s, 2H, CH), 7.23 (d, 2H, CH), 7.12 (d j= 7.14 Hz, 1H, CH), 7.03 (q, 1H, $CH^{hq\ 2}$), 6.44 (s, 3H CH), 6.23 (s, 2H, CH), 6.20 (d, 1H, CH), 3.87 (s, 4H, CH_2), 2, 32, 2.08, 1.92 (s, 18H, $CH_3^{mes\ 7,7',8,8',9,9'}$).

^{13}C-NMR (δ, 20°C, $CDCl_3$, 300 MHz): Ru=C and Ru-C not observed, 167.2 (C_q), 164.6 (CH), 146.7 (CH), 143.8 (C_q), 143.2 (CH), 142.8 (CH), 141.8 (C_q), 138.0 (C_q), 136.9 (C_q), 136.8 (C_q), 136.5 (C_q), 133.2 (CH), 132.7 (CH), 129.1 (CH), 129.1 (CH), 128.7 (CH), 128.0 (CH), 127.9 (CH), 127.6 (CH), 126.0 (C_q), 125.9 (CH), 125.7 (C_q), 121.8 (CH), 121.0 (CH), 118.9 (C_q), 118.5 (C_q), 117.6 (CH), 111.7 (C_q), 109.3 (C_q), 51.6 (2C, CH_2-N), 20.9, 18.1 (12C, CH_3).

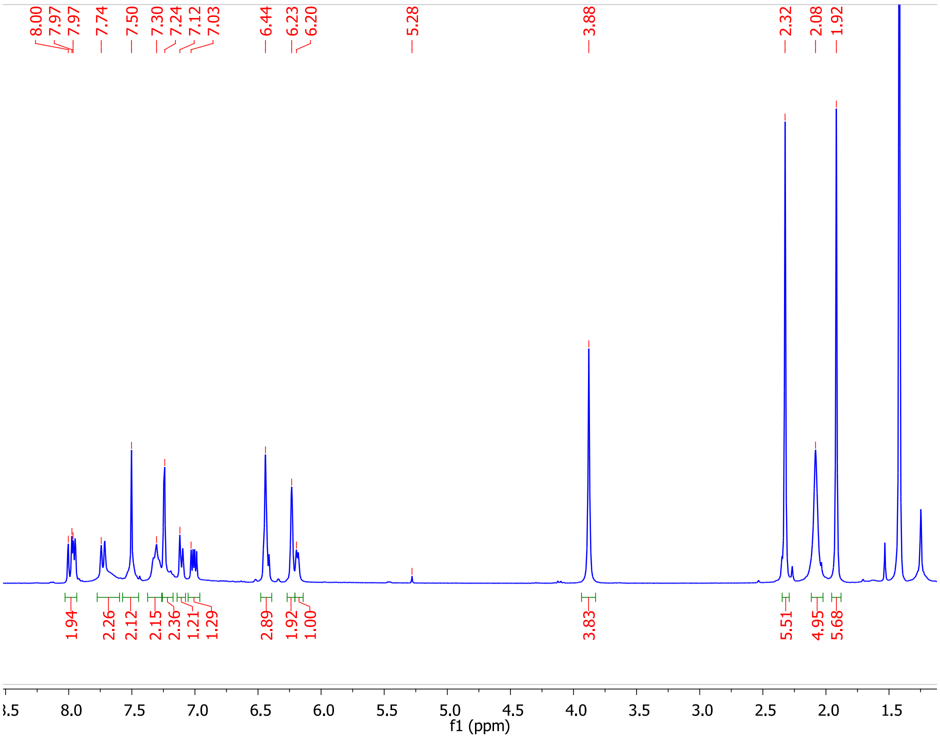

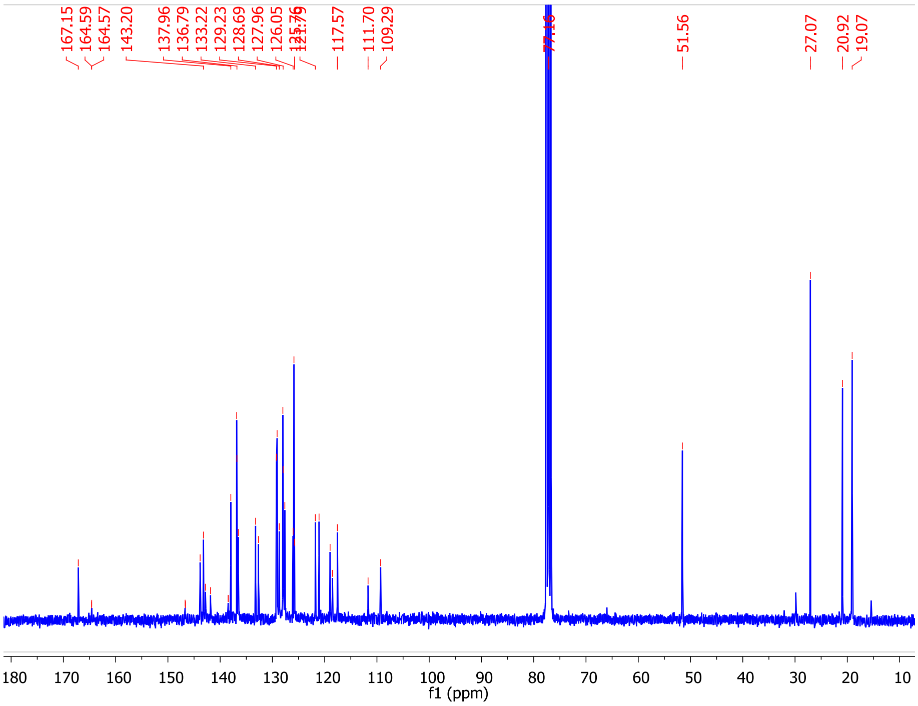

1b: **^{1}H-NMR** (δ, 20°C, $CDCl_3$, 300 MHz): 8.15 (d j= 4.8 hZ, 1H, $CH^{hq\ 1}$), 7.99 (dd j= 8.4, 1H, $CH^{hq\ 3}$), 7.9 (3H, $CH^{hq\ 3}$), 7.60 (1H, CH), 7.52 (1H, CH), 7.47 (1H, CH), 7.31 (s, 1H, CH), 7.24 (s, 1H, CH), 7.2 (t, 2H, $CH^{ind\ 2\ or\ 3}$), 6.81 (q, 1H, $CH^{hq\ 2}$), 6.65 (t, 1H, $CH^{ind\ 2\ or\ 3}$), 6.53 (s, 2H, CH^{mes}), 6.50 (q, 1H, $CH^{hq\ 2}$)6.35 (s, 2H, CH^{mes}), 6.28 (d j=7.2 Hz, $CH^{ind\ 1\ or\ 4}$), 5.48 (dd j=4.8 Hz, 1H, $CH^{hq\ 1}$), 3.89 (s, 4H, $CH_2{}^{mes}$), 2.36, 2.28, 2.08 (s, 18H, $CH_3{}^{mes}$).

^{13}C-NMR (δ, 20°C, $CDCl_3$, 300 MHz): Ru=C not observed, 204.3 (1C, C_q, Ru-C), 166.3 (C_q), 161.2 (C_q), 150.0 (CH), 145.8 (C_q), 145.1 (C_q), 143.1 (CH), 142.0 (C_q), 141.7 (CH), 140.4 (C_q),

137.9 (C_q), 137.5 (C_q), 137.3 (C_q), 136.8 (C_q), 136.4 (C_q), 136.3 (C_q), 136.4 (C_q), 136.3 (C_q), 136.2 (C_q), 133.3 (CH), 133.2 (CH), 130.0 (CH), 129.7 (CH), 129.6 (CH), 129.4 (CH), 129.3 (CH), 129.1 (CH), 128.5 (CH), 128.2 (CH), 128.1 (CH), 126.6 (CH), 126.0 (CH), 125.9 (C_q), 125.8 (CH), 125.4 (C_q), 120.9 (CH), 120.7 (CH), 120.1 (C_q), 118.5 (CH), 118.2 (C_q), 112.2 (C_q), 108.2 (C_q), 53.3 (2C, CH_2-N), 20.9, 20.3, 19.5 (18C, CH_3).

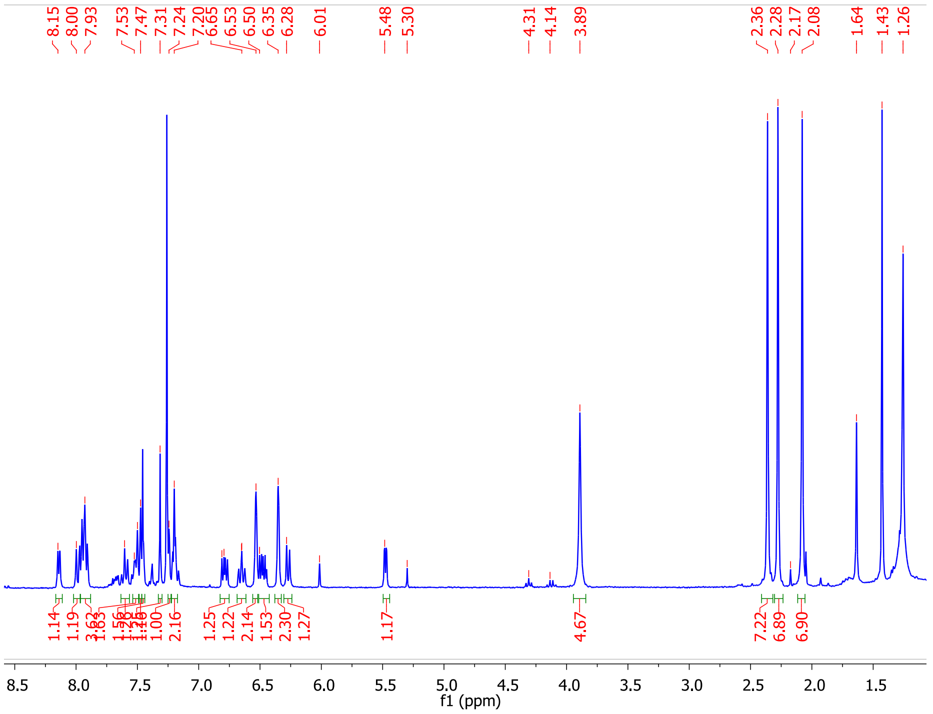

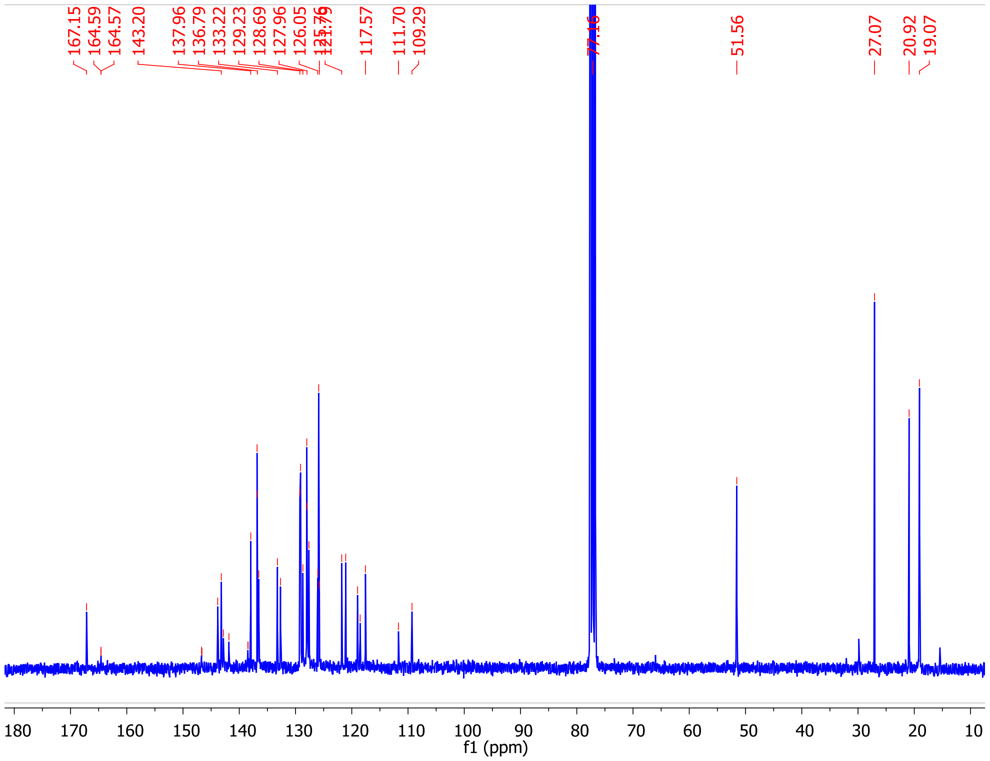

Synthesis way B

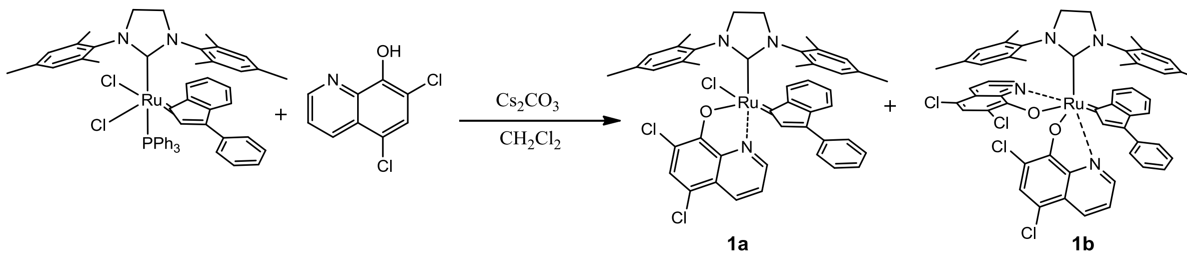

1a **1b**

In a Schlenk flask (PPh_3)(H_2IMes)(Cl_2)Ru(3-phenyl-1*H*-inden-1-ylidene) (26 mg, 0,0278 mmol, 1 equiv) was dissolved in degassed CH_2Cl_2 (≈3 mL). 5,7-Dichloro-8-hydroxyquinoline (88 mg, 0,411 mmol, 15 equiv) and Cs_2CO_3 (150 mg, 0,461 mmol, 16 equiv) were added. The reaction mixture was stirred in a Schlenk flask under argon atmosphere overnight. The insoluble residue was filtered over celite. According to a TLC (CH/EE 5:1) two derivatives were formed. The two catalysts were separated via column chromatography (CH/EE 20:1) and fully characterized by NMR. Yield = 73% (10,3 mg **1a** and 8,4 mg **1b**).

Di-(5,7-dichloro-8-quinolinolate)(isopropylbenzylidene)(1,3-bis(2,4,6-trimethylphenyl)-4,5-dihydroimidazol-2-ylidene)ruthenium, 2a and 2b

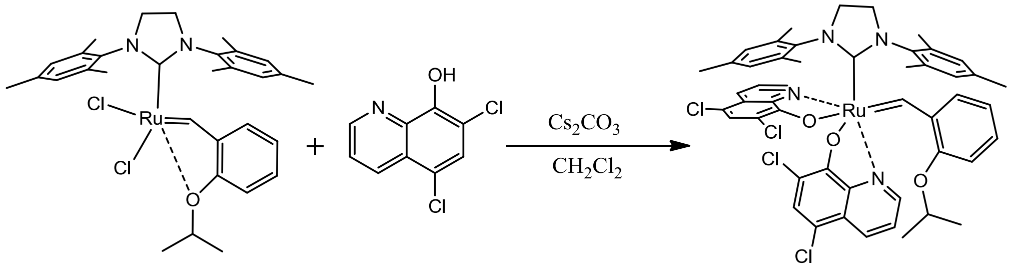

2 Derivatives
Derivative 1: **1**
Derivative 2: **2**

In a Schlenk flask, (H_2IMes)Cl_2Ru(CH-*o*-OiPrC_6H_4) (106 mg, 0.169 mmol, 1 equiv) was dissolved in degassed CH_2Cl_2 (≈18 mL). 5,7-Dichloro-8-hydroxyquinoline (707 mg, 3.303 mmol, 19 equiv) and Cs_2CO_3 (150 mg, 0,461 mmol, 16 equiv) were added. The reaction mixture was stirred under a nitrogen atmosphere overnight. The insoluble residue was filtered over celite. According to a TLC (CH/EE 5:1) two derivatives were formed. The products were separated via column chromatography (CH/EE 5:1) and fully characterized by NMR and crystal structure analysis. Yield = 83% (46.5 mg **2a** and 91 mg **2b**).

2a: **^{1}H-NMR** (δ, 20°C, $CDCl_3$, 300 MHz): 19.10 (s, 1H, Ru=C*H*), 8.09 (d J=4,04, 1H, CH^{hq}), 7.95 (d J=8,56; j=1,43, 1H, CH^{hq}), 7.68 (d J=8,43 j=1,30, 1H, CH^{ph}), 7.49 (s, 1H, CH^{hq}), 7.17 (s, 1H, CH^{hq}), 7.05 (m, 2H, CH^{hq}), 6.56 (d J=8,04, 1H, CH^{hq}), 6.48 (s, 2H, CH^{mes}), 6.43 6,39 (?, 2H,

CH^{ph}), 6.14 (s, 2H, CH^{mes}), 6.06 (2H, C$H^{hq + ph}$), 3.97 (5H, CH_2 + C$H^{isoprop}$), 2.45 (s, 6H), 2.27 (s, 6H), 1.90 (s, 6H, C$H_3^{1, 1', 2, 2', 3, 3'}$), 1.43 (d, 3H, C$H_3^{isprop}$), 1.05 (d, 3H, C$H_3^{isoprop}$).

^{13}C-NMR (δ, 20°C, $CDCl_3$, 75 MHz): 315.5 (1C, Ru=CH), Ru-C was not observed, 162.6, 161.3, 149.7, 149.4, 149.0, 144.2, 143.2, 142.4, 142.3, 138.1 (C_q), 136.9 (C_q), 136.6 (C_q), 135.8 (C_q), 132.3 (CH), 131.7 (CH), 129.3 (CH), 129.2 (CH), 128.7, 127.7 (CH), 126.2, 125.8, 125.7, 122.2 (CH), 121.6 (CH), 121.0 (CH), 119.5 (CH), 118.9, 112.0, 109.2, 76.2 (1C, $CH^{isoprop}$), 51.6 (2C, CH_2-N), 23.1 (1C, $CH_3^{isoprop}$), 21.5 (1C, $CH_3^{isoprop}$), 20.8, 18.8, 18.5 (2C, $CH_3^{mes\ 7, 7', 8, 8', 9, 9'}$).

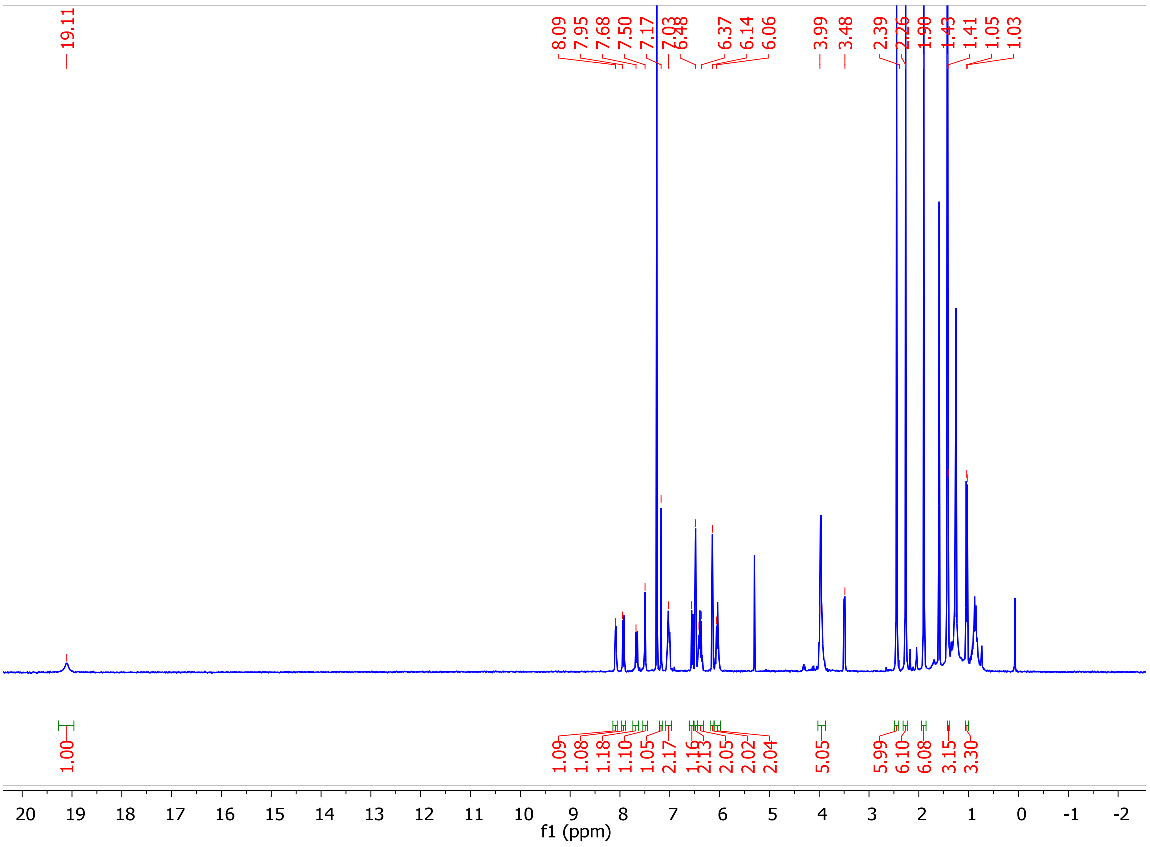

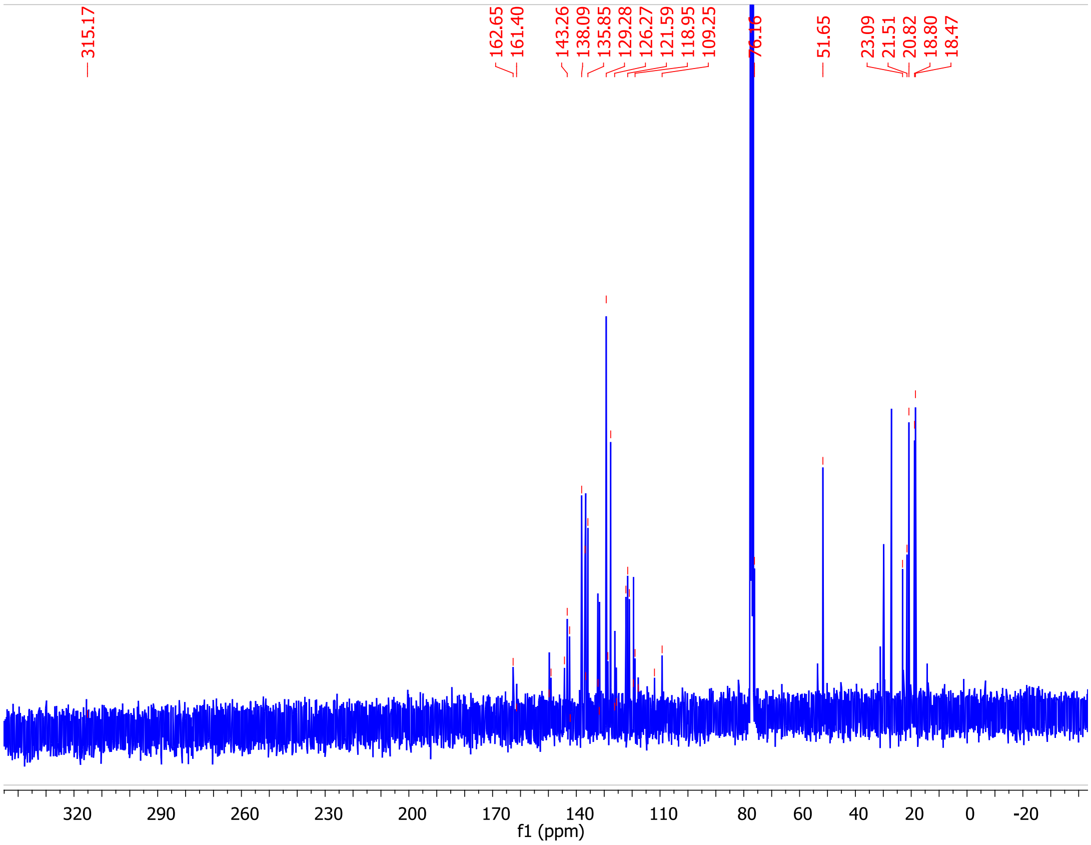

2b: ^{1}**H-NMR** (δ, 20°C, CDCl$_3$, 300 MHz):18.24 (bs, 1H, Ru=CH), 9.00 (d j=4.67 Hz, 1H, CH$^{\text{hq 1}}$), 8.09 (d J=8.56 Hz, 1H, CH$^{\text{hq 3}}$), 7.83 (d J=8.30 Hz, 1H, CH$^{\text{hq 3}}$) 7.57 (s, 1H, CH$^{\text{hq 4 or 4}}$), 7.12 (s, 1H, CH$^{\text{hq 4 or 4}}$), 7.06 (q, 1H, CH$^{\text{hq 2}}$), 6.94 (t, 1h; CH$^{\text{ph 3 or 4}}$), 6.59 (s, 2H, CH$^{\text{mes 3+3' or 5+5'}}$), 6.39 (d, 1H, CH$^{\text{ph 2 or 5}}$), 6.26 (s, 2H, CH$^{\text{mes 3+3' or 5+5'}}$), (d, 1H, CH$^{\text{ph 2 or 5}}$), (t, 1H, Ch$^{\text{hq 2}}$), 5.98 (t, 1H, CH$^{\text{ph 3 or 4}}$), 5.32 (d j= 4.54 Hz, 1H, CH$^{\text{hq 1}}$), 4.54 (m, 1H, CH^{isoprop}), 3.92 (q, 4H, CH$_2^{\text{mes}}$), 2.57 (s, 6H), 2.04 (s, 6H), 1.91 (s, 6H, CH$_3^{\text{mes 7, 7', 8, 8', 9, 9'}}$), 1.53 (d, 3H, CH$_3^{\text{isoprop}}$), 1.31 (d, 3H, CH$_3^{\text{isoprop}}$).

^{13}**C-NMR** (δ, 20°C, CDCl$_3$, 75 MHz): Ru=C not observed, 209.5 (1C, Ru-C), 166.4 (C$_q$), 160.9 (C$_q$), 147.7 (C$_q$), 146.7 (C$_q$), 147.1 (C$_q$), 146.7 (C$_q$), 164.5 (CH), 146.5 (CH), 144.9 (C$_q$), 141.2 (CH), 137.1 (C$_q$), 137.0 (C$_q$), 136.7 (C$_q$), 136.5 (C$_q$), 119.3 (C$_q$), 125.8 (C$_q$), 132.7 (CH), 132.2 (CH), 129.2 (CH), 129.1 (2C, CH), 129.0 (CH), 128.6 (CH), 127.9 (CH), 126.4 (C$_q$), 120.7 (CH), 120.1 (CH), 119.7 (CH), 118.0 (C$_q$), 111.3 (C$_q$), 110.5 (CH), 106.4 (C$_q$), 68.7 (1C, CH^{isoprop}), 51.7 (2C, CH$_2$), 22.7, 22.3 (2C, CH$_3^{\text{isoprop}}$), 20.9, 18.9, 18.1 (6C, CH$_3^{\text{mes 7, 7', 8, 8', 9, 9'}}$).

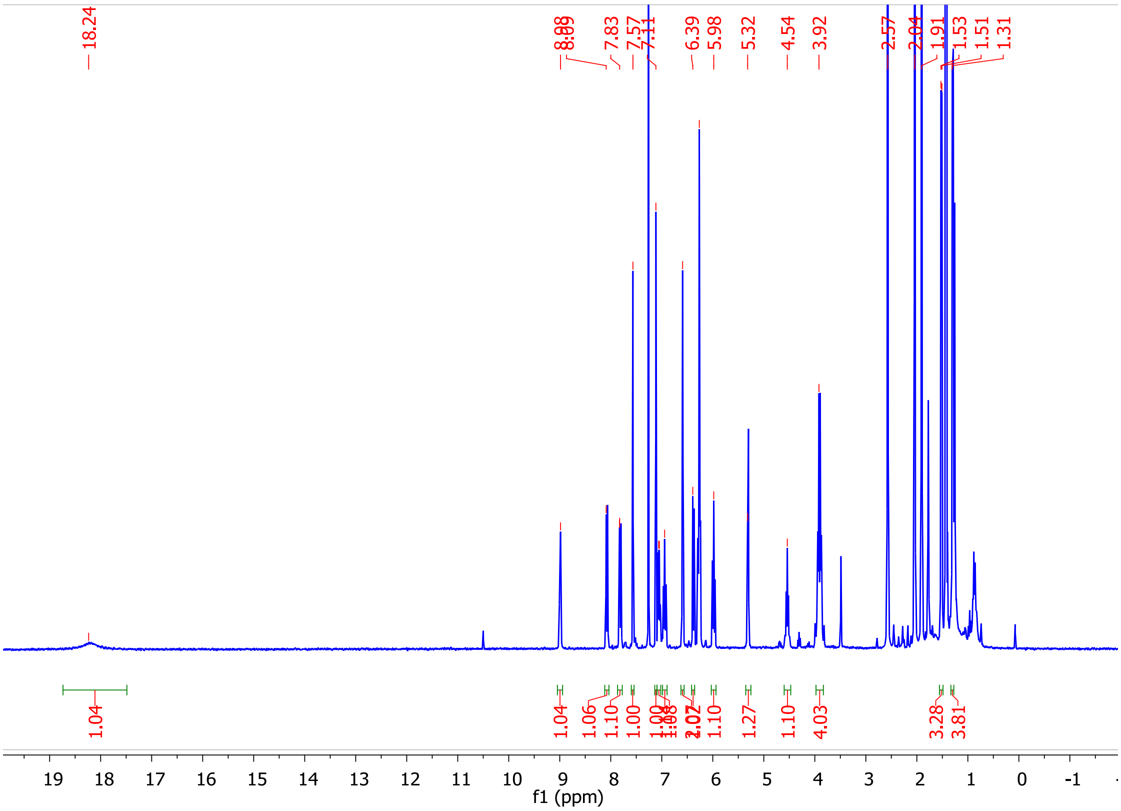
18.24
7.83
7.57
7.11
6.39
5.98
5.32
4.54
3.92
2.57
2.04
1.91
1.53
1.51
1.31
1.04
1.06
1.10
1.00
1.10
1.27
1.10
4.03
3.28
3.81
19 18 17 16 15 14 13 12 11 10 9 8 7 6 5 4 3 2 1 0 -1
f1 (ppm)

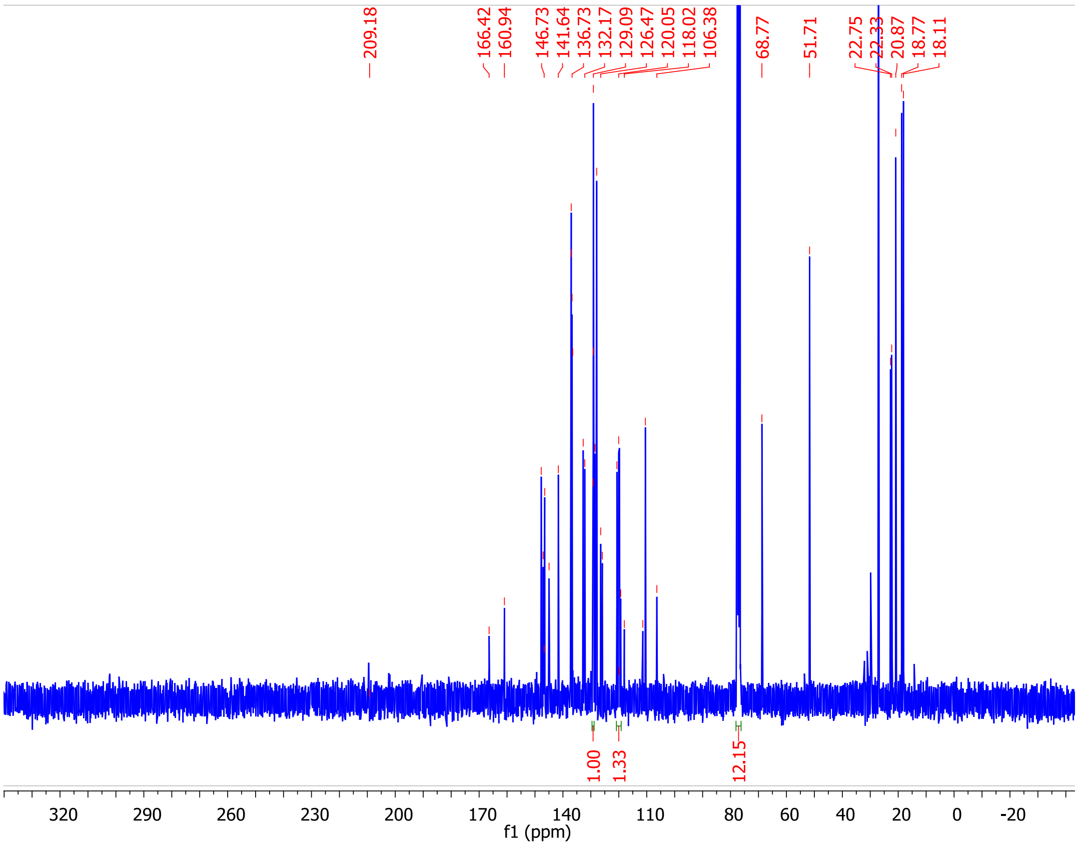
209.18
166.42
160.94
146.73
141.64
136.73
132.17
129.09
126.47
120.05
118.02
106.38
68.77
51.71
22.75
22.33
20.87
18.77
18.11
1.00
1.33
12.15
320 290 260 230 200 170 140 110 80 60 40 20 0 -20
f1 (ppm)

Crystal structures

The crystals for the X-ray diffraction measurement were obtained by slow diffusion of Et_2O in a saturated solution of CH_2Cl_2.

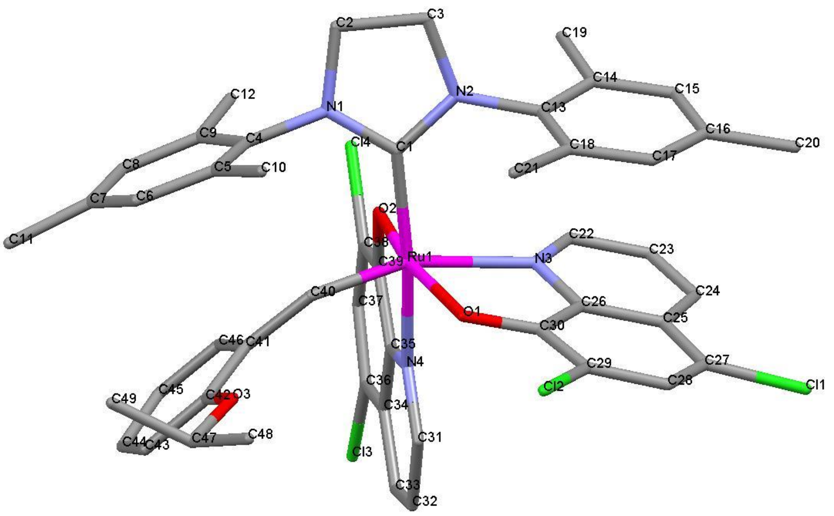

Figure 1: Crystal strucuture of complex 1

Empirical formula	C24.50 H23 Cl2 N2 O1.50 Ru0.50	
Formula weight	490.88	
Temperature	100(2) K	
Wavelength	0.71073 Å	
Crystal system	Triclinic	
Space group	P-1	
Unit cell dimensions	a = 9.1375(5) Å	α= 72.358(3)°.
	b = 12.6359(6) Å	β= 88.303(5)°.
	c = 20.7869(12) Å	γ = 74.605(3)°.
Volume	2201.7(2) $Å^3$	
Z	4	
Density (calculated)	1.481 Mg/m^3	
Absorption coefficient	0.647 mm^{-1}	
F(000)	1008	
Crystal size	0.246 x 0.112 x 0.086 mm^3	
Theta range for data collection	1.74 to 24.05°.	
Index ranges	-10<=h<=10, -14<=k<=14, -23<=l<=23	
Reflections collected	87418	
Independent reflections	6894 [R(int) = 0.1038]	
Completeness to theta = 24.05°	99.0 %	
Absorption correction	None	
Refinement method	Full-matrix least-squares on F^2	

Data / restraints / parameters	6894 / 0 / 558
Goodness-of-fit on F^2	1.154
Final R indices [I>2sigma(I)]	R1 = 0.0528, wR2 = 0.0964
R indices (all data)	R1 = 0.0901, wR2 = 0.1153
Largest diff. peak and hole	0.802 and -0.896 e.Å^{-3}

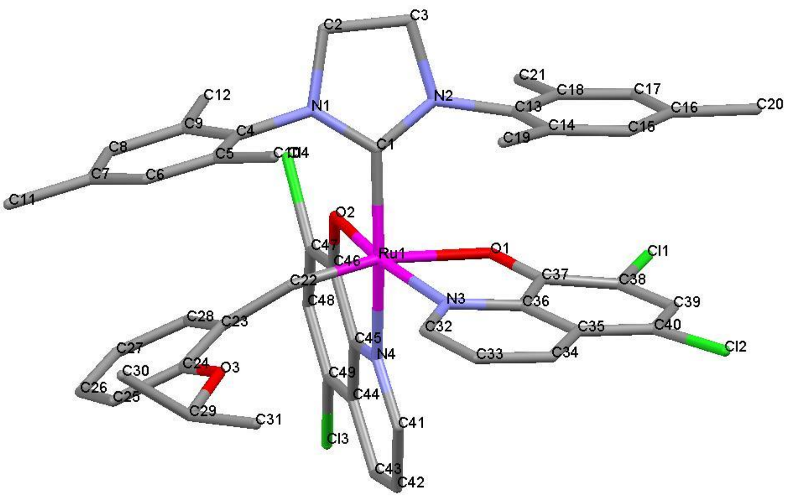

Figure 2: Crystal structure of complex 2

Empirical formula	C49 H47 Cl4 N4 O3 Ru	
Formula weight	982.78	
Temperature	100(2) K	
Wavelength	0.71073 Å	
Crystal system	triclinic	
Space group	P-1	
Unit cell dimensions	a = 11.7075(4) Å	α= 68.854(2)°
	b = 13.0761(4) Å	β= 76.072(2)°
	c = 16.6073(5) Å	γ = 73.544(2)°
Volume	2246.63(12) Å^3	
Z	2	
Density (calculated)	1.453 Mg/m^3	
Absorption coefficient	0.634 mm^{-1}	
F(000)	1010	
Crystal size	0.18 x 0.12 x 0.04 mm^3	
Theta range for data collection	1.71 to 25.00°	

Index ranges	-13<=h<=13, -15<=k<=15, -19<=l<=19
Reflections collected	27470
Independent reflections	7791 [R(int) = 0.0629]
Completeness to theta = 25.00°	98.5 %
Max. and min. transmission	0.9751 and 0.8944
Refinement method	Full-matrix least-squares on F^2
Data / restraints / parameters	7791 / 0 / 558
Goodness-of-fit on F^2	1.013
Final R indices [I>2sigma(I)]	R1 = 0.0727, wR2 = 0.1411
R indices (all data)	R1 = 0.1226, wR2 = 0.1673
Largest diff. peak and hole	1.388 and -1.529 e.$Å^{-3}$

Di-(5,7-dibromo-8-quinolinolate)(isopropylbenzylidene)(1,3-bis(2,4,6-trimethylphenyl) 4,5-dihydroimidazol-2-ylidene)ruthenium (3)

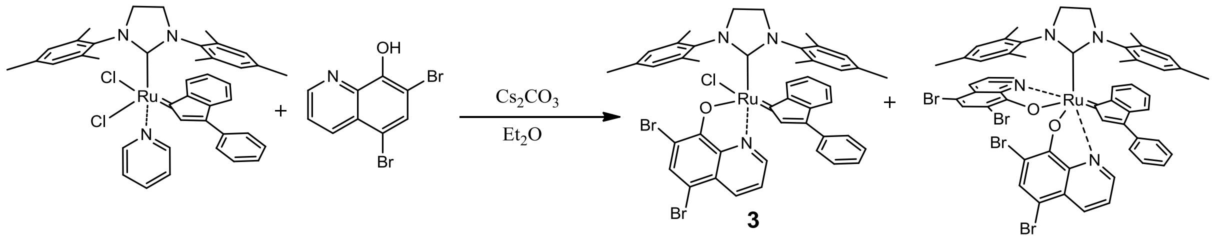

In a Schlenk flask, M31 (160 mg, 0.214 mmol, 1 equiv) was dissolved in degassed Et_2O. 5,7-Dibromo-8-hydroxquinoline (960 mg, 3.169 mmol, 15 equiv) and Cs_2CO_3 (1g, 3.077 mmol, 14 equiv) were added. The reaction mixture was stirred in a Schlenk flask under argon atmosphere for 12 hours. The reaction progress was followed by TLC (CH/EE 5:1). The insoluble residue was filtered over celite. According to a TLC (CH/EE 5:1) two products were formed. The new formed pre-catalysts were separated by column-chromatography (CH/EE 10:1), however, just the catalyst possessing one 8-quinolinolate was isolated.

3: **^{1}H-NMR** (δ, 20°C, $CDCl_3$, 300 MHz): 7.97 (d J=8.52, 1H, CH), 7.90 (d J=4.26, 1H, CH), 7.81 (s, 1H, CH^{hq}), 7.71 (d J=8.33, 1H, CH), 7.57 (s), 7.53 (bs, 3H, CH), 7.34 (bs, 3H, CH), 7.11 (d J=7.00, 1H, CH), 7.03 (q, 1H, CH), 6.49 (bs), 6.42 (s, 3H, CH + CH^{mes}), 6.27 (s, 2H, CH^{mes}), 6.18 (1H, bs, CH), 3.89 (s, 4H, CH_2), 2.30 (s, 6H, CH_3), 2.15 (bs, 6H, CH_3), 1.93 (s, 6H, CH_3).
^{13}C-NMR (δ, 20°C, $CDCl_3$, 300 MHz): Ru=C not observed, 241.9 (Ru-C), 168.8, 144.5, 144.1, 143.7, 143.0, 137.8, 137.0, 136.9, 136.6, 135.6, 135.2, 134.2, 133.4, 129.2, 129.1, 128.2, 127.8, 127.6, 127.5, 125.9, 122.6, 121.6, 117.4, 108.7, 108.3, 98.2, 3 C fehlen, 51.5 (2C, CH_2^{mes}), 21.0, 19.2 (18C, CH_3^{mes}).

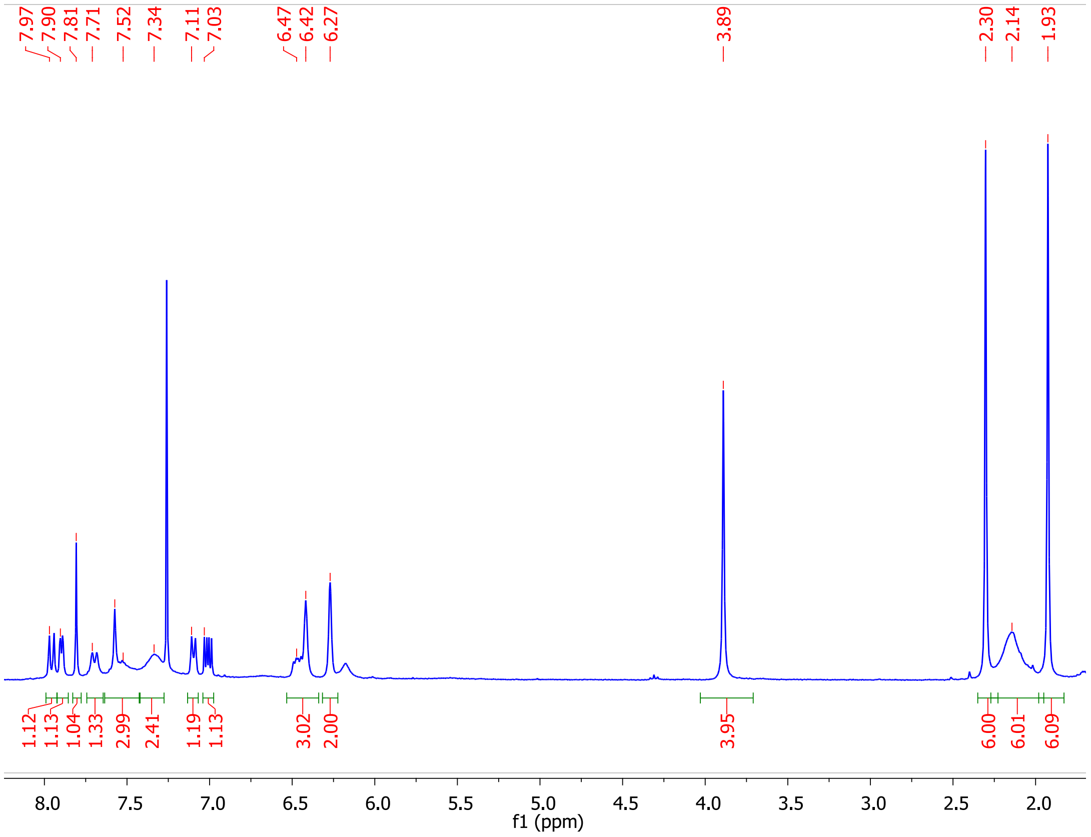
7.97
7.90
7.81
7.71
7.52
7.34
7.11
7.03
6.47
6.42
6.27
3.89
2.30
2.14
1.93
1.12
1.13
1.04
1.33
2.99
2.41
1.19
1.13
3.02
2.00
3.95
6.00
6.01
6.09
8.0 7.5 7.0 6.5 6.0 5.5 5.0 4.5 4.0 3.5 3.0 2.5 2.0
f1 (ppm)

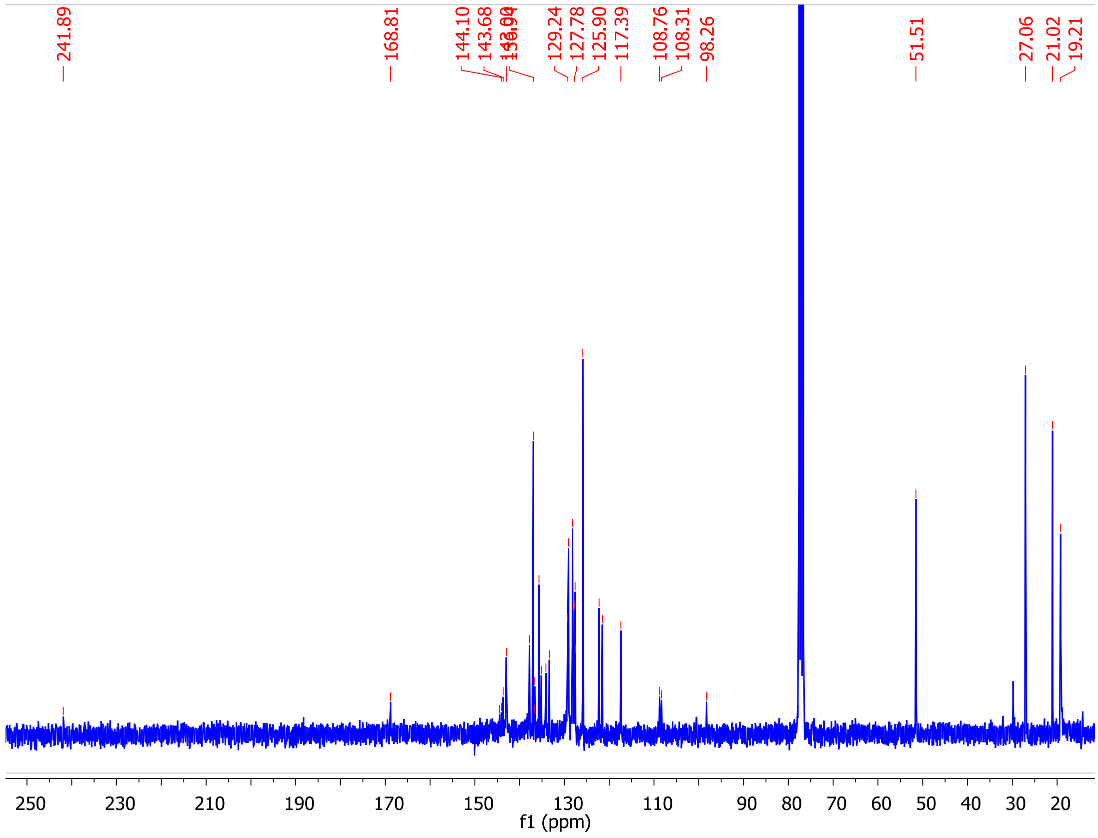
241.89
168.81
144.10
143.68
129.24
127.78
125.90
117.39
108.76
108.31
98.26
51.51
27.06
21.02
19.21
250 230 210 190 170 150 130 110 90 80 70 60 50 40 30 20
f1 (ppm)

Di(5,7-dichloro-8-quinolinolate)(isopropylbenzylidene)(1,3-bis(2,4,6-trimethylphenyl)-4,5-dihydroimidazol-2-ylidene)ruthenium (4)

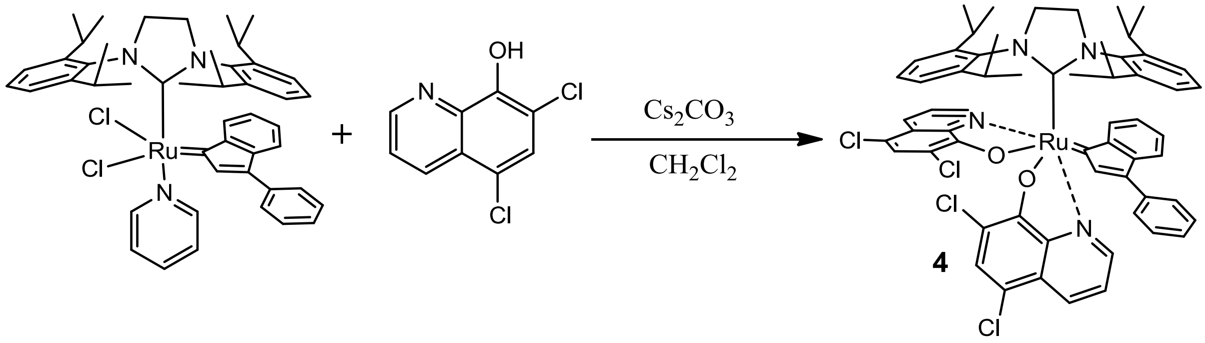

In a Schlenk flask, (Py)(H_2IPr)(Cl_2)Ru(3-phenyl-1*H*-inden-1-ylidene) (58 mg, 0,0697 mmol, 1 equiv) was dissolved in degassed CH_2Cl_2 (≈6 mL). Dichlorohydroxyquinoline (136 mg; 0,6355 mmol; 9 equiv) and Cs_2CO_3 (300 mg, 0,9230 mmol, 13 equiv) were added. The reaction mixture was stirred in a Schlenk flask under argon atmosphere overnight. The insoluble residue was filtered over celite. According to a TLC (CH/EE 5:1) two products were formed. The products were separated via column chromatography (CH/EE 5:1). The first derivative was just formed in very low amount and not identified. The second derivative was identified by NMR spectroscopy (^{1}H, COSY, ^{13}C and APT). Yield = 52%.

4: **^{1}H-NMR** (δ, 20°C, $CDCl_3$, 300 MHz): 8.03 (m, 3H, CH), 7.91 (s + d, 2H, CH), 7.67 (t, 1H, CH), 7.53 (t, 2H, CH), 7.41 (2s, 3H, CH), 7.30 (2s, 2H, CH), 7.21 (s, 1H, CH), 7.16 (m, 2H, CH), 6.79 (m, 2H, CH), 6.65 (m, 2H, CH), 6.47 (q, 1H, CH), 6.26 (m, 2H, CH), 5.9 (d, 1H J=4.51, CH^{hq}), 4.66, 4.17, 3.9, 3.76, 3.48 (8H, CH^{isop} + CH_2), 1.65, 1.34, 1.27, 1.17, 0.91, 0.61, 0.45 (d, 24H, $CH_3$9).

^{13}C-NMR (δ, 20°C, $CDCl_3$, 300 MHz)[i]: 286.2 (Ru=C), 206.6 (1C, Ru-C), 165.4 (1C, C_q), 162.0 (1C, C_q), 151.0 (1C, CH), 147.4, 146.4, 145.8, 145.6, 145.3, 145.2 (6C, C_q), 145.1 (1C, CH), 144.7 (1C, C_q), 144.0 (1C, CH), 141.2, 141.1, 139.3, 138.4, 137.0 (5C, C_q), 133.8, 132.6, 130.0, 129.8, 129.7, 128.9, 127.8, 127.7, 127.6, 126.0 (10C, CH), 125.8 (1C, C_q), 125.3 (1C, CH), 125.2 (1C, C_q), 124.8, 124.4, 124.3, 123.1, 121.5 (5C, CH), 120.4 (1C, C_q), 120.0, 117.9 (2C, CH), 117.7, 112.6, 108.4 (3C, C_q), 58.7, 55.9 (2C, CH_2), 29.7, 28.6, 28.5, 27.3, 26.9, 26.1, 25.6, 24.8, 24.6, 22.0, 21.9, 21.2 (12C, CH^{isop} + CH_3).

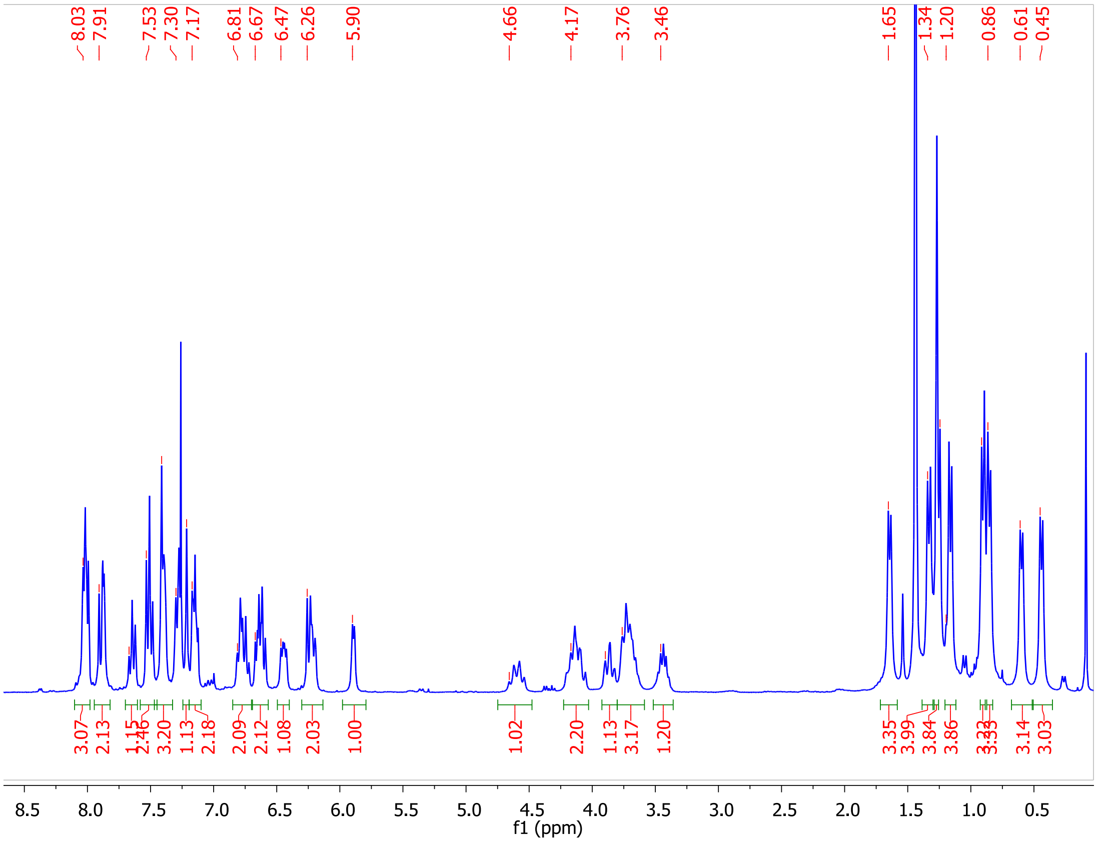
8.03
7.91
7.53
7.30
7.17
6.81
6.67
6.47
6.26
5.90
4.66
4.17
3.76
3.46
1.65
1.34
1.20
0.86
0.61
0.45
3.07
2.13
1.15
2.46
3.20
1.13
2.18
2.09
2.12
1.08
2.03
1.00
1.02
2.20
1.13
3.17
1.20
3.35
3.99
3.84
3.86
3.33
3.53
3.14
3.03
f1 (ppm)

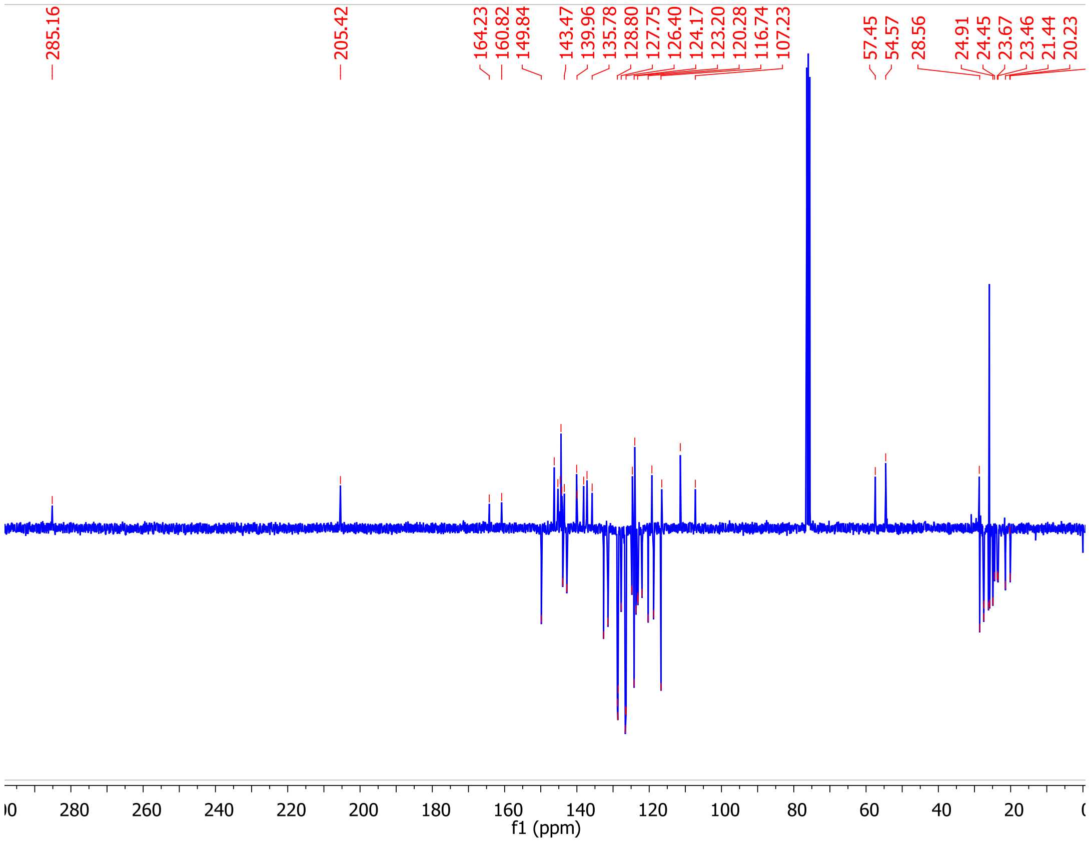
285.16
205.42
164.23
160.82
149.84
143.47
139.96
135.78
128.80
127.75
126.40
124.17
123.20
120.28
116.74
107.23
57.45
54.57
28.56
24.91
24.45
23.67
23.46
21.44
20.23
f1 (ppm)

2. Product characterization of ROMP products

a. Polymerization in a NMR tube

In an NMR tube, catalysts **1–4** (1 equiv) were dissolved in $CDCl_3$. **5** (50 equiv) dissolved in $CDCl_3$ was added. A concentration of 0.1 mol/L **5** was used. ^{1}H NMR spectra were recorded periodically and until the reaction was complete or no further conversion of the monomers could be observed to obtain conversion time plots

b. Polymerization in a Schlenk tube

Defined solutions of pre-catalyst **1–4** and **5** (300 equiv, 0.1 mol/L) were prepared. The reactions were performed in CH_2Cl_2 for room temperature and in toluene for 80 °C polymerizations. HCl (50 equiv ethereal HCl relative to ruthenium or 2 drops of HCl in aqueous solution) was added to activate the reaction. The reaction was followed by TLC (CH:EE 3:1) and after complete conversion stopped with an excess of ethyl vinyl ether. The polymer was precipitated in vigorously stirred methanol (approx. 50 mL for 100 mg polymer), and the white to yellowish precipitate was sampled and dried in vacuum. Yield: 90–20%.

^{1}H NMR (δ, 20 °C, CDCl3, 300 MHz): 5.50–5.19 (bm, 2H, C*H*=C*H*), 3.63 (bd, 6H, $CH_3^{5,5'}$), 3.24–2.97 (bd, 4H, $^{CH2,2',3,3'}$), 1.97, 1.47 (bs, 2H,CH^1).

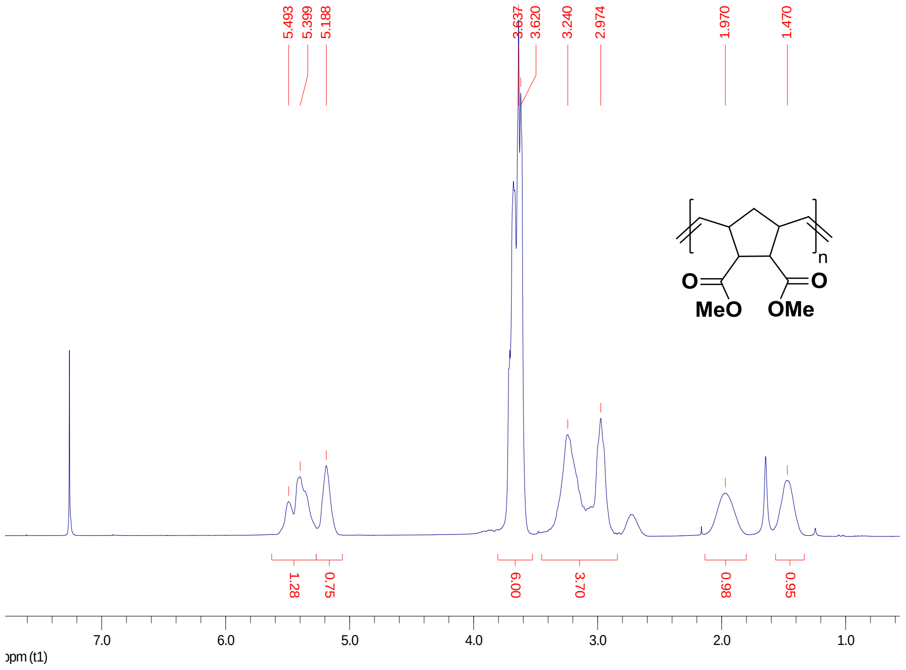

NMR spectra of 1b, 2a and 2b:

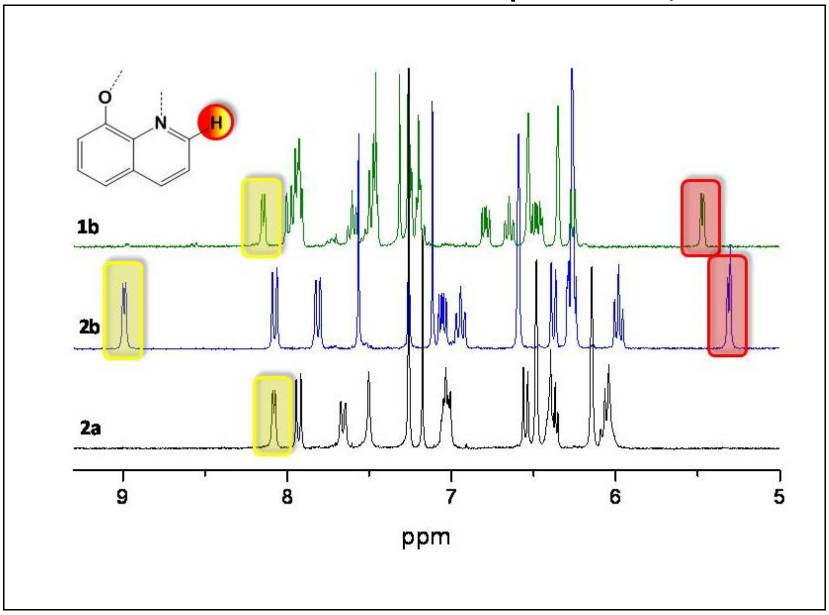

3. STA measurements

For the TGA-DTA measurements, a defined reaction solution of pre-catalyst **1**–**4** respectivly (100 ppm) and DCPD were prepared. The corresponding amount of ethereal HCl (25equiv relative to ruthenium) was added. To avoid polymerization before the measurements starts, the reaction solution was cooled immediately with liquid nitrogen after the HCl was added. A weighted portion of the frozen pre-catalyst/DCPC/HCl mixture was put promptly into the STA and measured with a temperature program of 3 °C/min. The TGA is operated with a helium flow rate of 50 mL/min used in combination with a protetctive flow of 8 mL/min.

4. Tensile strength tests

To obtain the shoulder-test-bars, the appropriate amount of pre-catalyst was mixed with 28 mL of DCPD. The mixture was shacked, heated or if necessary put into an ultrasonic bath until the catalyst was totally dissolved. Afterwards, the corresponding amount of etherical HCl was added and the mixture put immediately into a cold shoulder-test-bar form. Until complete hardening of the test bar was observable, the form was kept in an at 60 °C temperate dry-box.

5. Computational details

All calculations were performed with the Gaussian09 package Gaussian 09,[ii] Revision A.1, at the BP86 GGA level[iii] using the SDD ECP on Ru[iv] and the split-valence plus one polarization function SVP basis set on all main group atoms during geometry optimizations.[v] Furthermore

diffuse basis sets have been incorporated for O and Cl.[vi] The reported energies have been optimized via single point calculations on the BP86 geometries with triple-ζ valence plus polarization (TZVP keyword in Gaussian) using the M06 functional.[vii] Solvent effects, dichloromethane, were calculated with the PCM model,[viii] and non-electrostatic terms were also included. The geometry optimizations were performed without symmetry constraints, and the nature of the extrema was checked by analytical frequency calculations.

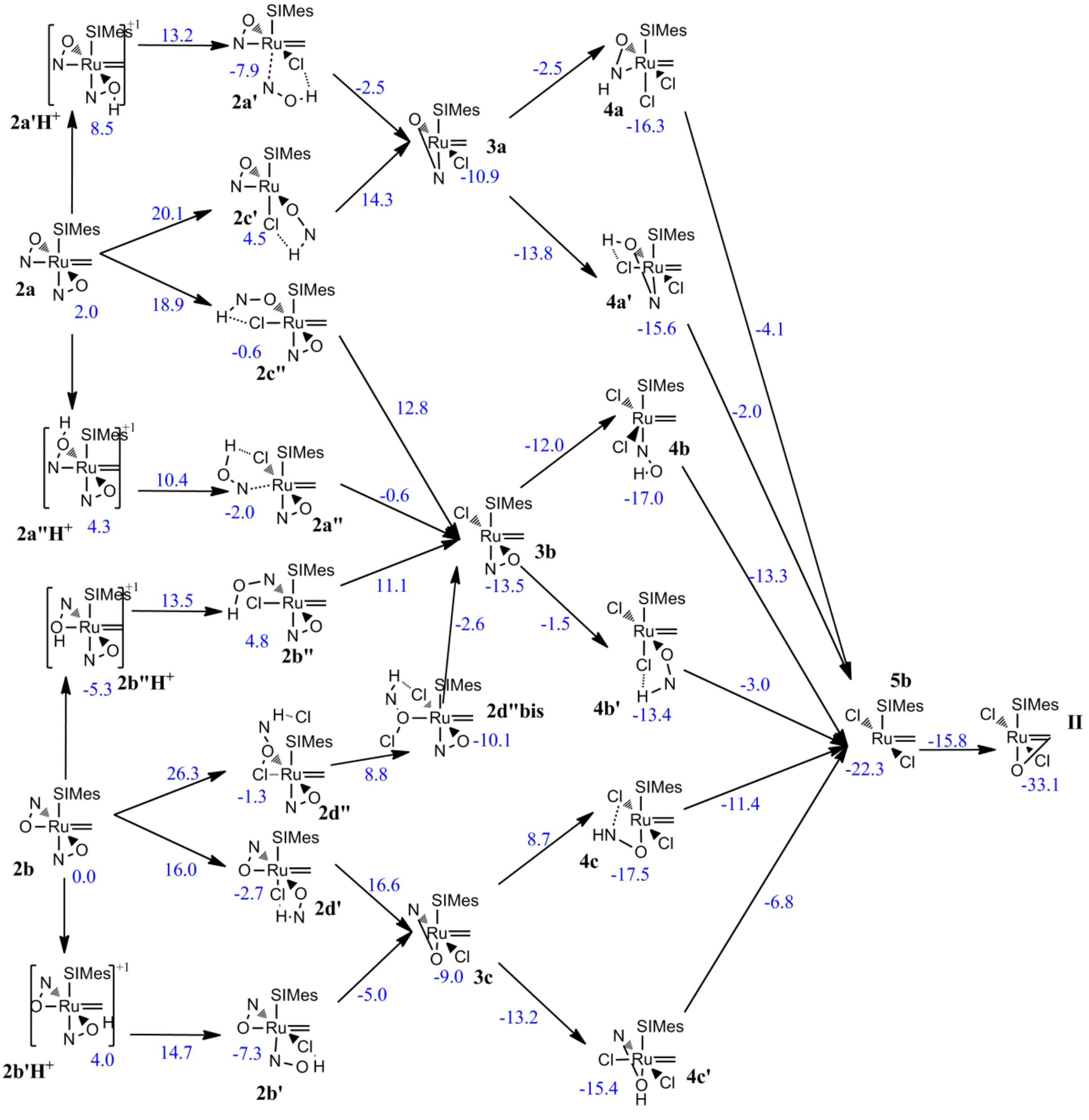

Figure S1. Reaction pathways for **2a** and **2b** (in kcal/mol). The ligands have been simplified for the sake of clarity.

Table S1. coordinate data sets and absolute energies for DFT optimized complexes (in a.u.).

2a				2a'H+				2a'H+→2a'				2a'			
-4274.4065755				-4274.8260495				-4735.1894584				-4735.2165833			
Ru	0.071128	-0.004208	-0.232504	Ru	0.081438	-0.063396	-0.245671	Ru	0.21358200	-0.03916300	-0.42980700	Ru	0.577672	-0.497936	-0.605352
Cl	1.208657	3.868057	-3.350052	Cl	1.843932	3.766328	-3.303283	Cl	0.42628600	5.00005600	-3.22166400	Cl	-3.943746	3.695461	-3.989504
Cl	1.110459	6.767804	1.263792	Cl	1.347630	6.734781	1.246757	Cl	-1.96246800	6.69461500	1.34807800	Cl	-5.311396	5.260317	1.033264
Cl	-2.239083	-2.822947	3.354055	Cl	-2.490309	-2.151110	3.538982	Cl	-1.43391400	-2.34426500	3.75069800	Cl	-0.268820	-1.769104	4.286053
Cl	-6.475584	0.488380	2.444982	Cl	-6.476339	1.219746	1.987370	Cl	-6.33973900	-0.49391400	2.25720100	Cl	-5.391540	-2.593629	2.611940
O	0.688817	1.516015	-1.503140	O	0.968982	1.491454	-1.520236	O	1.01228200	2.50578000	-1.62402200	O	-1.774413	2.214425	-2.663416
O	-0.937398	-1.250934	1.137549	O	-1.053273	-1.072708	1.138445	O	-0.52903500	-1.10642900	1.16659100	O	0.226773	-1.052233	1.396634
N	0.275264	1.616048	1.140474	N	0.303479	1.624430	1.058837	N	-0.04136900	1.86544400	0.82103900	N	-1.263909	2.088940	0.071249
N	-1.117937	-1.803246	-2.453761	N	-1.194424	-2.196257	-2.130981	N	-0.50931000	-2.51286000	-2.12338300	N	1.366568	-3.246672	-1.262662
N	1.076632	-1.920441	-2.420990	N	0.999916	-2.204125	-2.251812	N	1.66524600	-2.11585500	-2.12498800	N	3.176787	-1.983880	-1.110793
C	4.678985	-0.918926	-2.447475	C	4.438138	-0.781979	-2.693094	C	5.14916800	-0.69998200	-1.98857200	C	5.655370	0.767159	-1.625575
H	5.319292	-0.149838	-2.911638	H	4.939066	0.009931	-3.275781	H	5.76139200	0.05462500	-2.51075800	H	6.038507	1.392559	-2.449408
C	2.474964	-1.869947	-2.092393	C	2.404279	-1.965188	-2.073125	C	3.01559100	-1.82690700	-1.71045300	C	4.137405	-0.951362	-0.836287
C	3.296301	-0.885842	-2.706156	C	3.048182	-0.949994	-2.830996	C	3.81386300	-0.87906500	-2.40513200	C	4.638779	-0.164460	-1.907126
C	4.432820	-2.893415	-1.079417	C	4.554201	-2.648830	-1.168193	C	4.93161600	-2.43697500	-0.33192000	C	5.708536	0.090191	0.693372
H	4.874344	-3.687488	-0.453542	H	5.144470	-3.330084	-0.532920	H	5.37053500	-3.06830600	0.45877300	H	6.137216	0.174044	1.706162
C	3.041439	-2.907627	-1.304916	C	3.163098	-2.852731	-1.262404	C	3.59092100	-2.65211300	-0.70031900	C	4.688041	-0.854814	0.469236
C	2.200934	-4.025390	-0.727737	C	2.531649	-4.018229	-0.534113	C	2.79587200	-3.75397300	-0.04028600	C	4.194263	-1.734543	1.594742
H	2.229749	-4.927578	-1.379088	H	2.763439	-4.977025	-1.047546	H	2.40995300	-4.48571400	-0.78233800	H	4.363251	-2.812546	1.383339
H	1.140475	-3.733010	-0.603887	H	1.431420	-3.927343	-0.462603	H	1.91169600	-3.36055000	0.50224000	H	3.103783	-1.611179	1.765254
H	2.584424	-4.333600	0.265295	H	2.930758	-4.100514	0.496224	H	3.42061900	-4.31132800	0.68464900	H	4.717513	-1.497451	2.541386
C	5.268772	-1.903945	-1.628355	C	5.213215	-1.614865	-1.858983	C	5.72608600	-1.44997900	-0.94798200	C	6.201183	0.917212	-0.334638
C	2.706742	0.161545	-3.618044	C	2.295121	-0.102333	-3.834820	C	3.29857200	-0.10546100	-3.59192400	C	4.111543	-0.326873	-3.312060
H	3.497075	0.820797	-4.027055	H	2.458369	-0.468619	-4.872205	H	3.47224800	-0.67476800	-4.53316200	H	4.572146	0.413483	-3.994965
H	1.966812	0.790769	-3.080058	H	2.660032	0.948012	-3.839844	H	3.83617700	0.85783800	-3.69996200	H	3.008531	-0.205102	-3.351487
H	2.175408	-0.298679	-4.479641	H	1.198363	-0.121748	-3.672329	H	2.21171300	0.10266800	-3.52801900	H	4.338283	-1.337210	-3.718646
C	6.751547	-1.879819	-1.333857	C	6.699948	-1.393682	-1.709140	C	7.14600400	-1.19452600	-0.49621300	C	7.272058	1.950531	-0.065622
H	7.334673	-1.502397	-2.198831	H	7.164293	-1.068792	-2.662314	H	7.17315600	-0.39633900	0.27814700	H	8.020014	1.986473	-0.884625
H	7.135565	-2.886260	-1.070585	H	7.219010	-2.310589	-1.366346	H	7.78885100	-0.85561300	-1.33410500	H	7.809348	1.749310	0.882902
H	6.970295	-1.207099	-0.475474	H	6.904185	-0.598432	-0.959034	H	7.60569800	-2.10066000	-0.05153800	H	6.829044	2.967883	0.010661
C	-0.006090	-1.368433	-1.769181	C	-0.070967	-1.603207	-1.627304	C	0.46408400	-1.70814300	-1.58104300	C	1.808905	-1.978075	-0.955074
C	-0.800561	-2.577256	-3.673617	C	-0.916070	-3.236239	-3.151053	C	0.02490800	-3.49780300	-3.08931800	C	2.437251	-4.115412	-1.789378
H	-1.052538	-1.985221	-4.581674	H	-1.401971	-2.966410	-4.112223	H	-0.53554100	-3.44875600	-4.04469600	H	2.302526	-4.278642	-2.882197
H	-1.397631	-3.512015	-3.705260	H	-1.342910	-4.208716	-2.827493	H	-0.08898400	-4.52847500	-2.68259400	H	2.411210	-5.107641	-1.293818
C	0.704779	-2.822457	-3.531277	C	0.615866	-3.244017	-3.232778	C	1.48953400	-3.06912000	-3.23937000	C	3.707630	-3.314256	-1.471467
H	0.945570	-3.877441	-3.268892	H	1.061411	-4.222469	-2.954559	H	2.20528600	-3.91265900	-3.15312200	H	4.287410	-3.744540	-0.623091
H	1.277358	-2.568057	-4.447243	H	1.001303	-2.981082	-4.241308	H	1.67993500	-2.55476200	-4.20717600	H	4.395958	-3.234640	-2.337650
C	-4.448795	-2.587745	-0.954407	C	-4.602894	-2.608975	-0.646698	C	-3.43946300	-3.81961300	-0.18585100	C	-1.428625	-4.996112	0.491964
H	-4.885947	-3.316427	-0.251491	H	-5.097956	-3.217749	0.128037	H	-3.62104400	-4.43695900	0.70988300	H	-1.643308	-5.405381	1.493300
C	-3.065420	-2.645029	-1.210079	C	-3.215227	-2.763511	-0.834711	C	-2.10736100	-3.53652700	-0.54616800	C	-0.188990	-4.367668	0.273133
C	-3.325728	-0.749880	-2.775397	C	-3.337328	-1.104537	-2.665462	C	-2.94982100	-2.30541800	-2.51279500	C	-0.854674	-3.969609	-2.076049
C	-6.758243	-1.564686	-1.229698	C	-6.855097	-1.550410	-1.166754	C	-5.95624900	-3.62073100	-0.49076200	C	-3.732490	-5.765430	-0.242738
H	-7.340739	-1.061489	-2.027745	H	-7.391772	-1.206555	-2.073609	H	-6.65934100	-3.63141100	-1.34837000	H	-4.311289	-5.939637	-1.171648
H	-6.919232	-0.992494	-0.289273	H	-7.033184	-0.794250	-0.370477	H	-6.30665900	-2.83410900	0.21377100	H	-4.350362	-5.126673	0.425508
H	-7.189319	-2.574601	-1.070779	H	-7.322132	-2.496217	-0.825394	H	-6.04135300	-4.59166500	0.03893400	H	-3.608681	-6.740958	0.273062
C	-2.507356	-1.691644	-2.104244	C	-2.585876	-1.971440	-1.831349	C	-1.87019100	-2.72747700	-1.69266400	C	0.071893	-3.822898	-1.013992
C	-4.704936	-0.734673	-2.482844	C	-4.721595	-0.985355	-2.431321	C	-4.26362500	-2.61019400	-2.09971400	C	-2.081687	-4.609871	-1.802003
H	-5.344767	0.008554	-2.987976	H	-5.311005	-0.307343	-3.071043	H	-5.10237900	-2.26182200	-2.72594900	H	-2.815159	-4.714202	-2.619217
C	-5.286343	-1.634326	-1.568678	C	-5.375336	-1.719969	-1.422011	C	-4.53642200	-3.35015200	-0.93418700	C	-2.396469	-5.116541	-0.526780
C	-2.205246	-3.706040	-0.564300	C	-2.436244	-3.759180	-0.006687	C	-0.97182500	-4.11563800	0.26404700	C	0.839924	-4.289021	1.376514
H	-2.822954	-4.412274	0.023441	H	-3.108850	-4.316705	0.672635	H	-1.35365400	-4.82062700	1.02769000	H	0.491486	-4.822719	2.281732
H	-1.460261	-3.248850	0.120801	H	-1.668131	-3.249645	0.611490	H	-0.41029700	-3.31512700	0.78834200	H	1.044469	-3.234797	1.657103
H	-1.643332	-4.294346	-1.321720	H	-1.912281	-4.505884	-0.641689	H	-0.24943300	-4.66567500	-0.37521600	H	1.807456	-4.741478	1.068925
C	-2.752724	0.204232	-3.799179	C	-2.700368	-0.361849	-3.818920	C	-2.72979800	-1.59866900	-3.82845900	C	-0.547051	-3.463074	-3.465826
H	-1.708522	0.488745	-3.561189	H	-1.643767	-0.097797	-3.614256	H	-1.84192900	-0.93617500	-3.80636100	H	-0.185970	-2.412264	-3.454473
H	-3.360779	1.128179	-3.866606	H	-3.253256	0.570333	-4.048898	H	-3.61551400	-0.99179600	-4.10408100	H	-1.443876	-3.517528	-4.113997
H	-2.750107	-0.249318	-4.815924	H	-2.713243	-0.974543	-4.748085	H	-2.58309100	-2.33476100	-4.65166100	H	0.244253	-4.071785	-3.956679
N	-2.008652	0.806895	-0.242039	N	-1.913921	0.887384	-0.527435	N	-2.01973900	0.29496100	-0.58458100	N	-1.577535	-1.051016	-0.601111
C	-2.476402	1.871214	-0.903841	C	-2.300805	1.865718	-1.352659	C	-2.73278600	1.00925900	-1.46404000	C	-2.449707	-0.983656	-1.614750
H	-1.811576	2.303295	-1.667824	H	-1.588670	2.160234	-2.139868	H	-2.17600700	1.37608000	-2.34202600	H	-2.031677	-0.693827	-2.592475
C	-3.757795	2.417568	-0.643505	C	-3.564725	2.498247	-1.244336	C	-4.12115300	1.24590100	-1.30041900	C	-3.826777	-1.261788	-1.440100
H	-4.092210	3.301486	-1.207140	H	-3.832255	3.299550	-1.948891	H	-4.65365400	1.84453300	-2.05472200	H	-4.506434	-1.177935	-2.301039
C	-2.150671	-0.875888	1.437413	C	-2.246617	-0.552510	1.342905	C	-1.80946100	-0.95947800	1.42286500	C	-1.004755	-1.388613	1.682165
C	-4.103074	0.694320	1.040534	C	-4.064061	1.063137	0.657306	C	-4.06240600	-0.04547800	0.75528300	C	-3.393664	-1.727765	0.909889
C	-2.774420	0.226334	0.735133	C	-2.758272	0.480481	0.477044	C	-2.65340500	-0.22352500	0.51735000	C	-2.017450	-1.392249	0.652102
C	-4.573298	1.827909	0.314715	C	-4.444435	2.096527	-0.246420	C	-4.78832900	0.72056400	-0.20184500	C	-4.297154	-1.640718	-0.187771
H	-5.576756	2.221686	0.536179	H	-5.432779	2.566559	-0.131342	H	-5.86578200	0.88332800	-0.04844700	H	-5.359909	-1.872503	-0.019886
C	-2.936636	-1.494104	2.446915	C	-3.083545	-0.952129	2.413324	C	-2.42471400	-1.48576100	2.58486800	C	-1.438459	-1.759841	2.980886
C	-4.827560	-0.002426	2.050929	C	-4.862789	0.575765	1.734225	C	-4.61367200	-0.63999700	1.92930500	C	-3.737966	-2.113392	2.239591
C	-4.250251	-1.070533	2.740047	C	-4.372470	-0.403598	2.598114	C	-3.80746700	-1.33649900	2.82865900	C	-2.777255	-2.123431	3.251001
H	-4.817658	-1.589420	3.525623	H	-4.988489	-0.756943	3.437202	H	-4.24319600	-1.77518600	3.73734300	H	-3.057029	-2.415080	4.273267
C	0.571526	2.797438	0.504794	C	0.705666	2.792773	0.449797	C	-0.24320900	3.12992800	0.30593800	C	-2.314138	2.842339	-0.375275
C	0.504114	3.989020	2.637368	C	0.500680	4.017653	2.553394	C	-1.14093200	3.89998700	2.46076300	C	-2.867678	3.514280	1.914423
H	0.589812	4.920075	3.217384	H	0.566671	4.952206	3.129424	H	-1.60569400	4.68372900	3.07723100	H	-3.503257	4.066609	2.623006
C	0.224395	2.774254	3.251508	C	0.130457	2.819743	3.151926	C	-0.79355100	2.66579100	2.98688200	C	-1.789766	2.754371	2.341472
H	0.082232	2.710367	4.340675	H	-0.106771	2.772107	4.224696	H	-0.95695100	2.42688100	4.04789500	H	-1.533647	2.675464	3.408572
C	0.756096	2.694545	-0.923331	C	1.044854	2.732978	-0.931994	C	0.21951300	3.40496700	-1.02784500	C	-2.548883	2.878214	-1.798458
C	1.120321	5.141419	-0.928074	C	1.530902	5.129017	-0.926885	C	-0.80414800	5.64710800	-0.87273900	C	-4.475332	4.365651	-1.396192
H	1.328277	6.054254	-1.503846	H	1.845771	6.026916	-1.476127	H	-1.03015600	6.60848300	-1.35393400	H	-5.307396	4.947567	-1.815785
C	0.964424	5.210563	0.458089	C	1.222410	5.199924	0.429531	C	-1.15906000	5.41711300	0.44971500	C	-4.248583	4.342867	-0.026257
C	0.112245	1.596850	2.469102	C	0.041731	1.639912	2.372932	C	-0.26855500	1.67050900	2.12557500	C	-1.012566	2.055396	1.377722
H	-0.114830	0.616013	2.915390	H	-0.259337	0.675983	2.812195	H	-0.05944000	0.65738500	2.50195900	H	-0.156260	1.438434	1.700599
C	1.016922	3.907919	-1.608776	C	1.445703	3.887305	-1.603031	C	-0.10210200	4.65011900	-1.59571600	C	-3.630511	3.639257	-2.271784
C	0.685119	4.039095	1.222681	C	0.809477	4.037108	1.162528	C	-0.87012300	4.17007500	1.09036900	C	-3.168051	3.587193	0.525206
O	3.001945	-1.908981	2.613830	O	2.797573	-2.140983	2.771247	O	3.18597900	-0.86258900	2.83238700	O	2.774399	2.188363	2.236514
C	1.678289	-0.762548	0.456676	C	1.591135	-0.913255	0.585198	C	1.88323600	-0.31924900	0.44994300	C	1.917782	0.603174	0.139574
H	1.587890	-1.833746	0.732954	H	1.380331	-1.947016	0.931701	H	1.93287400	-1.30420600	0.95924300	H	2.321812	0.245057	1.109354
C	3.444672	1.023949	0.463998	C	3.478234	0.745323	0.618608	C	3.34538100	1.72770100	0.24248300	C	2.126546	2.648257	-1.343884
H	2.930620	1.477543	-0.394172	H	3.030588	1.219039	-0.264332	H	2.80620000	1.97581500	-0.68240800	H	1.738674	2.047316	-2.179772
C	5.283067	0.963070	2.037298	C	5.239341	0.598699	2.266774	C	5.05070700	2.20463600	1.89468100	C	2.983970	4.732356	-0.456417

H	6.210055	1.396129	2.447215	H	6.172034	0.983521	2.709498	H	5.86974000	2.83789300	2.27331600	H	3.272832	5.787153	-0.593717
C	3.584402	-0.790759	2.102863	C	3.444708	-1.061079	2.280501	C	3.63497900	0.23191500	2.16190000	C	2.734037	2.805136	1.023167
C	2.886156	-0.163910	1.006843	C	2.823749	-0.401870	1.149997	C	2.94034900	0.55714700	0.93717100	C	2.241780	2.011036	-0.079463
C	4.628327	1.584911	0.959668	C	4.668026	1.244848	1.152214	C	4.38565700	2.54326500	0.70259500	C	2.495786	3.983145	-1.541890
H	5.040035	2.501076	0.509341	H	5.154710	2.127222	0.710048	H	4.68046300	3.43949300	0.13514900	H	2.400181	4.441566	-2.538222
C	4.771854	-0.217436	2.606433	C	4.642256	-0.541537	2.825260	C	4.68345800	1.05935700	2.62211900	C	3.104445	4.152145	0.817712
H	5.307879	-0.678908	3.447556	H	5.119922	-1.023588	3.688774	H	5.21743100	0.82449300	3.55337200	H	3.480927	4.763620	1.649403
C	3.565626	-2.605334	3.749611	C	3.267529	-2.847000	3.958841	C	3.75961000	-1.26637300	4.09836000	C	3.203755	2.887190	3.428627
H	3.955639	-1.848293	4.468758	H	3.660012	-2.089319	4.673927	H	3.98001600	-0.34921900	4.69216300	H	2.801206	3.925806	3.391820
C	2.405354	-3.354173	4.403109	C	2.035384	-3.512263	4.565482	C	2.67617400	-2.06947400	4.81592900	C	2.561504	2.151529	4.603647
H	1.597886	-2.654529	4.695438	H	1.254497	-2.763024	4.801799	H	1.75714900	-1.46574300	4.94853400	H	1.457947	2.139857	4.508327
H	2.755295	-3.890616	5.308508	H	2.310118	-4.039444	5.501136	H	3.03543900	-2.39182100	5.81441900	H	2.826856	2.647918	5.559177
H	1.975369	-4.096922	3.700174	H	1.604038	-4.254266	3.862834	H	2.40849600	-2.97326800	4.23117100	H	2.912260	1.100089	4.645553
C	4.701158	-3.534161	3.307469	C	4.368822	-3.842673	3.589217	C	5.04725800	-2.06678300	3.87957600	C	4.732429	2.925697	3.518510
H	4.312885	-4.317092	2.623500	H	3.972410	-4.625936	2.910467	H	4.82950400	-2.99300700	3.30898000	H	5.137532	1.895889	3.595440
H	5.153847	-4.037953	4.186471	H	4.750958	-4.344916	4.501331	H	5.49306200	-2.35603700	4.85370200	H	5.051831	3.492499	4.417504
H	5.502258	-2.980271	2.778642	H	5.226263	-3.349451	3.089127	H	5.80322100	-1.48474200	3.31578900	H	5.184999	3.408260	2.629529
				H	1.365662	1.468108	-2.424674	H	0.60794500	2.04651400	-2.46787100	H	-1.052970	1.720851	-2.192598
								Cl	-0.09017500	0.68428500	-3.40330000	Cl	0.511732	0.083662	-2.930073

2a'→3a				3a				3a→4a				4a			
-4735.210334				-3339.0395572				-3799.823937				-3799.852003			
Ru	1.480626	-0.260278	-0.602541	Ru	0.386916	-0.556057	0.584118	Ru	-0.883037	0.096629	0.724328	Ru	-1.204442	-0.008928	0.806840
Cl	-6.193867	1.535036	-4.015983	Cl	1.563840	2.379383	-3.452057	Cl	3.881238	-5.191966	-0.660059	Cl	4.888744	-4.194428	-1.583626
Cl	-8.770627	-1.409375	-0.224597	Cl	3.839022	5.573655	0.333675	Cl	0.355914	-2.255390	-3.571964	Cl	-0.011495	-2.561629	-3.222070
Cl	2.872533	-2.071161	3.990162	O	0.939742	0.576747	-1.105731	N	-1.252989	2.632821	-0.894258	N	-1.192857	1.534884	-1.889353
Cl	-1.606774	-5.128556	3.429397	N	1.056595	-3.062789	-0.813796	N	-3.075631	1.388251	-0.754457	N	-3.018538	0.367690	-1.431610
O	-3.892486	1.472053	-2.054843	N	-1.112310	-2.724570	-1.033190	N	0.253184	-2.205672	1.568474	N	1.415707	-2.278374	1.728958
O	2.133528	-1.046745	1.240685	C	-4.612214	-2.186368	0.145963	O	-0.509651	-1.179371	-0.923410	O	-0.369948	-1.618714	-0.403638
N	-3.854844	0.286553	0.323199	H	-5.275970	-2.534120	0.955270	O	3.471741	0.418469	-0.395932	O	3.252884	1.103707	0.676065
N	4.172722	-0.398726	-1.767288	C	-2.441714	-2.196761	-0.931851	C	-1.752488	1.453075	-0.385673	C	-1.800807	0.763467	-0.926630
N	3.881141	1.657076	-1.021963	C	-3.288232	-2.660858	0.109092	C	-2.270689	3.436157	-1.603004	C	-2.055480	1.726974	-3.079617
C	2.283314	5.007520	-0.649651	C	-4.241419	-0.848970	-1.835221	H	-2.423132	4.405754	-1.079928	H	-2.425844	2.777381	-3.110888
H	1.793089	5.765594	-1.283234	H	-4.613644	-0.145404	-2.598726	H	-1.933355	3.668913	-2.635934	H	-1.485707	1.542816	-4.012935
C	3.345048	2.841869	-0.417792	C	-2.906220	-1.293219	-1.920404	C	-3.515288	2.538217	-1.567081	C	-3.181494	0.707400	-2.856614
C	2.727618	3.813980	-1.247124	C	-1.995192	-0.809511	-3.025355	H	-3.829768	2.193354	-2.576714	H	-3.052084	-0.202923	-3.484551
C	3.063444	4.269573	1.518351	H	-1.644590	-1.645638	-3.668767	H	-4.392571	3.031618	-1.097295	H	-4.192173	1.119862	-3.055314
H	3.200162	4.448808	2.597937	H	-1.086709	-0.313800	-2.621490	C	0.051101	3.227262	-0.794199	C	-0.079296	2.437800	-1.763811
C	3.524164	3.054242	0.971798	H	-2.518836	-0.086736	-3.681005	C	0.344145	4.106771	0.280205	C	-0.239002	3.660828	-1.050262
C	4.176647	2.007187	1.845085	C	-5.109058	-1.279607	-0.812950	C	1.579963	4.786104	0.262835	C	0.814211	4.593401	-1.086310
H	5.192916	1.740060	1.483455	C	-2.774272	-3.617975	1.158054	H	1.816638	5.466991	1.097634	H	0.690646	5.543145	-0.538732
H	3.588755	1.064359	1.863753	H	-3.565247	-3.860493	1.894444	C	2.504064	4.640065	-0.788736	C	1.997491	4.369417	-1.820033
H	4.279480	2.368663	2.886967	H	-1.905918	-3.189152	1.704888	C	2.170296	3.771719	-1.849324	C	2.119231	3.155029	-2.518312
C	2.443216	5.257317	0.729116	H	-2.428906	-4.575971	0.711819	H	2.871229	3.651807	-2.693045	H	3.031399	2.958789	-3.107699
C	2.539429	3.563795	-2.724741	C	-6.531021	-0.771443	-0.724250	C	0.956344	3.060897	-1.877503	C	1.097969	2.180880	-2.510860
H	3.512444	3.462201	-3.253566	H	-7.246523	-1.594088	-0.515406	C	-0.636169	4.326407	1.405150	C	-1.505584	3.979526	-0.297762
H	1.990119	4.399092	-3.201306	H	-6.848356	-0.270727	-1.660869	H	-0.229117	5.035953	2.151853	H	-1.498057	5.027671	0.060105
H	1.976535	2.622635	-2.907655	H	-6.636330	-0.036151	0.103409	H	-1.597326	4.746387	1.036072	H	-2.406298	3.844640	-0.933473
C	1.952829	6.554332	1.333920	C	0.030471	-2.212570	-0.487439	H	-0.885116	3.374019	1.921273	H	-1.645878	3.311085	0.580246
H	2.386178	7.434741	0.813162	C	0.615619	-4.240805	-1.586814	C	3.820690	5.384701	-0.783048	C	3.084391	5.421028	-1.862352
H	2.216355	6.632620	2.407513	H	0.783712	-5.175972	-1.008236	H	3.831543	6.190365	-0.022240	H	2.698278	6.381951	-2.265386
H	0.848334	6.642232	1.246586	H	1.193438	-4.325604	-2.531706	H	4.667581	4.701304	-0.554412	H	3.479655	5.638518	-0.846989
C	3.285172	0.434585	-1.133741	C	-0.880189	-3.952295	-1.823694	H	4.035037	5.842113	-1.771673	H	3.935762	5.107699	-2.499161
C	5.404190	0.290908	-2.200832	H	-1.118012	-3.773694	-2.895899	C	0.634706	2.127356	-3.021984	C	1.257272	0.915243	-3.319061
H	5.439008	0.371097	-3.310232	H	-1.539490	-4.772915	-1.469879	H	-0.312062	2.403158	-3.533678	H	0.922655	1.060683	-4.371062
H	6.303781	-0.272199	-1.874933	C	4.611955	-1.944391	-1.177870	H	1.440115	2.140682	-3.782146	H	2.319816	0.602561	-3.366781
C	5.274372	1.666039	-1.519240	H	5.225680	-1.369124	-1.891554	H	0.505092	1.078727	-2.676041	H	0.663335	0.079988	-2.902669
H	5.985463	1.794642	-0.671823	C	3.242534	-2.117807	-1.457834	C	-4.041080	0.337168	-0.544109	C	-4.073998	-0.307664	-0.713019
H	5.434298	2.512438	-2.218755	C	3.045040	-3.479885	0.602375	C	-4.963743	0.451572	0.528033	C	-5.076560	0.488450	-0.090663
C	4.208415	-4.084750	-1.122294	C	6.682161	-2.255286	0.263432	C	-5.957277	-0.539032	0.654035	C	-6.121423	-0.162616	0.590564
H	4.493313	-4.775903	-0.311214	H	7.107307	-3.050142	0.909017	H	-6.668487	-0.465535	1.494033	H	-6.891346	0.453162	1.085252
C	4.375788	-2.700802	-0.916850	H	6.825591	-1.288715	0.795263	C	-6.069184	-1.609325	-0.253793	C	-6.209414	-1.566722	0.661821
C	3.560384	-2.310996	-3.224074	H	7.284620	-2.204435	-0.666687	C	-5.164957	-1.661017	-1.332087	C	-5.237119	-2.323165	-0.017413
C	3.496225	-6.094456	-2.507395	C	2.453211	-2.851094	-0.527342	H	-5.247281	-2.477643	-2.069214	H	-5.305142	-3.424042	-0.007436
H	3.644874	-6.409506	-3.560412	C	4.421092	-3.272173	0.831334	C	-4.151609	-0.699657	-1.506800	C	-4.175484	-1.724980	-0.727349
H	2.458792	-6.381049	-2.225514	H	4.884159	-3.748271	1.712069	C	-4.892453	1.587035	1.520078	C	-5.033602	1.995892	-0.140166
H	4.182186	-6.686009	-1.867738	C	5.218374	-2.495800	-0.031189	H	-5.742125	1.543608	2.229502	H	-5.995282	2.429424	0.197918
C	3.995648	-1.815016	-1.963753	C	2.643995	-1.548670	-2.721323	H	-3.947653	1.547226	2.104367	H	-4.228502	2.370069	0.528886
C	3.412853	-3.705020	-3.371766	H	3.435378	-1.160168	-3.391678	H	-4.921979	2.580801	1.022951	H	-4.825003	2.378784	-1.161128
H	3.061961	-4.095065	-4.342096	H	1.948525	-0.714559	-2.486554	C	-7.114160	-2.685721	-0.063244	C	-7.306710	-2.237570	1.456777
C	3.710317	-4.607730	-2.331851	H	2.066532	-2.311617	-3.285240	H	-7.447191	-3.111771	-1.031764	H	-7.533069	-3.252138	1.070629
C	4.960229	-2.187659	0.376694	C	2.245519	-4.356424	1.534456	H	-6.706892	-3.526976	0.540216	H	-7.006410	-2.351476	2.521842
H	5.411155	-3.014128	0.960146	H	1.474315	-3.760030	2.070305	H	-8.006595	-2.302486	0.472171	H	-8.243798	-1.644075	1.445162
H	4.174374	-1.711926	1.001503	H	2.904114	-4.832244	2.287339	C	-3.206442	-0.790622	-2.679763	C	-3.222481	-2.585540	-1.518429
H	5.746518	-1.424204	0.200036	H	1.715823	-5.167250	0.990439	H	-2.172806	-1.011548	-2.339367	H	-2.169548	-2.266687	-1.394895
C	3.286709	-1.389570	-4.387465	N	1.465862	1.069846	1.491342	H	-3.520131	-1.592066	-3.377023	H	-3.302256	-3.646612	-1.209758
H	2.541173	-0.612112	-4.114581	C	1.739959	1.241607	2.789539	H	-3.162974	0.158128	-3.257279	H	-3.461280	-2.545413	-2.604838
H	2.893166	-1.956646	-5.253680	H	1.378576	0.443513	3.460197	C	0.868159	0.722834	0.487855	C	0.508843	0.715411	0.859607
H	4.211420	-0.872137	-4.724679	C	2.446934	2.378601	3.253949	H	1.216633	0.884661	-0.551282	H	1.126917	0.606932	-0.058598
N	-0.048625	-1.709091	-0.193870	H	2.650221	2.483201	4.330110	C	1.978123	0.723736	1.437063	C	1.267349	1.211640	2.003157
C	-1.080270	-2.046374	-0.977072	C	1.550084	1.693930	-0.810018	C	3.332874	0.600929	0.943595	C	2.697548	1.398025	1.884495
H	-1.150683	-1.496476	-1.930943	C	2.594413	3.185723	0.957866	C	4.418773	0.630114	1.845668	C	3.448584	1.828249	3.000409
C	-2.011422	-3.041053	-0.588416	C	1.879168	1.998601	0.565063	H	5.450630	0.535368	1.481122	H	4.534527	1.972812	2.920595
H	-2.843840	-3.290366	-1.262816	C	2.866172	3.346226	2.347661	C	4.192958	0.774972	3.224955	C	2.815674	2.078982	4.230003
C	1.309404	-1.919428	1.757187	H	3.410358	4.243788	2.679304	H	5.056933	0.795907	3.909104	H	3.423313	2.417933	5.085053
C	-0.779229	-3.336384	1.495154	C	1.950854	2.662232	-1.767739	C	2.885237	0.888260	3.728409	C	1.429388	1.896647	4.370805
C	0.137133	-2.332920	1.018198	C	2.968161	4.099439	-0.072250	H	2.711400	1.006938	4.809179	H	0.934811	2.088808	5.335356
C	-1.868611	-3.675689	0.640363	C	2.645312	3.837978	-1.405096	C	1.801605	0.850777	2.842960	C	0.677078	1.459586	3.274673
H	-2.582355	-4.444513	0.973556	H	2.932521	4.552303	-2.189611	H	0.769521	0.947851	3.215475	H	-0.407315	1.302013	3.367563
C	1.488425	-2.536526	3.023670	O	-2.940938	2.373572	-0.261192	C	4.773607	0.216491	-1.000039	C	4.677047	1.234522	0.452496
C	-0.518879	-3.908728	2.775776	C	-1.250358	0.317948	0.400989	H	5.375237	-0.432741	-0.323046	H	5.211219	0.833591	1.345733
C	0.591910	-3.508371	3.521187	H	-1.333661	0.847195	-0.572801	C	4.521314	-0.536137	-2.304520	C	4.997672	0.351091	-0.751520
H	0.780549	-3.953572	4.508483	C	-2.609441	0.019956	2.536304	H	3.884246	0.064422	-2.985588	H	4.448906	0.708216	-1.646918
C	-5.013464	0.180839	-0.384034	H	-1.914140	-0.775585	2.845266	H	5.482015	-0.744851	-2.817692	H	6.083971	0.381987	-0.972330
C	-6.059291	-1.128506	1.381717	C	-4.545752	1.419343	2.941375	H	4.009436	-1.499118	-2.110368	H	4.708637	-0.701930	-0.565212
H	-6.920724	-1.679232	1.789728	H	-5.398740	1.717903	3.572613	C	5.489600	1.553638	-1.212017	C	5.064836	2.698566	0.224830
C	-4.869726	-1.011821	2.091416	C	-3.239821	1.732818	0.899587	H	5.629821	2.103210	-0.259754	H	4.788235	3.339052	1.085470
H	-4.756054	-1.471992	3.084693	C	-2.353648	0.657471	1.292729	H	6.489997	1.387337	-1.662136	H	6.159909	2.786781	0.068571
C	-5.002965	0.832652	-1.668931	C	-3.685977	0.384484	3.351653	H	4.900494	2.197024	-1.896617	H	4.547353	3.090873	-0.673359
C	-7.313256	0.065998	-1.992900	H	-3.854376	-0.134272	4.308044	C	0.717038	-2.520934	2.784678	C	2.232133	-2.485041	2.769072
H	-8.202614	0.031953	-2.637268	C	-4.327914	2.090182	1.727077	H	0.124440	-2.151091	3.636936	H	1.815717	-2.239167	3.757301
C	-7.323515	-0.562287	-0.751726	H	-5.011292	2.898672	1.433034	C	1.868057	-3.324442	2.947474	C	3.526530	-2.999303	2.558146
C	-3.784506	-0.290651	1.517429	C	-3.642989	3.569381	-0.677752	H	2.240748	-3.547391	3.957487	H	4.199550	-3.155240	3.412920
H	-2.827742	-0.191541	2.059250	H	-3.917935	4.150740	0.232204	C	2.478447	-3.858520	1.817924	C	3.910093	-3.328481	1.261520
C	-6.161404	0.760932	-2.452269	C	-2.642174	4.381643	-1.498504	H	3.346245	-4.530101	1.905756	H	4.900587	-3.763773	1.058720
C	-6.172247	-0.526175	0.095900	H	-1.749972	4.634831	-0.892526	C	1.991673	-3.551327	0.513233	C	3.028596	-3.130666	0.158157
O	-0.106872	2.430189	2.676203	H	-3.108038	5.324887	-1.849734	C	0.878632	-2.634198	0.421037	C	1.734534	-2.532735	0.425217
C	0.855732	1.147855	0.449260	H	-2.304202	3.806115	-2.384528	C	0.373710	-2.129952	-0.845551	C	0.746351	-2.227950	-0.608882
H	1.197637	1.055632	1.502781	C	-4.905629	3.205170	-1.463861	C	0.938621	-2.748757	-1.999918	C	1.125500	-2.727100	-1.903050
C	-0.651975	2.657893	-0.947513	H	-4.636282	2.654887	-2.388723	C	1.983843	-3.692647	-1.928750	C	2.368226	-3.325567	-2.182023
H	-0.319844	2.123337	-1.850830	H	-5.458228	4.122544	-1.754605	H	2.391625	-4.108260	-2.861271	H	2.587507	-3.642066	-3.211645
C	-2.001402	4.353942	0.136444	H	-5.588074	2.565226	-0.869834	C	2.525099	-4.073547	-0.699168	C	3.325066	-3.502645	-1.180597
H	-2.730822	5.178534	0.082870	Cl	0.208318	-1.760527	2.665810	Cl	-1.556777	1.118480	2.752963	Cl	-2.439705	1.521862	2.107639

C -0.577253 2.900626 1.489801		H -1.072593 -2.107787 1.522096	H 0.410228 -1.956055 1.977650
C -0.111182 2.229017 0.294690		Cl -2.586341 -2.011655 1.424649	Cl -1.251963 -1.520158 2.757961
C -1.581876 3.699665 -1.036656			
H -1.984200 3.998156 -2.016437			
C -1.505164 3.961418 1.389285			
H -1.855061 4.482806 2.290836			
C -0.581993 2.941253 3.944524			
H -1.666052 3.179930 3.845154			
C -0.418011 1.798569 4.946076			
H -0.997834 0.909392 4.628539			
H -0.775121 2.110904 5.948497			
H 0.647403 1.501294 5.030037			
C 0.192030 4.200085 4.345352			
H 1.267090 3.962122 4.479274			
H -0.196590 4.603796 5.303219			
H 0.108782 4.994683 3.577487			
H -3.258992 1.360730 -1.291674			
Cl 0.613412 0.198561 -2.804920			

4a→5b			
**-3799.8259731**			
Ru	1.492630	0.679710	0.869864
Cl	-7.826643	0.783893	-0.534500
Cl	-3.423534	-1.031391	-3.162144
N	2.599277	-0.272532	-1.821351
N	2.616565	1.918802	-1.491643
N	-3.044013	1.467293	1.313751
O	-1.890213	0.198582	-0.796619
O	-0.895823	-3.131991	0.996106
C	2.277165	0.713210	-0.926759
C	3.246317	0.262889	-3.039599
H	4.309328	-0.063577	-3.077016
H	2.746251	-0.124566	-3.951895
C	3.103923	1.787999	-2.876843
H	2.368580	2.227740	-3.588052
H	4.064355	2.327946	-3.013546
C	2.500872	-1.699169	-1.674541
C	3.574706	-2.422155	-1.089092
C	3.482031	-3.825740	-1.046915
H	4.308311	-4.394188	-0.587498
C	2.379056	-4.521310	-1.583485
C	1.348644	-3.771428	-2.180756
H	0.488977	-4.296474	-2.629921
C	1.383668	-2.362713	-2.242792
C	4.787981	-1.717119	-0.532091
H	5.524913	-2.447927	-0.145522
H	5.300509	-1.104452	-1.305407
H	4.516503	-1.023955	0.292723
C	2.314039	-6.030814	-1.511097
H	1.509935	-6.438697	-2.155930
H	3.272957	-6.495230	-1.822503
H	2.114764	-6.372569	-0.471871
C	0.256516	-1.594984	-2.890188
H	0.626969	-0.852717	-3.628288
H	-0.430733	-2.280883	-3.421967
H	-0.355319	-1.025671	-2.154295
C	2.285878	3.209746	-0.945166
C	3.312133	3.988325	-0.344681
C	2.974422	5.262959	0.148213
H	3.762947	5.868815	0.625696
C	1.668345	5.783117	0.049076
C	0.687522	4.999498	-0.588483
H	-0.334545	5.399280	-0.699304
C	0.967953	3.717861	-1.101543
C	4.725124	3.469894	-0.228732
H	5.381872	4.218483	0.256096
H	4.751402	2.537365	0.374747
H	5.164731	3.231955	-1.221499
C	1.331442	7.138504	0.629010
H	1.161703	7.067815	1.726046
H	2.155973	7.865348	0.476601
H	0.410387	7.561088	0.179539
C	-0.109183	2.920492	-1.796799
H	-0.444112	2.041888	-1.201235
H	-1.001583	3.548598	-1.986951
H	0.232659	2.520833	-2.774449
C	0.943708	-1.080575	0.921970
H	0.232018	-1.300167	0.089892
C	1.028886	-2.107939	1.949199
C	0.071583	-3.189705	1.946821
C	0.162360	-4.209910	2.919918
H	-0.555145	-5.041731	2.922832
C	1.168169	-4.169061	3.899548
H	1.216580	-4.974331	4.650524
C	2.099417	-3.115233	3.928419
H	2.882963	-3.083690	4.701014
C	2.020033	-2.101230	2.968215
H	2.737562	-1.266768	2.974320
C	-1.962314	-4.110882	0.938931
H	-2.243097	-4.383181	1.982515
C	-3.145353	-3.408448	0.276956
H	-2.874657	-3.046851	-0.735648
H	-4.003260	-4.105376	0.185881
H	-3.467015	-2.534209	0.876302
C	-1.506798	-5.361206	0.181330
H	-0.616855	-5.826036	0.650914
H	-2.319221	-6.116834	0.161565
H	-1.246229	-5.100678	-0.864531
C	-3.483418	2.108455	2.405381
H	-2.706757	2.448679	3.105969
C	-4.870288	2.300935	2.577006
H	-5.237174	2.824797	3.470879
C	-5.750027	1.823672	1.607255
H	-6.836861	1.962840	1.716707
C	-5.272646	1.143664	0.448307
C	-3.841596	0.971944	0.326060
C	-3.149028	0.299728	-0.784807
C	-4.079978	-0.191203	-1.773789
C	-5.475376	-0.033941	-1.680167
H	-6.107767	-0.438844	-2.483738
C	-6.077572	0.618768	-0.595950
Cl	3.592796	0.751986	1.852655
H	-2.004304	1.340414	1.193189

5b→II			
**-2403.6594089**			
Ru	0.244579	0.445391	0.442201
N	0.318450	-2.444810	-0.453058
N	2.312105	-1.519850	-0.182773
O	-2.005513	3.072740	-0.575281
C	0.953722	-1.260266	-0.141734
C	1.265344	-3.512023	-0.835832
H	1.243637	-3.674628	-1.938638
H	0.989126	-4.469879	-0.350013
C	2.608004	-2.954558	-0.350731
H	2.924336	-3.403054	0.618819
H	3.432102	-3.101671	-1.078331
C	-1.082948	-2.719710	-0.594533
C	-1.711348	-2.506906	-1.850192
C	-3.071051	-2.850912	-1.980774
H	-3.568405	-2.681462	-2.950743
C	-3.810490	-3.395876	-0.910456
C	-3.149472	-3.601311	0.316830
H	-3.708176	-4.032522	1.164624
C	-1.789489	-3.277512	0.500925
C	-0.958582	-1.876573	-3.000043
H	-1.612024	-1.763181	-3.886870
H	-0.079050	-2.481658	-3.308265
H	-0.574008	-0.871303	-2.723482
C	-5.279414	-3.719094	-1.068968
H	-5.509769	-4.088672	-2.089114
H	-5.904612	-2.814224	-0.902326
H	-5.611633	-4.485413	-0.340066
C	-1.113413	-3.501020	1.832995
H	-0.231557	-4.171006	1.740802
H	-1.810622	-3.964116	2.558416
H	-0.741575	-2.545274	2.262781
C	3.406071	-0.590572	-0.049763
C	3.924413	0.012203	-1.228749
C	5.015108	0.891618	-1.105088
H	5.410225	1.373293	-2.015360
C	5.614542	1.170201	0.139802
C	5.121777	0.499941	1.274671
H	5.603010	0.668738	2.252810
C	4.037437	-0.400055	1.207795
C	3.337456	-0.288746	-2.587424
H	3.961575	0.148554	-3.391324
H	2.317236	0.139671	-2.673087
H	3.259005	-1.381099	-2.773781
C	6.747169	2.165830	0.251131
H	7.379665	1.970860	1.140632
H	6.353800	3.201857	0.348559
H	7.397607	2.150059	-0.647463
C	3.630293	-1.176809	2.438093
H	2.530078	-1.219017	2.563867
H	4.056868	-0.717215	3.351333
H	4.013096	-2.221158	2.391760
C	-1.543264	0.299694	0.076632
H	-1.961615	-0.529579	-0.530041
C	-2.532388	1.180816	0.751303
C	-2.751556	2.558862	0.439083
C	-3.717609	3.291597	1.169911
H	-3.896310	4.351893	0.944420
C	-4.465601	2.674895	2.185276
H	-5.209426	3.269227	2.739980
C	-4.278299	1.315545	2.484981
H	-4.869704	0.826858	3.274459
C	-3.321426	0.585135	1.766603
H	-3.154320	-0.478983	1.996180
C	-2.228034	4.421649	-1.050926
H	-2.376294	5.088499	-0.169834
C	-3.464403	4.470181	-1.955967
H	-3.310554	3.821584	-2.843114
H	-3.644150	5.506527	-2.309827
H	-4.376937	4.124962	-1.429445
C	-0.950742	4.845754	-1.771389
H	-0.074127	4.753464	-1.101752
H	-1.039692	5.898253	-2.110610
H	-0.767977	4.201965	-2.655307
Cl	1.084659	1.884074	-1.171330
Cl	0.290579	-0.080516	2.721024

II			
**-2403.6916615**			
Ru	-0.147873	0.501707	-0.043609
N	0.721178	-2.431875	0.006600
N	-1.464141	-2.125052	-0.010992
O	-0.039941	2.847635	-0.109849
C	-0.251321	-1.457446	-0.003516
C	0.173186	-3.799047	0.114941
H	0.363462	-4.211943	1.132407
H	0.660007	-4.476020	-0.617251
C	-1.320459	-3.584376	-0.161310
H	-1.608867	-3.898971	-1.190225
H	-1.977757	-4.122569	0.552672
C	2.146272	-2.281455	0.067519
C	2.789356	-2.185316	1.328281
C	4.195927	-2.090265	1.347526
H	4.705619	-2.002924	2.321991
C	4.964265	-2.094632	0.165593
C	4.287766	-2.188953	-1.068465
H	4.869138	-2.180964	-2.006021
C	2.884393	-2.287086	-1.144237
C	1.992923	-2.125497	2.610946
H	2.663600	-2.060547	3.489903
H	1.351839	-3.022969	2.745855
H	1.314279	-1.244559	2.625340
C	6.474931	-2.028578	0.218541
H	6.919421	-3.048384	0.200155
H	6.831717	-1.533978	1.144470
H	6.891954	-1.479104	-0.650068
C	2.176809	-2.343220	-2.477769
H	1.562456	-3.263560	-2.581162
H	2.902596	-2.328747	-3.314250
H	1.483486	-1.482794	-2.600155
C	-2.783791	-1.547139	-0.027218
C	-3.464500	-1.365024	1.211480
C	-4.758515	-0.811666	1.186738
H	-5.283042	-0.657761	2.144962
C	-5.406541	-0.471201	-0.018428
C	-4.742337	-0.752565	-1.226010
H	-5.251803	-0.547722	-2.182915
C	-3.448163	-1.313103	-1.261599
C	-2.846837	-1.792518	2.520612
H	-3.586890	-1.728805	3.342496
H	-1.980257	-1.146577	2.778102
H	-2.477055	-2.839079	2.475809
C	-6.777407	0.167390	-0.007224
H	-7.294867	0.047340	-0.980299
H	-6.706197	1.258726	0.195949
H	-7.423787	-0.265287	0.784240
C	-2.864540	-1.739212	-2.588332
H	-1.759919	-1.730270	-2.583793
H	-3.194909	-1.065724	-3.403386
H	-3.215805	-2.763918	-2.847253
C	1.675362	0.797323	-0.044151
H	2.442181	0.001922	-0.000074
C	2.190192	2.151959	-0.131239
C	1.268577	3.241906	-0.168925
C	1.720963	4.568406	-0.270145
H	1.016914	5.411008	-0.300577
C	3.106186	4.815559	-0.333076
H	3.457948	5.856528	-0.413025
C	4.036509	3.760137	-0.292218
H	5.115887	3.971356	-0.338852
C	3.577648	2.439813	-0.192075
H	4.287393	1.597334	-0.161633
C	-1.124587	3.835467	-0.004401
H	-0.884651	4.637381	-0.737892
C	-1.185774	4.395562	1.417009
H	-1.399358	3.575537	2.131368
H	-1.990677	5.156580	1.484295
H	-0.233450	4.876478	1.716826
C	-2.412360	3.143567	-0.435224
H	-2.305523	2.694458	-1.441654
H	-3.233175	3.889528	-0.457276
H	-2.691859	2.345191	0.282795
Cl	-0.628409	0.795796	2.255043
Cl	-0.545601	0.643814	-2.367707

2a→2c’			
**-4735.1742699**			
Ru	-0.04204400	-0.09887200	0.27285300
Cl	0.16716300	4.35404200	2.29155200
Cl	3.01968700	6.62364700	-1.70700400
Cl	-0.48675500	-4.99941000	-1.08086500
Cl	4.68519800	-4.45260900	-2.71388500
O	0.17047100	1.93822200	0.62865800
O	-0.15827300	-2.14925100	-0.14139600
N	1.08949000	1.78690600	-2.12104800
N	0.54609900	-0.65920200	3.08938000
N	-1.64406400	-0.52546000	2.81766900
C	-4.93192000	1.05161000	2.02732200
H	-5.39444600	2.05272800	2.04546700
C	-2.98464300	-0.36006700	2.33077100
C	-3.57623700	0.93004400	2.37900900
C	-5.09304300	-1.32930900	1.63088900
H	-5.68519100	-2.21366200	1.34142200
C	-3.73562000	-1.50726900	1.96622200
C	-3.09371500	-2.87303800	1.89810400
H	-2.63906900	-3.17118500	2.86723400
H	-2.27719500	-2.90035800	1.14331800
H	-3.83752400	-3.64768200	1.62782500
C	-5.71097000	-0.06414900	1.65616700
C	-2.76199300	2.13318300	2.78328500
H	-3.34039400	3.06830400	2.65342200
H	-1.82953800	2.21417900	2.18739900
H	-2.45308400	2.08040200	3.85079900
C	-7.17206800	0.10609200	1.30420700
H	-7.30523400	0.85201100	0.49216700
H	-7.75619900	0.47415500	2.17539300
H	-7.62885400	-0.84695100	0.97130400
C	-0.44493300	-0.46092100	2.15138400
C	0.02571200	-0.71582300	4.46912400
H	0.29937100	0.20855500	5.02601400
H	0.46156200	-1.57896500	5.01286900
C	-1.48838900	-0.84262700	4.25266400
H	-1.86983200	-1.86701600	4.46609000
H	-2.07558900	-0.13248200	4.87029100
C	3.82753700	-2.36621300	2.55885900
H	4.20772100	-3.38522900	2.37518400
C	2.43707400	-2.17585600	2.66643600
C	2.84708800	0.23234700	3.05860000
C	6.22917300	-1.54019000	2.55209600
H	6.54401500	-2.47872300	3.05356600
H	6.82256500	-0.70586700	2.97688700
H	6.51153500	-1.63829500	1.48064100
C	1.95603600	-0.85669900	2.88617300
C	4.23130500	-0.01665500	2.95653900
H	4.93021900	0.82721600	3.08571500
C	4.74316100	-1.30229400	2.69703100
C	1.49225100	-3.35342200	2.59497800
H	2.02753700	-4.27124600	2.28362300
H	0.66059200	-3.17868400	1.88318200
H	1.03456900	-3.56074000	3.58791000
C	2.33527100	1.62009300	3.35793300
H	1.47522300	1.88882700	2.71247000
H	3.12965800	2.37882800	3.21620500
H	1.98998200	1.70993300	4.41195700
N	2.03785500	-0.62080200	-0.34083500
C	3.12827000	0.15385000	-0.37382900
H	3.02755900	1.16299900	0.05398000
C	4.35048500	-0.28264100	-0.94011200
H	5.21503400	0.39759300	-0.94578600
C	0.88931400	-2.66858100	-0.72229900
C	3.30046500	-2.41757600	-1.45613800
C	2.10022200	-1.89391100	-0.85344400
C	4.43452600	-1.55683600	-1.49091000
H	5.36390100	-1.92343100	-1.95276300
C	0.93829200	-3.99313600	-1.22863700
C	3.25851800	-3.75369800	-1.95394600
C	2.09832000	-4.52076000	-1.83734800
H	2.07982600	-5.55101400	-2.22015600
C	1.27947800	2.90486800	-1.34813700
C	2.64430200	3.90305700	-3.14984700
H	3.22724600	4.74895200	-3.54420800
C	2.50282800	2.72338800	-3.85905000
H	2.97670700	2.57913700	-4.84149400
C	0.74925500	2.93430400	0.01742600
C	1.54466500	5.30598900	0.16278000
H	1.61027100	6.22896100	0.75549300
C	2.13473200	5.22956100	-1.09406600
C	1.69080000	1.70129000	-3.31058800
H	1.49604900	0.78246400	-3.88874600
C	0.87621400	4.18758900	0.69388700
C	2.02943200	4.04019600	-1.87220800
O	-3.86342200	-1.01039100	-1.92472000
C	-1.86439600	-0.11329700	-0.24697800
H	-2.29902100	-1.13184900	-0.25622100
C	-2.53900000	2.24316400	-0.88387900
H	-1.73807500	2.61531900	-0.23329600
C	-4.39576400	2.62660500	-2.38913700
H	-5.05728100	3.31453200	-2.94067000
C	-3.75373000	0.33599700	-1.82946700

Cl -0.067247 1.961950 2.156218			C -2.69538900 0.83428900 -0.97896200<br>C -3.37400600 3.13445800 -1.56587900<br>H -3.22770300 4.22059700 -1.46245400<br>C -4.58812100 1.24196400 -2.52209800<br>H -5.39339600 0.87332500 -3.17226300<br>C -4.70517000 -1.64003500 -2.92034400<br>H -4.70835900 -0.99663600 -3.83000200<br>C -4.03584500 -2.97145400 -3.25735200<br>H -3.00524100 -2.80723600 -3.62672900<br>H -4.61484200 -3.50725400 -4.03715100<br>H -3.97591800 -3.61713400 -2.35743200<br>C -6.13381500 -1.80628700 -2.39366500<br>H -6.13941800 -2.46176600 -1.49863000<br>H -6.77839200 -2.27152200 -3.16808100<br>H -6.58427700 -0.83434100 -2.10981300<br>H -0.13064400 0.42382500 -2.57515400<br>Cl -0.61283000 -0.54444100 -3.37863200

2c'	2c'→3a	3a→4a'	4a'
**-4735.2017062**	**-4735.1838533**	**-3799.8426415**	**-3799.8451169**
Ru -0.215338 -0.164989 -0.047750	Ru -0.480669 0.443578 -0.053971	Ru -0.710749 -0.045981 -0.718013	Ru -0.711252 -0.033474 -0.719843
Cl 0.862630 3.960946 2.242420	Cl 3.484576 -2.935267 3.159617	Cl 3.037118 5.906238 -0.269385	Cl 3.197697 5.836499 -0.402355
Cl 4.547795 6.166797 -1.061605	Cl 6.890734 -5.100964 -0.490137	Cl 0.284873 2.957273 3.372380	Cl 0.335229 3.091336 3.307028
Cl -1.912858 -4.851806 -0.949951	Cl -4.579929 1.258219 -2.814920	N -1.165155 -2.887385 0.385519	N -1.248449 -2.863221 0.376026
Cl 3.220535 -5.826947 -2.498762	Cl -5.267723 -4.130968 -2.371130	N -3.036960 -1.732200 0.355800	N -3.085815 -1.653850 0.374381
O 0.606516 1.841292 0.151991	O 2.152249 -1.676404 0.711412	N 0.272928 1.681036 -1.585051	N 0.338314 1.642338 -1.606471
O -0.855741 -2.121541 -0.250352	O -2.255381 0.803576 -0.959129	O -0.616262 1.365551 0.975933	O -0.589011 1.438857 0.967040
N 1.860258 1.963381 -2.417353	N 2.878087 -2.143579 -1.883393	O 3.441614 -0.258955 1.019134	O 3.384678 -0.300527 1.142566
N 0.726328 -0.433635 2.838051	N -2.275411 0.764474 2.455037	C -1.687494 -1.637835 0.123016	C -1.737483 -1.596693 0.126149
N -1.420411 0.074056 2.739998	N -0.509168 2.073560 2.521789	C -2.177634 -3.884022 0.794120	C -2.286740 -3.836032 0.776959
C -4.765043 1.661070 2.219627	C 3.198469 2.547758 2.572671	H -2.180108 -4.742065 0.088363	H -2.334361 -4.671809 0.046205
H -5.207109 2.671487 2.227960	H 4.103286 1.973559 2.835566	H -1.939353 -4.287930 1.802809	H -2.043507 -4.277831 1.768059
C -2.804525 0.233215 2.381139	C 0.787082 2.657276 2.327205	C -3.483436 -3.081982 0.761032	C -3.563683 -2.988600 0.793779
C -3.375056 1.532401 2.407124	C 1.947115 1.914080 2.681868	H -3.993103 -3.034396 1.747751	H -4.033757 -2.925367 1.799455
C -5.004714 -0.735125 2.032968	C 2.158069 4.608219 1.863556	H -4.217404 -3.479587 0.027824	H -4.339272 -3.357290 0.089287
H -5.640377 -1.627211 1.901819	H 2.236462 5.664433 1.554771	C 0.199120 -3.331690 0.458305	C 0.103665 -3.347217 0.423683
C -3.620722 -0.920623 2.211469	C 0.879561 4.018317 1.937216	C 0.762000 -4.071071 -0.617058	C 0.628642 -4.102631 -0.660110
C -3.029852 -2.307972 2.202320	C -0.336817 4.840656 1.572616	C 2.051638 -4.610433 -0.447754	C 1.914797 -4.658871 -0.518432
H -2.361997 -2.481213 3.072187	H -0.331183 5.818017 2.099145	H 2.498310 -5.174315 -1.284053	H 2.333691 -5.231837 -1.362902
H -2.414650 -2.473297 1.289962	H -1.283567 4.317929 1.804555	C 2.777831 -4.466611 0.751506	C 2.671843 -4.521409 0.661858
H -3.825342 -3.078018 2.227038	H -0.341204 5.055057 0.482693	C 2.176473 -3.756021 1.808239	C 2.100156 -3.809927 1.734292
C -5.599912 0.543061 2.034064	C 3.329523 3.891685 2.163780	H 2.717037 -3.648534 2.763751	H 2.660283 -3.715512 2.679770
C -2.519908 2.750212 2.648934	C 1.866874 0.484422 3.160131	C 0.892447 -3.189014 1.690352	C 0.821453 -3.226587 1.644565
H -3.024070 3.668841 2.290412	H 2.677037 0.265733 3.883986	C 0.001674 -4.293551 -1.901325	C -0.165486 -4.333779 -1.922419
H -1.539982 2.665421 2.142158	H 1.982582 -0.246706 2.326256	H 0.644620 -4.779801 -2.661107	H 0.460470 -4.817944 -2.697664
H -2.313485 2.894287 3.734002	H 0.899698 0.256413 3.649619	H -0.878635 -4.955753 -1.744103	H -1.033846 -5.004974 -1.738276
C -7.088698 0.705556 1.824639	C 4.692897 4.529693 2.025893	H -0.384286 -3.338898 -2.317852	H -0.567700 -3.383170 -2.332401
H -7.456783 1.670578 2.227607	H 5.411048 4.130596 2.771100	C 4.161939 -5.060956 0.889321	C 4.056747 -5.120659 0.764542
H -7.661231 -0.112386 2.308690	H 4.645828 5.631266 2.144440	H 4.186484 -6.117485 0.548794	H 4.092539 -6.141436 0.330272
H -7.343769 0.682595 0.742334	H 5.118570 4.324464 1.018709	H 4.898956 -4.507692 0.267190	H 4.798187 -4.508329 0.205681
C -0.321409 -0.222065 1.959837	C -1.151956 1.128737 1.748893	H 4.519246 -5.031950 1.938086	H 4.400912 -5.182834 1.816485
C 0.378711 -0.090630 4.234528	C -2.448402 1.516537 3.717585	C 0.272286 -2.444202 2.850091	C 0.228533 -2.495436 2.827459
H 0.829767 0.888670 4.512344	H -2.645505 0.823683 4.561652	H -0.698515 -2.889190 3.156805	H -0.736576 -2.943176 3.148290
H 0.766561 -0.858987 4.933495	H -3.325261 2.197569 3.636143	H 0.940495 -2.455785 3.733194	H 0.915131 -2.522473 3.695996
C -1.148363 -0.029492 4.188561	C -1.123944 2.272777 3.848896	H 0.061414 -1.383799 2.593019	H 0.015280 -1.430866 2.592358
H -1.631152 -0.944113 4.605109	H -1.252454 3.353693 4.066664	C -4.044032 -0.704487 0.408386	C -4.068965 -0.603564 0.435198
H -1.564473 0.844260 4.729480	H -0.461705 1.851245 4.639419	C -4.934912 -0.545940 -0.682054	C -4.963739 -0.427366 -0.649290
C 3.302318 -3.111180 2.429504	C -5.557062 -0.550432 1.225172	C -5.981404 0.386327 -0.550274	C -5.990469 0.524636 -0.511030
H 3.340574 -4.210626 2.349508	H -6.450226 -0.182468 0.693138	H -6.669489 0.529121 -1.400686	H -6.681017 0.681102 -1.357021
C 2.045565 -2.489088 2.537340	C -4.491322 0.345772 1.449022	C -6.168801 1.143247 0.622394	C -6.152061 1.288644 0.661304
C 3.189469 -0.305642 2.671073	C -3.289763 -1.469390 2.622592	C -5.277319 0.939957 1.693323	C -5.255286 1.070749 1.724564
C 5.838806 -3.080275 2.305174	C -6.651992 -2.842624 1.353590	H -5.409257 1.517843 2.623914	H -5.367534 1.653524 2.654741
H 6.686332 -2.408758 2.548824	H -6.853298 -3.524531 2.205731	C -4.213310 0.022046 1.613958	C -4.212427 0.128622 1.639442
H 5.987903 -3.445263 1.265298	H -6.409908 -3.478868 0.473823	C -4.754271 -1.327127 -1.961391	C -4.797593 -1.204959 -1.932537
H 5.902619 -3.968909 2.967552	H -7.589005 -2.299885 1.116295	H -5.472571 -0.989584 -2.734063	H -5.529190 -0.870710 -2.694027
C 1.997408 -1.069320 2.622179	C -3.339835 -0.143054 2.118219	H -3.724789 -1.210141 -2.362852	H -3.774978 -1.077899 -2.347966
C 4.425629 -0.980106 2.577719	C -4.377449 -2.324362 2.363754	H -4.920051 -2.417297 -1.812311	H -4.950393 -2.297007 -1.784556
H 5.354604 -0.385269 2.607057	H -4.335843 -3.361438 2.737061	C -7.271402 2.174074 0.714020	C -7.234841 2.339979 0.758518
C 4.507469 -2.377336 2.444049	C -5.516984 -1.890304 1.655327	H -7.608484 2.324333 1.760001	H -7.513832 2.547056 1.811704
C 0.786200 -3.319242 2.615108	C -4.584944 1.777437 0.975487	H -6.919086 3.160388 0.338687	H -6.892171 3.300654 0.314230
H 1.009386 -4.392871 2.462766	H -5.615305 2.019782 0.650804	H -8.152700 1.889300 0.103592	H -8.151256 2.038082 0.210803
H 0.034216 -3.011915 1.861870	H -3.899849 1.950299 0.118607	C -3.262948 -0.150660 2.776999	C -3.257080 -0.062058 2.795729
H 0.303027 -3.216304 3.612090	H -4.299937 2.500322 1.768565	H -2.265737 0.283249 2.549532	H -2.255675 0.359979 2.564744
C 3.158787 1.193627 2.840488	C -2.124682 -1.946642 3.459799	H -3.651282 0.354487 3.683246	H -3.632793 0.443347 3.707212
H 2.235358 1.633784 2.420001	H -1.149987 -1.631001 3.039474	H -3.096304 -1.219170 3.029693	H -3.101335 -1.133630 3.041953
H 4.032437 1.671434 2.353138	H -2.122733 -3.050846 3.545723	C 0.998865 -0.648282 -0.239414	C 0.976946 -0.673525 -0.191737
H 3.200109 1.480954 3.915079	H -2.178530 -1.540980 4.495433	H 1.156353 -0.752280 0.854882	H 1.100812 -0.755930 0.909036
N 1.691918 -1.254949 -0.480337	N -1.372116 -1.570530 -0.017400	C 2.246861 -0.719582 -0.988411	C 2.248780 -0.776123 -0.895193
C 2.936582 -0.788222 -0.600028	C -0.876965 -2.722208 0.444437	C 3.508705 -0.554154 -0.306805	C 3.490544 -0.617000 -0.176733
H 3.110937 0.239454 -0.245368	H 0.068848 -2.650411 1.007721	C 4.718038 -0.659165 -1.028755	C 4.720661 -0.747706 -0.857967
C 3.990987 -1.562682 -1.147124	C -1.507825 -3.965041 0.180768	H 5.685102 -0.536490 -0.522068	H 5.672997 -0.626893 -0.323531
H 4.997195 -1.125045 -1.229101	H -1.067187 -4.889181 0.584442	C 4.698217 -0.915366 -2.410660	C 4.740642 -1.027269 -2.235545
C 0.032034 -2.944756 -0.745978	C -2.909592 -0.286978 -1.299870	H 5.654554 -0.991950 -2.953353	H 5.712390 -1.123312 -2.746903
C 2.422288 -3.398796 -1.443428	C -3.192733 -2.791540 -1.128172	C 3.479917 -1.068563 -3.096031	C 3.542534 -1.179836 -2.955984
C 1.409377 -2.531989 -0.895611	C -2.494943 -1.576325 -0.803353	H 3.470448 -1.271418 -4.178184	H 3.565025 -1.402415 -4.034099
C 3.736388 -2.861673 -1.567829	C -2.660635 -4.001545 -0.594979	C 2.276999 -0.960718 -2.388426	C 2.319325 -1.044109 -2.288863
H 4.528214 -3.492920 -1.998988	H -3.167933 -4.950866 -0.824920	H 1.306808 -1.083865 -2.895772	H 1.363540 -1.163686 -2.823905
C -0.269141 -4.275136 -1.133987	C -4.043402 -0.269588 -2.146344	C 4.630809 0.058749 1.782790	C 4.553489 -0.004623 1.944416
C 2.031006 -4.716749 -1.822022	C -4.343243 -2.684793 -1.963902	H 5.319563 0.641766 1.128778	H 5.276121 0.561934 1.312763
C 0.711479 -5.141925 -1.663456	C -4.749705 -1.450159 -2.468311	C 4.169298 0.952090 2.932598	C 4.071912 0.901411 3.076095
H 0.421923 -6.160534 -1.958197	H -5.627754 -1.383874 -3.126186	H 3.442413 0.416008 3.576441	H 3.317234 0.379848 3.699669
C 2.137351 2.876554 -1.442150	C 3.609460 -2.699434 -0.874759	H 5.035297 1.251441 3.557526	H 4.923133 1.189250 3.726192
C 3.764215 3.851394 -3.017337	C 5.055259 -3.658426 -2.609139	H 3.678897 1.868352 2.549325	H 3.607906 1.823854 2.675354
H 4.517232 4.621073 -3.245209	H 5.927552 -4.269140 -2.889403	C 5.327136 -1.217699 2.263603	C 5.206854 -1.292766 2.454209
C 3.444174 2.867336 -3.947941	C 4.266516 -3.051634 -3.584156	H 5.605030 -1.879621 1.419364	H 5.501717 -1.964088 1.623304
H 3.935223 2.826556 -4.930549	H 4.493632 -3.164894 -4.653640	H 6.252907 -0.965328 2.821072	H 6.116896 -1.056556 3.043729
C 1.427839 2.775292 -0.164546	C 3.152011 -2.425420 0.493539	H 4.658677 -1.788001 2.940438	H 4.503117 -1.846157 3.109116
C 2.655855 4.887819 0.438754	C 5.067753 -3.905201 1.166943	C 0.553531 1.898012 -2.869709	C 0.636705 1.814237 -2.894269
H 2.827403 5.678253 1.183228	H 5.627043 -4.380564 1.985838	H 0.152224 1.149543 -3.573980	H 0.225970 1.052028 -3.578217
C 3.367013 4.899853 -0.764915	C 5.480194 -4.105897 -0.155781	C 1.323911 3.008928 -3.300304	C 1.435239 2.893049 -3.352598
C 2.454339 1.922118 -3.618234	C 3.150915 -2.285613 -3.185984	H 1.522757 3.140300 -4.374138	H 1.645819 2.985467 -4.428229
H 2.100521 1.123540 -4.287972	H 2.458764 -1.777479 -3.874074	C 1.835045 3.895074 -2.361893	C 1.960110 3.795819 -2.438094
C 1.722536 3.876283 0.719250	C 3.949902 -3.108275 1.481145	H 2.460958 4.750318 -2.657225	H 2.609841 4.625654 -2.753306
C 3.120069 3.902679 -1.744454	C 4.754337 -3.512756 -1.223298	C 1.539082 3.700265 -0.982268	C 1.645763 3.650934 -1.056732
O -4.243343 -0.071707 -1.970263	O 0.557949 4.532248 -2.037462	C 0.710801 2.577286 -0.634212	C 0.784279 2.561342 -0.681393
C -2.036592 0.300902 -0.353205	C 0.134555 2.159962 -0.615340	C 0.288768 2.345478 0.718513	C 0.347675 2.395415 0.673819
H -2.710246 -0.580104 -0.314558	H -0.641716 2.924547 -0.836767	C 0.770581 3.204761 1.714552	C 0.842218 3.268416 1.647552
C -2.192248 2.761067 -0.891339	C 2.594460 1.735214 -0.814130	C 1.633162 4.289137 1.403136	C 1.739582 4.316888 1.310044
H -1.290928 2.909314 -0.281424	H 2.445777 0.802311 -0.245731	H 1.991391 4.941728 2.211175	H 2.109827 4.985304 2.099315
C -3.976441 3.610778 -2.289503	C 4.038741 3.253107 -2.023151	C 1.996083 4.538363 0.084418	C 2.117513 4.510553 -0.012986
H -4.489284 4.451358 -2.785180	H 5.036187 3.530929 -2.401449	Cl -1.134398 -1.013414 -2.852141	Cl -1.103313 -1.035954 -2.837060
C -3.846577 1.216044 -1.827427	C 1.661033 3.768425 -1.817646	H -1.653464 1.756232 0.442320	H -1.535827 1.782747 0.475181
C -2.677393 1.432872 -1.009293	C 1.458205 2.555885 -1.062027	Cl -2.676792 2.014031 -0.708221	Cl -2.598846 1.928178 -0.844837
C -2.829147 3.843617 -1.508237	C 3.869650 2.070340 -1.278717		
H -2.435300 4.864594 -1.386690	H 4.729519 1.416073 -1.068511		
C -4.483801 2.311614 -2.453224	C 2.949627 4.098318 -2.293321		
H -5.378025 2.159702 -3.073753	H 3.116853 5.017384 -2.871775		

C	-5.259144	-0.459518	-2.924443	C	0.577186	5.644896	-2.965873				
H	-5.193938	0.222437	-3.803255	H	1.289391	5.401963	-3.787228				
C	-4.897733	-1.875679	-3.370152	C	-0.833353	5.736910	-3.545878				
H	-3.884997	-1.895074	-3.818313	H	-1.116944	4.786512	-4.039453				
H	-5.627592	-2.242070	-4.120833	H	-0.889706	6.554491	-4.293226				
H	-4.901103	-2.570889	-2.505917	H	-1.572433	5.945274	-2.744933				
C	-6.650908	-0.362101	-2.292476	C	1.018548	6.927995	-2.255258				
H	-6.721336	-1.040274	-1.417170	H	0.301732	7.189419	-1.449380				
H	-7.431049	-0.656848	-3.024625	H	1.053367	7.774194	-2.972352				
H	-6.873917	0.668671	-1.950159	H	2.024250	6.821436	-1.801486				
Cl	-0.155968	-0.139245	-2.637168	Cl	0.365893	-0.473978	-2.228259				
H	1.073653	1.220934	-2.285209	H	2.010182	-1.566073	-1.635093				

4a'→5b	2a→2c"	2c"	2c"→3b
**-3799.8261078**	**-4735.1738349**	**-4735.2086666**	**-4735.1871175**
Ru -1.133887 0.024220 0.839864	Ru 0.22008200 -0.18282000 -0.23935100	Ru -0.167885 -0.501138 -0.239859	Ru -0.690562 -0.458793 -0.135859
Cl 4.867908 -4.464121 -1.141208	Cl 3.82020400 2.56797100 -2.57520700	Cl -3.417712 -4.345408 0.231593	Cl -5.498707 -1.929909 -0.505750
Cl 0.417699 -2.300671 -3.349814	Cl 2.64329400 6.18950500 1.29863300	Cl -2.416442 -3.722329 5.553705	Cl -5.351484 -1.669732 4.938208
N -1.468299 2.294865 -1.182703	Cl -3.65880100 -1.26623200 2.33628200	Cl 2.025657 3.572051 -1.355493	Cl 4.221080 2.793819 -1.048442
N -3.262654 1.030995 -0.895545	Cl -6.06381600 3.47937300 1.31152300	Cl 6.063252 3.669530 2.273798	Cl 8.006267 0.353410 1.986622
N 1.071578 -2.323948 1.733946	O 1.78247400 0.80881000 -1.11211600	O -1.637854 -1.909781 -0.054499	O -2.640007 -0.950050 -0.304138
O -0.454020 -1.818097 -0.535919	O -1.76522600 -0.48778600 0.18911900	O 1.599214 0.768422 -0.425553	O 2.475129 0.388416 -0.277616
O 3.312767 0.821419 -0.013703	N 0.32445000 1.51980900 1.00571000	N -0.320371 -0.643809 1.892293	N -1.275890 -0.427661 1.920479
C -1.949674 1.191490 -0.512472	N -0.79978600 -2.47716900 -2.14626400	N 0.679154 -1.126432 -3.172884	N -0.259499 -1.815283 -2.873429
C -2.503578 2.966595 -1.998634	N 1.39362200 -2.45267100 -1.94505700	N -1.147301 0.092902 -3.106841	N -0.810987 0.298665 -3.117518
H -2.725377 3.970787 -1.573519	C 4.86711500 -1.09717300 -2.26376000	C -4.750103 0.627350 -2.271784	C -2.880882 3.396400 -2.847648
H -2.143895 3.119941 -3.038070	H 5.42925900 -0.42876600 -2.93671900	H -5.654958 0.021547 -2.095152	H -3.945956 3.677487 -2.789460
C -3.698904 2.009166 -1.909217	C 2.77332400 -2.15950700 -1.64221500	C -2.372280 0.749277 -2.743521	C -1.172133 1.679230 -2.947014
H -3.910545 1.492638 -2.872130	C 3.50349500 -1.31594700 -2.52503500	C -3.538928 -0.037657 -2.534044	C -2.546451 2.030115 -2.888133
H -4.636127 2.511191 -1.589616	C 4.79829000 -2.58949000 -0.36987100	C -3.686435 2.780877 -2.517334	C -0.549466 4.022840 -2.977577
C -0.206754 2.981092 -1.108136	H 5.30653400 -3.11094800 0.45911100	H -3.743210 3.882545 -2.527427	H 0.232375 4.799297 -3.028823
C -0.015617 4.009523 -0.144859	C 3.42931100 -2.85251000 -0.59389700	C -2.446541 2.166047 -2.786321	C -0.158803 2.669217 -3.022102
C 1.149727 4.794037 -0.235331	C 2.74813500 -3.90536100 0.25367900	C -1.246577 3.016290 -3.134650	C 1.296853 2.286753 -3.135911
H 1.301502 5.594318 0.508611	H 3.25233600 -4.88796400 0.12862500	H -1.244699 3.282603 -4.215587	H 1.483392 1.624984 -4.008821
C 2.103971 4.612617 -1.256918	H 1.68056600 -4.03376900 -0.00450400	H -0.292435 2.502965 -2.909059	H 1.660323 1.737242 -2.239423
C 1.881148 3.586747 -2.194775	H 2.80146600 -3.65039600 1.33251700	H -1.260187 3.969303 -2.569423	H 1.932652 3.184419 -3.259317
H 2.608734 3.428853 -3.008974	C 5.53156000 -1.70421900 -1.17780900	C -4.845920 2.034428 -2.240882	C -1.900508 4.409194 -2.882212
C 0.737604 2.764308 -2.144310	C 2.84307300 -0.68668800 -3.72461100	C -3.488958 -1.543603 -2.604700	C -3.618946 0.967856 -2.874070
C -1.033440 4.275358 0.934696	H 2.34231300 -1.44290500 -4.36675100	H -4.508637 -1.974647 -2.574328	H -4.622423 1.418209 -2.743663
H -0.732950 5.139459 1.558876	H 3.58508200 -0.15432800 -4.35022300	H -2.908782 -1.962580 -1.755429	H -3.453534 0.231063 -2.060677
H -2.035797 4.500626 0.510569	H 2.06970500 0.04210400 -3.41227900	H -2.998526 -1.894290 -3.537923	H -3.638175 0.392422 -3.826146
H -1.170395 3.392080 1.594411	C 6.98885800 -1.40815900 -0.90173700	C -6.154192 2.711251 -1.901498	C -2.294789 5.867064 -2.799625
C 3.318117 5.511056 -1.341401	H 7.43257700 -2.14275500 -0.20005100	H -7.019534 2.176674 -2.345193	H -3.206661 6.078896 -3.395659
H 3.023287 6.580361 -1.404820	H 7.11031700 -0.39981500 -0.44859100	H -6.178692 3.762102 -2.254279	H -1.485110 6.533204 -3.159816
H 3.960174 5.410684 -0.440202	H 7.59069100 -1.41459100 -1.83450600	H -6.311594 2.726070 -0.800395	H -2.521503 6.155889 -1.749721
H 3.941282 5.280389 -2.228591	C 0.24470600 -1.81660000 -1.53770500	C -0.210443 -0.535025 -2.314170	C -0.500637 -0.657451 -2.181397
C 0.524259 1.683629 -3.178207	C -0.35882200 -2.57395700 -3.04333900	C 0.348032 -0.938200 -4.602903	C -0.483506 -1.690380 -4.331185
H -0.397160 1.853373 -3.776614	H -0.65333300 -3.35338300 -4.09155000	H 0.148728 -1.923492 -5.078941	H -1.352108 -2.313935 -4.638720
H 1.375242 1.637733 -3.885585	H -0.85567800 -4.52241400 -2.74873000	H 1.205990 -0.479936 -5.137913	H 0.403122 -2.058235 -4.888435
H 0.412465 0.682277 -2.712935	C 1.15968600 -3.60245600 -2.84402800	C -0.885704 -0.030874 -4.556656	C -0.731577 -0.188740 -4.511521
C -4.170510 -0.044409 -0.576834	H 1.52266800 -4.54069500 -2.36879200	H -0.701585 0.970317 -5.003613	H 0.093274 0.324704 -5.054452
C -5.166506 0.159179 0.413258	H 1.72606100 -3.47221800 -3.79003300	H -1.768653 -0.467117 -5.071116	H -1.674543 0.036080 -5.052195
C -6.082359 -0.884037 0.653581	C -4.14211100 -3.17699100 -0.61141000	C 4.209847 -2.312791 -2.830275	C 2.055482 -4.626352 -2.058257
H -6.848705 -0.739967 1.433771	H -4.54705400 -3.68300000 0.28044300	H 5.230352 -1.892885 -2.845445	H 3.141659 -4.819715 -2.031416
C -6.051498 -2.092662 -0.066583	C -2.74724100 -3.10528500 -0.76648900	C 3.115086 -1.432889 -2.961179	C 1.607562 -3.333060 -2.389518
C -5.087488 -2.232394 -1.083998	C -3.08961600 -1.92638100 -2.90546100	C 1.599679 -3.380012 -2.808108	C -0.721739 -4.127316 -2.143927
H -5.063244 -3.158046 -1.683595	C -6.52704100 -2.67325700 -1.32787300	C 5.226061 -4.627629 -2.534829	C 1.664368 -7.046184 -1.375426
C -4.147344 -1.224327 -1.365532	H -7.09059000 -2.46418500 -2.25923600	H 5.122639 -5.539466 -3.159555	H 1.034580 -7.851463 -1.807138
C -5.262602 1.446991 1.194393	H -6.84277000 -1.92063900 -0.57239500	H 5.323275 -4.970995 -1.481222	H 1.645710 -7.180593 -0.271132
H -6.145892 1.435725 1.862672	H -6.84726000 -3.66513400 -0.94584900	H 6.177233 -4.129041 -2.810456	H 2.708767 -7.210312 -1.709984
H -4.354581 1.601139 1.815734	C -2.22293100 -2.43760200 -1.90713700	C 1.810872 -1.981428 -2.921643	C 0.211294 -3.097380 -2.417050
H -5.356395 2.333712 0.530567	C -4.48281300 -2.00868900 -2.69145300	C 2.725845 -4.214532 -2.700033	C -0.220871 -5.401247 -1.814040
C -7.010224 -3.216412 0.255747	H -5.15690900 -1.59037300 -3.45833900	H 2.568140 -5.301749 -2.599560	H -0.939398 -6.207030 -1.586581
H -7.270947 -3.807261 -0.646226	C -5.03239000 -2.61931200 -1.55185700	C 4.039258 -3.703318 -2.697924	C 1.160445 -5.673391 -1.763770
H -6.555767 -3.920761 0.987163	C -1.84142400 -3.71423100 0.27410100	C 3.343378 0.047099 -3.164212	C 2.588328 -2.220574 -2.674160
H -7.950258 -2.838593 0.706731	H -2.39650400 -4.42865000 0.91301700	H 4.369147 0.335256 -2.859118	H 3.631252 -2.593726 -2.646264
C -3.143933 -1.407665 -2.478719	H -1.42795200 -2.91935600 0.92855100	H 2.620638 0.651835 -2.582462	H 2.490072 -1.396625 -1.933316
H -2.108861 -1.479876 -2.087669	H -0.98309700 -4.25359000 -0.17811900	H 3.237691 0.334432 -4.234942	H 2.427234 -1.764739 -3.675168
H -3.353584 -2.334202 -3.048422	C -2.58223600 -1.36373500 -4.21235100	C 0.205458 -3.956077 -2.758136	C -2.209114 -3.868317 -2.184926
H -3.159748 -0.562688 -3.200327	H -1.53586400 -1.01447900 -4.14770500	H -0.318288 -3.629057 -1.834706	H -2.489056 -2.985608 -1.573924
C 0.602325 0.707194 0.648031	H -3.19910600 -0.50396700 -4.54343400	H 0.233437 -5.063163 -2.762568	H -2.773821 -4.743390 -1.808333
H 1.045102 0.589297 -0.367156	H -2.64462400 -2.12819200 -5.01947100	H -0.417973 -3.629172 -3.617735	H -2.567144 -3.668710 -3.219370
C 1.610128 1.039522 1.652461	N -1.72377200 1.98650300 -1.18459600	N 3.289648 -0.605657 1.379443	N 3.313216 -1.706364 1.288388
C 3.009069 1.086512 1.287251	C -1.65624800 3.20996400 -1.71807700	C 4.020481 -1.309952 2.250140	C 3.606255 -2.776249 2.038552
C 3.987237 1.368572 2.267133	H -0.83881800 3.38545400 -2.43701300	H 3.751703 -2.374053 2.336983	H 2.798003 -3.515908 2.141045
H 5.052179 1.397060 2.000139	C -2.59168600 4.23690400 -1.44314400	C 5.055705 -0.685457 2.975025	C 4.882805 -2.882179 2.628395
C 3.614908 1.612733 3.599045	H -2.47585800 5.22321900 -1.91652800	H 5.647316 -1.266910 3.696220	H 5.125704 -3.758433 3.245959
H 4.398129 1.830270 4.343498	C -2.67837400 0.41539700 0.41013000	C 2.570402 1.363605 0.157449	C 3.654742 0.410603 0.183837
C 2.261680 1.576434 3.977437	C -3.74759200 2.67882300 0.02055600	C 4.546920 1.409226 1.803257	C 5.495434 -0.734139 1.609761
H 1.966718 1.767415 5.020874	C -2.71562100 1.70233000 -0.28097000	C 3.466351 0.723312 1.121253	C 4.167920 -0.675061 1.034987
C 1.287677 1.285604 3.016564	C -3.65362900 3.95770400 -0.59847900	C 5.314989 0.802180 2.743788	C 5.813350 -1.868451 2.412459
H 0.226228 1.239604 3.301246	H -4.42497500 4.70943200 -0.37285400	H 6.126882 1.180683 3.276480	H 6.819223 -1.922018 2.857059
C 4.687584 0.787316 -0.468195	C -3.71202700 0.19348800 1.36335600	C 2.940071 2.724129 -0.128877	C 4.651865 1.431333 -0.035083
H 5.296263 0.260149 0.303104	C -4.77450000 2.33564400 0.95036100	C 4.779479 2.773377 1.477167	C 6.376917 0.342174 1.326287
C 4.691431 -0.044064 -1.748918	C -4.75143800 1.11277500 1.61246700	C 3.994316 3.404397 0.508957	C 5.948183 1.396500 0.512111
H 4.063986 0.437814 -2.526678	H -5.52212800 0.86889500 2.35703500	H 4.195294 4.451387 0.240585	H 6.638609 2.223241 0.290685
H 5.724254 -0.136834 -2.141770	C 1.23029700 2.44426700 0.53596400	C -1.128876 -1.691429 2.258859	C -2.572283 -0.849014 2.073033
H 4.296398 -1.061369 -1.562520	C 0.67048000 3.96652300 2.36346800	C -0.656434 -1.274217 4.620472	C -2.387470 -0.665198 4.500132
C 5.232974 2.201325 -0.689451	H 0.80136000 4.92725400 2.88343200	H -0.781387 -1.532402 5.682755	H -2.824670 -0.760117 5.505282
H 5.177030 2.816345 0.230056	C -0.20305300 2.99553300 2.83864600	C 0.120573 -0.193472 4.221045	C -1.079768 -0.233300 4.313905
H 6.294210 2.156598 -1.010672	H -0.78842700 3.15862900 3.75552900	H 0.628982 0.443250 4.960734	H -0.443584 0.029370 5.172410
H 4.648149 2.713927 -1.479171	C 1.97120000 2.02375900 -0.62544400	C -1.782966 -2.368866 1.168807	C -3.264747 -1.133255 0.848374
C 1.760485 -2.609588 2.829650	C 3.07084400 4.23777900 -0.55907800	C -2.777115 -3.878448 2.851479	C -5.224947 -1.738035 2.213862
H 1.312784 -2.286630 3.786717	H 3.79280500 4.93400800 -1.00852200	H -3.418334 -4.744363 3.068546	H -6.264884 -2.091235 2.254125
C 3.013826 -3.284348 2.812109	C 2.36502900 4.60702300 0.58496100	C -2.155741 -3.190017 3.895833	C -4.536379 -1.453845 3.395077
H 3.537901 -3.499569 3.755695	C -0.35591300 1.77806200 2.13050900	C 0.267877 0.095188 2.840814	C -0.550399 -0.127315 3.002815
C 3.559182 -3.649021 1.589560	H -1.04436600 0.99292400 2.47705200	H 0.873635 0.946807 2.493531	H 0.481781 0.208638 2.820206
H 4.530481 -4.161846 1.520788	C 2.88514900 2.96707800 -1.15461500	C -2.601643 -3.472278 1.510592	C -4.602241 -1.576686 0.955307
C 2.851635 -3.353256 0.389533	C 1.42467200 3.71368800 1.18044100	C -1.318691 -2.065444 3.635871	C -3.186276 -0.993848 3.365202
C 1.574560 -2.688164 0.523863	O 0.63392300 -2.46215900 3.52469000	O -1.371765 3.729635 0.854339	O 0.431289 3.642153 1.179311
C 0.785965 -2.379392 -0.643600	C 0.99092800 -1.25490400 1.07639100	C -1.125082 1.131012 -0.145714	C -0.679201 1.411156 -0.090343
C 1.317529 -2.682839 -1.901873	H 0.68841700 -2.32162600 1.06862700	H -0.607747 1.993575 -0.618395	H 0.312123 1.779659 -0.448180
C 2.576992 -3.324182 -2.048915	C 2.90397200 0.02931400 2.09641000	C -3.313796 0.622638 0.981670	C -2.914223 2.255136 0.645122
H 2.946586 -3.558826 -3.056386	H 3.06658800 0.50243000 1.11846700	H -3.217816 -0.402648 0.602051	H -3.348312 1.360727 0.177846
C 3.319856 -3.661446 -0.926409	C 3.57353200 -0.35674200 4.39330600	C -4.562435 2.336800 2.147611	C -3.158541 4.379046 1.777894
Cl -2.025716 1.271564 2.597643	H 4.23738400 -0.14672300 5.24735300	H -5.443576 2.657686 2.726894	H -3.792274 5.154327 2.238399
H -1.030359 -2.268121 0.214092	C 1.67867200 -1.58706400 3.46663600	C -2.412340 2.892649 1.126088	C -0.926311 3.581668 1.179659
Cl -2.267447 -2.079570 1.604159	C 1.84258900 -0.90516100 2.20976900	C -2.272514 1.534125 0.660950	C -1.502675 2.400659 0.590313
	C 3.76442200 0.30422000 3.16827400	C -4.446903 1.006818 1.709439	C -3.740803 3.224471 1.225980
	H 4.58731000 1.02480000 3.04466200	H -5.237190 0.273459 1.931439	H -4.832535 3.085212 1.243271
	C 2.54206400 -1.30164700 4.54503100	C -3.557537 3.276516 1.857184	C -1.765013 4.562926 1.754056
	H 2.41717800 -1.80632800 5.51325900	H -3.676164 4.308561 2.214977	H -1.339466 5.472383 2.200222

	C	0.36764000	-3.23359800	4.71979800	C	-1.406678	5.120317	1.253317	C	1.131060	4.797913	1.703710
	H	0.51587300	-2.57261200	5.60491500	H	-1.878925	5.188015	2.260153	H	0.593447	5.153155	2.612749
	C	-1.10550600	-3.62928400	4.64524400	C	0.048779	5.568682	1.365144	C	2.517433	4.307513	2.113343
	H	-1.74712300	-2.73173400	4.54644200	H	0.586413	4.971246	2.128066	H	2.440962	3.502368	2.870954
	H	-1.40306200	-4.18143100	5.55978700	H	0.100336	6.637457	1.657033	H	3.104127	5.142236	2.548482
	H	-1.28923600	-4.28218700	3.76730000	H	0.571196	5.437408	0.395929	H	3.064234	3.911871	1.234006
	C	1.30765900	-4.44028000	4.80766300	C	-2.214032	5.946746	0.247486	C	1.174790	5.912069	0.654145
	H	1.14642500	-5.11585200	3.94221300	H	-1.725774	5.924149	-0.748789	H	1.725157	5.564850	-0.244074
	H	1.11319500	-5.01529800	5.73657400	H	-2.279747	7.004486	0.576606	H	1.693819	6.805648	1.058672
	H	2.37264700	-4.13283900	4.81274000	H	-3.245528	5.556469	0.136315	H	0.156456	6.218203	0.340640
	H	-0.84422700	1.21244700	-2.66450800	Cl	1.416670	-2.544603	0.251473	Cl	0.463101	-2.536932	0.721243
	Cl	-0.28860600	1.25321300	-3.88931800	H	2.525363	-1.188483	0.842723	H	2.331939	-1.677089	0.867600

3b			
**-3339.0418481**			
Ru	0.238238	-0.439693	-0.532299
Cl	4.613762	5.024153	0.043732
Cl	2.634103	1.395440	3.597500
N	-1.390332	-2.773266	0.714416
N	0.712720	-3.245265	0.252621
N	1.259507	1.261504	-1.321604
O	1.195616	0.284599	1.173223
O	-3.435952	1.788001	-1.551901
C	-0.225849	-2.245522	0.230108
C	-1.247328	-4.177582	1.160940
H	-2.030411	-4.817260	0.702900
H	-1.369023	-4.241520	2.265799
C	0.174226	-4.545423	0.696162
H	0.800010	-4.973768	1.507686
H	0.169252	-5.273182	-0.145190
C	-2.605948	-2.078078	1.025479
C	-3.714776	-2.207930	0.152794
C	-4.928412	-1.594977	0.526978
H	-5.798189	-1.691254	-0.144908
C	-5.061220	-0.869202	1.726758
C	-3.932358	-0.753949	2.564473
H	-4.012647	-0.181079	3.503623
C	-2.699293	-1.350272	2.241845
C	-3.588300	-2.947931	-1.158560
H	-4.528124	-2.883956	-1.741274
H	-3.364295	-4.026781	-1.008941
H	-2.760239	-2.534112	-1.776152
C	-6.375374	-0.230209	2.118717
H	-7.148889	-0.368763	1.337303
H	-6.257927	0.860433	2.293593
H	-6.769179	-0.664333	3.063133
C	-1.502411	-1.200929	3.150538
H	-1.077339	-2.185116	3.441886
H	-1.772808	-0.662674	4.079723
H	-0.684200	-0.640370	2.649601
C	2.099841	-3.101100	-0.109786
C	2.547459	-3.504971	-1.398333
C	3.916690	-3.356148	-1.699655
H	4.267498	-3.652309	-2.702604
C	4.844259	-2.857898	-0.764200
C	4.376641	-2.534589	0.525111
H	5.091722	-2.185040	1.288793
C	3.019804	-2.659847	0.882276
C	1.607140	-4.105555	-2.414365
H	2.121873	-4.258657	-3.383136
H	0.730785	-3.444482	-2.587336
H	1.230070	-5.098121	-2.082777
C	6.298892	-2.669401	-1.132362
H	6.967801	-2.851166	-0.266352
H	6.489223	-1.628423	-1.475637
H	6.605239	-3.346051	-1.955617
C	2.569348	-2.346959	2.288736
H	1.997934	-1.394858	2.313202
H	3.439197	-2.239233	2.965579
H	1.911635	-3.142435	2.697786
C	-1.420557	0.377723	-0.362010
H	-2.235468	-0.128214	-0.920055
C	-1.825920	1.668192	0.188690
C	-2.926820	2.364793	-0.431365
C	-3.379719	3.590004	0.104151
H	-4.213052	4.131737	-0.364270
C	-2.759722	4.135666	1.241006
H	-3.126180	5.094493	1.642307
C	-1.685236	3.473594	1.860306
H	-1.206555	3.901158	2.754520
C	-1.224234	2.262541	1.328410
H	-0.399263	1.721390	1.811025
C	-4.548210	2.373400	-2.269240
H	-4.404176	3.478354	-2.302333
C	-5.873086	2.040402	-1.576698
H	-6.022126	0.941632	-1.546829
H	-6.722428	2.494925	-2.127423
H	-5.897865	2.417822	-0.534913
C	-4.462715	1.823475	-3.691882
H	-3.488234	2.078302	-4.153118
H	-5.272630	2.249386	-4.318391
H	-4.563497	0.718888	-3.689266
C	1.274106	1.693246	-2.590927
H	0.646981	1.117685	-3.292150
C	2.056384	2.804171	-2.990024
H	2.037591	3.122992	-4.042986
C	2.829373	3.479901	-2.051813
H	3.441539	4.350572	-2.331548
C	2.838740	3.040880	-0.695310
C	2.023652	1.899997	-0.371086
C	1.955171	1.334992	0.953012
C	2.714854	1.992295	1.952507
C	3.519872	3.116672	1.661548
H	4.094549	3.587900	2.471397
C	3.587666	3.631897	0.366076
Cl	-0.374393	-1.259700	-2.723476

3b→4b			
**-3799.8398724**			
Ru	0.580664	-0.063760	-0.570166
Cl	-2.155077	6.441440	-0.077642
Cl	0.291128	3.162820	3.506119
N	0.869002	-2.866498	0.627828
N	2.853457	-2.029494	0.177125
N	-0.250074	1.764013	-1.380248
O	0.686474	1.344605	1.129917
O	-3.645055	-0.995907	-1.595095
C	1.511563	-1.733412	0.175579
C	1.786811	-3.955971	1.018508
H	1.495054	-4.910659	0.533500
H	1.744282	-4.116247	2.120120
C	3.144585	-3.433344	0.544165
H	3.929101	-3.470829	1.328446
H	3.529114	-3.984386	-0.341931
C	-0.500294	-3.038290	1.015916
C	-1.355334	-3.822148	0.197646
C	-2.655666	-4.096714	0.667992
H	-3.331136	-4.694730	0.033408
C	-3.111653	-3.639811	1.919406
C	-2.225430	-2.884300	2.716476
H	-2.561856	-2.520408	3.701868
C	-0.920446	-2.578326	2.294135
C	-0.887370	-4.336695	-1.142687
H	-1.734684	-4.741819	-1.730245
H	-0.147006	-5.159814	-1.029533
H	-0.391318	-3.537872	-1.736402
C	-4.515212	-3.931481	2.401116
H	-5.034436	-4.654867	1.741337
H	-5.125193	-3.002593	2.431808
H	-4.513691	-4.347476	3.430839
C	0.008895	-1.768033	3.165608
H	0.924552	-2.337664	3.435510
H	-0.488458	-1.468222	4.108511
H	0.351856	-0.849500	2.643474
C	4.013638	-1.201679	-0.040647
C	4.690460	-1.238844	-1.285635
C	5.884353	-0.500348	-1.408698
H	6.408067	-0.509765	-2.379762
C	6.428431	0.238289	-0.340559
C	5.749070	0.216637	0.892491
H	6.160622	0.779576	1.747328
C	4.547008	-0.493176	1.066704
C	4.163294	-2.033856	-2.455637
H	4.769035	-1.843697	-3.363492
H	3.104569	-1.778628	-2.676951
H	4.200838	-3.129095	-2.262102
C	7.688029	1.055283	-0.522926
H	8.236044	1.185947	0.432451
H	7.446594	2.072570	-0.903264
H	8.377704	0.589617	-1.256502
C	3.830132	-0.465219	2.396325
H	2.884197	0.114062	2.331569
H	4.459608	0.006207	3.176458
H	3.555502	-1.482108	2.748610
C	-1.167168	-0.736843	-0.433652
H	-1.421608	-1.522901	-1.173749
C	-2.356799	-0.228265	0.247483
C	-3.648851	-0.412045	-0.368389
C	-4.815575	0.047117	0.283197
H	-5.803845	-0.086978	-0.177734
C	-4.723464	0.690498	1.528745
H	-5.645459	1.044761	2.017780
C	-3.475251	0.881511	2.147308
H	-3.405558	1.379252	3.126546
C	-2.316710	0.428656	1.504494
H	-1.338136	0.547831	1.988010
C	-4.867767	-1.214124	-2.338532
H	-5.532590	-0.331981	-2.190318
C	-5.573314	-2.485141	-1.856585
H	-4.926819	-3.370003	-2.028585
H	-6.522760	-2.634761	-2.411180
H	-5.811169	-2.437845	-0.775142
C	-4.458544	-1.279675	-3.808868
H	-3.953628	-0.343226	-4.117711
H	-5.351255	-1.427030	-4.450181
H	-3.757887	-2.121883	-3.982236
C	-0.593586	1.974182	-2.652734
H	-0.366945	1.143274	-3.343544
C	-1.211977	3.177791	-3.076661
H	-1.471426	3.302890	-4.138371
C	-1.491948	4.171541	-2.147684
H	-1.986680	5.110130	-2.439190
C	-1.125516	3.980446	-0.784749
C	-0.474447	2.744348	-0.441216
C	-0.008467	2.480076	0.888699
C	-0.250937	3.450516	1.871754
C	-0.923515	4.662164	1.568250
H	-1.099776	5.395012	2.367535
C	-1.343716	4.924013	0.268566
Cl	0.690538	-1.136365	-2.719980
H	1.805135	1.583744	0.541552
Cl	2.789310	1.697391	-0.607965

4b			
**-3799.8531705**			
Ru	-0.513121	-0.558023	-0.062487
Cl	-3.919411	5.856498	1.054516
Cl	0.525318	5.196904	-1.978466
N	1.112272	-3.165200	0.322113
N	-1.073291	-3.439245	0.194979
N	-2.010179	1.128615	-0.057316
O	-0.047547	2.356084	-1.785191
O	3.056374	1.788467	1.279800
C	-0.066144	-2.501695	0.124868
C	0.940235	-4.618592	0.532640
H	1.381933	-4.922522	1.505588
H	1.467310	-5.187844	-0.263603
C	-0.586621	-4.801942	0.485596
H	-0.914736	-5.509216	-0.306896
H	-1.000958	-5.165423	1.450299
C	2.453891	-2.660622	0.239875
C	3.126727	-2.272484	1.425999
C	4.472888	-1.866487	1.316625
H	5.004390	-1.552561	2.230547
C	5.158121	-1.862567	0.087038
C	4.463643	-2.288367	-1.064261
H	4.983862	-2.298631	-2.036612
C	3.117925	-2.692460	-1.014703
C	2.434994	-2.303635	2.767714
H	3.088753	-1.885145	3.557833
H	2.178645	-3.343465	3.069332
H	1.482381	-1.729774	2.757477
C	6.595971	-1.404569	-0.011256
H	7.061778	-1.291420	0.988303
H	6.660885	-0.421569	-0.526717
H	7.211759	-2.116954	-0.599654
C	2.399944	-3.141295	-2.266496
H	2.113407	-4.215004	-2.217388
H	3.044536	-3.015356	-3.158121
H	1.464815	-2.566264	-2.432389
C	-2.487354	-3.219629	0.030900
C	-3.320945	-3.096832	1.179577
C	-4.704856	-2.909856	0.978395
H	-5.352963	-2.804485	1.864883
C	-5.283386	-2.881318	-0.305977
C	-4.440672	-3.095832	-1.414164
H	-4.877845	-3.136358	-2.426003
C	-3.052073	-3.292068	-1.274890
C	-2.772684	-3.202927	2.581626
H	-3.556950	-2.966340	3.327353
H	-1.923246	-2.503916	2.737675
H	-2.417275	-4.234512	2.799022
C	-6.764922	-2.642468	-0.493178
H	-7.184674	-3.287903	-1.292148
H	-6.963519	-1.590125	-0.793679
H	-7.333010	-2.833050	0.439420
C	-2.210659	-3.604846	-2.487315
H	-1.643898	-2.706973	-2.815834
H	-2.847027	-3.939406	-3.329953
H	-1.465445	-4.399621	-2.279126
C	1.214643	0.115499	0.167949
H	1.441786	0.255691	1.245382
C	2.279843	0.658281	-0.670922
C	3.254612	1.519384	-0.036573
C	4.319617	2.062229	-0.790355
H	5.063497	2.715847	-0.314156
C	4.432731	1.780616	-2.160718
H	5.265744	2.221851	-2.732081
C	3.492193	0.953329	-2.800210
H	3.576877	0.740966	-3.877061
C	2.438432	0.410284	-2.059362
H	1.680380	-0.214047	-2.552371
C	3.876839	2.737552	2.000440
H	4.138402	3.571520	1.309423
C	5.153353	2.064656	2.514029
H	4.898267	1.252853	3.225810
H	5.793719	2.801420	3.042030
H	5.745165	1.624184	1.686980
C	2.997786	3.284126	3.124313
H	2.085239	3.758660	2.712907
H	3.553906	4.040640	3.714715
H	2.684347	2.467002	3.805996
C	-3.102310	0.605185	0.514139
H	-3.124760	-0.491562	0.630325
C	-4.176852	1.377314	1.008791
H	-5.045153	0.870851	1.453380
C	-4.065898	2.758077	0.986531
H	-4.836497	3.407124	1.428248
C	-2.936443	3.363047	0.370581
C	-1.940134	2.497256	-0.234541
C	-0.892604	3.071286	-1.044848
C	-0.768492	4.476161	-1.056047
C	-1.676405	5.325221	-0.382903
H	-1.537708	6.413644	-0.436426
C	-2.755921	4.779195	0.296615
Cl	-0.734638	-0.429816	2.352532
H	-0.348135	1.406068	-1.923649
Cl	-0.701090	-0.527036	-2.492587

4b→5b			
**-3799.8430264**			
Ru	-0.228018	0.635734	-0.208263
Cl	6.774327	-2.402123	1.637584
Cl	3.695175	-4.146951	-2.485960
N	-2.971160	1.938282	-0.343491
N	-1.271567	3.350840	-0.216412
N	2.647706	0.282912	-0.125821
O	2.227819	-1.621549	-2.123320
O	-1.992639	-2.839228	1.799256
C	-1.600479	2.007352	-0.246843
C	-3.601723	3.265698	-0.495196
H	-4.461381	3.372749	0.198420
H	-3.991246	3.387955	-1.531401
C	-2.451675	4.233313	-0.180651
H	-2.355672	5.050683	-0.925527
H	-2.555456	4.701769	0.823780
C	-3.835993	0.791745	-0.393569
C	-4.451787	0.361957	0.810596
C	-5.385300	-0.690982	0.743721
H	-5.868171	-1.030643	1.675561
C	-5.719076	-1.315897	-0.474124
C	-5.094068	-0.855279	-1.650577
H	-5.346284	-1.325964	-2.615820
C	-4.158136	0.197478	-1.641695
C	-4.086061	0.991446	2.135221
H	-4.658994	0.532239	2.964370
H	-4.292133	2.083423	2.150842
H	-3.001820	0.872031	2.354780
C	-6.707685	-2.459657	-0.524228
H	-6.188875	-3.428285	-0.694418
H	-7.433442	-2.335154	-1.354916
H	-7.280221	-2.549946	0.420514
C	-3.520682	0.672550	-2.924803
H	-3.718970	1.750288	-3.110433
H	-3.911274	0.107481	-3.793442
H	-2.416425	0.553872	-2.899154
C	0.038524	3.925768	-0.045325
C	0.491910	4.280595	1.256474
C	1.764946	4.875025	1.379783
H	2.125673	5.136636	2.389029
C	2.574267	5.161469	0.263291
C	2.056607	4.877783	-1.015753
H	2.646126	5.144598	-1.908936
C	0.790771	4.286341	-1.200261
C	-0.374589	4.120360	2.483618
H	0.246883	4.021992	3.395097
H	-1.016826	3.222168	2.424125
H	-1.018520	5.018134	2.623845
C	3.953231	5.760096	0.427176
H	4.179131	6.492204	-0.375174
H	4.736879	4.972477	0.373539
H	4.065947	6.271552	1.404290
C	0.247259	4.094867	-2.594552
H	0.245964	3.022076	-2.883035
H	0.857112	4.652620	-3.331903
H	-0.801847	4.447996	-2.677990
C	-1.356042	-0.809763	0.088766
H	-2.135652	-0.697199	0.866579
C	-1.203604	-2.195108	-0.345660
C	-1.593871	-3.245668	0.566290
C	-1.507555	-4.595100	0.164349
H	-1.794557	-5.403264	0.851129
C	-1.041608	-4.919610	-1.121831
H	-0.975642	-5.980298	-1.413852
C	-0.658550	-3.912988	-2.024371
H	-0.289035	-4.171289	-3.027796
C	-0.731694	-2.572169	-1.629732
H	-0.443570	-1.771164	-2.324398
C	-2.334100	-3.787780	2.837894
H	-1.629669	-4.648704	2.770009
C	-3.775462	-4.277866	2.669258
H	-4.479975	-3.423829	2.739731
H	-4.031060	-5.008180	3.464680
H	-3.930711	-4.770406	1.688536
C	-2.099181	-3.061070	4.160783
H	-1.052500	-2.705506	4.231480
H	-2.303996	-3.739136	5.014153
H	-2.767619	-2.179693	4.244306
C	2.961748	1.267178	0.714828
H	2.284885	2.136585	0.745479
C	4.092343	1.230473	1.573425
H	4.291781	2.078978	2.244345
C	4.898457	0.103021	1.574359
H	5.763177	0.010922	2.248911
C	4.617644	-0.962600	0.673737
C	3.479496	-0.802750	-0.205307
C	3.203909	-1.783920	-1.225368
C	4.013372	-2.931568	-1.272169
C	5.094866	-3.124418	-0.377333
H	5.699126	-4.038487	-0.456452
C	5.398895	-2.154867	0.569267
Cl	0.095953	0.817160	2.154498
H	1.785315	-0.731369	-2.030932
Cl	0.152434	0.633915	-2.565929

**4b’**	**2a”H+**	**2a”H+→2a”**	**2a”**
**-3799.84340738**	**-4274.8311272**	**-4735.1929981**	**-4735.208667**
Ru 1.008631 0.105106 0.742062	Ru 0.077931 -0.032362 -0.207696	Ru -0.16696800 -0.13602100 0.12735200	Ru -0.058526 -0.144576 -0.023238
Cl -4.798549 -4.858054 -1.412922	Cl 1.195770 3.738351 -3.360624	Cl -0.19710400 3.93716100 3.14885300	Cl -0.779113 4.181913 2.640768
Cl -0.221324 -2.559277 -3.179006	Cl 1.158776 6.742111 1.178865	Cl 0.25118600 6.66830300 -1.54415600	Cl -0.969520 6.475157 -2.294313
N 1.631819 1.589959 -1.932213	Cl -2.442292 -2.888933 3.318125	Cl 2.54450800 -4.24688700 -3.02577600	Cl 3.866755 -4.654886 -1.673745
N 3.153693 0.151459 -1.214097	Cl -6.551481 0.604211 2.422654	Cl 6.39732800 -0.44115400 -3.30122100	Cl 6.132327 -0.165203 -3.673187
N -1.664543 -2.255600 1.767111	O 0.674901 1.437303 -1.468634	O -0.11357400 1.48957500 1.36415400	O -0.220744 1.636343 1.093005
O 0.028104 -1.490425 -0.387141	O -1.087430 -1.340752 1.157643	O 1.33291100 -2.47624300 -0.92412200	O 2.126469 -2.849547 -0.198171
O -2.299475 2.318075 2.442998	N 0.267888 1.601565 1.177977	N 0.01180000 1.45663600 -1.30977700	N -0.161004 1.366559 -1.588048
C 1.937894 0.719655 -0.915760	N -1.055977 -1.858053 -2.445651	N 0.91339100 -1.87716100 2.46011100	N 1.167112 -1.282234 2.557000
C 2.729793 1.698083 -2.920904	N 1.139952 -1.937308 -2.391331	N -1.14793300 -2.43944000 1.93126400	N -0.495354 -2.566461 1.875011
H 3.258659 2.670996 -2.796301	C 4.718699 -0.850765 -2.418196	C -4.81259400 -1.72983700 2.02645700	C -4.178002 -3.093195 1.423063
H 2.332889 1.657886 -3.954897	H 5.344470 -0.077866 -2.894998	H -5.51300300 -1.08701100 2.58582300	H -5.121208 -2.671568 1.809900
C 3.616546 0.495865 -2.572011	C 2.537512 -1.846952 -2.057568	C -2.53562500 -2.43472400 1.55462100	C -1.743500 -3.062914 1.351815
H 3.455986 -0.362830 -3.263290	C 3.338326 -0.852519 -2.684608	C -3.43665800 -1.61573800 2.29112700	C -2.962031 -2.534977 1.863984
H 4.699158 0.735472 -2.571264	C 4.514855 -2.814971 -1.027714	C -4.39891100 -3.45800700 0.38810600	C -2.997596 -4.718592 0.091266
C 0.554653 2.539761 -2.019043	H 4.974680 -3.596412 -0.399829	H -4.77373800 -4.18927700 -0.34790300	H -3.004112 -5.590152 -0.585456
C 0.625691 3.757648 -1.288939	C 3.124959 -2.865177 -1.260485	C -3.00624600 -3.37724500 0.60495400	C -1.751711 -4.193059 0.490782
C -0.423005 4.687282 -1.447315	C 2.319822 -4.012761 -0.690700	C -2.06629800 -4.24905200 -0.18899600	C -0.490408 -4.862194 0.000308
H -0.377607 5.630094 -0.876376	H 2.456805 -4.936030 -1.295760	H -1.14471500 -4.50327800 0.37037000	H 0.391179 -4.635186 0.627499
C -1.502674 4.464487 -2.322922	H 1.234752 -3.793825 -0.660108	H -1.73669000 -3.70912500 -1.10718900	H -0.242436 -4.508503 -1.022124
C -1.504936 3.276576 -3.079202	H 2.650266 -4.256721 0.339093	H -2.56226800 -5.18917400 -0.50277100	H -0.622083 -5.962710 -0.040735
H -2.326368 3.090328 -3.791584	H 5.329486 -1.813111 -1.584557	C -5.31717300 -2.65100800 1.08454600	C -4.221246 -4.183314 0.533662
C -0.489245 2.309788 -2.953722	C 2.737054 0.156996 -3.631800	C -2.93500600 -0.66447300 3.35169800	C -2.983521 -1.460899 2.925907
C 1.799533 4.084091 -0.400345	H 3.513035 0.844094 -4.020629	H -3.74737500 0.00022800 3.70372200	H -3.945566 -0.912865 2.918576
H 1.730208 5.124210 -0.025630	H 1.951786 0.762395 -3.135812	H -2.10643600 -0.03237700 2.97485600	H -2.171122 -0.723455 2.795123
H 2.761762 3.988554 -0.948070	H 2.266456 -0.337888 -4.509275	H -2.54639600 -1.21018400 4.24024500	H -2.870405 -1.905802 3.940228
H 1.873757 3.401749 0.476440	C 6.814182 -1.760391 -1.310317	C -6.80796000 -2.78099500 0.86284500	C -5.537041 -4.803346 0.116494
C -2.630907 5.463843 -2.449640	H 7.393709 -1.603303 -2.243565	H -7.27861100 -1.79257900 0.67785500	H -5.771998 -5.697276 0.736008
H -2.355215 6.450468 -2.026147	H 7.178934 -2.690767 -0.831999	H -7.31004300 -3.21112800 1.75695700	H -5.513923 -5.143069 -0.939458
H -3.536152 5.110545 -1.908813	H 7.060576 -0.914191 -0.632750	H -7.04011900 -3.43829000 0.00154900	H -6.380918 -4.094110 0.233947
H -2.927508 5.614023 -3.508657	C 0.044564 -1.397633 -1.768750	C -0.13921100 -1.55216700 1.63303300	C 0.186674 -1.398216 1.593303
C -0.506192 1.080357 -3.830870	C -0.724955 -2.695131 -3.623048	C 0.65963900 -3.06390800 3.30683400	C 1.266306 -2.473589 3.428026
H 0.115457 1.228174 -4.742965	H -1.054368 -2.188987 -4.555920	H 0.83993000 -2.81658200 4.37480000	H 1.354539 -2.169655 4.491106
H -1.532541 0.853313 -4.181166	H -1.257952 -3.666848 -3.565062	H 1.35090700 -3.88876300 3.03231800	H 2.168849 -3.067657 3.164450
H -0.111271 0.186455 -3.311099	C 0.799257 -2.831810 -3.522683	C -0.80261300 -3.40029200 3.00151900	C -0.031126 -3.222476 3.117478
C 3.961018 -0.678385 -0.350001	H 1.128958 -3.869710 -3.300576	H -0.94541300 -4.44215300 2.64454900	H 0.121739 -4.307666 2.947972
C 4.982288 -0.055716 0.421426	H 1.328565 -2.505880 -4.441722	H -1.47568600 -3.25494400 3.87504200	H -0.797399 -3.110748 3.918102
C 5.786993 -0.869490 1.240600	C -4.492297 -2.512125 -1.112782	C 4.57810000 -1.09997600 2.57107000	C 4.130708 0.968404 3.083354
H 6.568610 -0.388939 1.852666	H -5.001413 -3.240338 -0.458910	H 5.52663400 -1.53012000 2.20660700	H 5.216128 0.969453 2.884564
C 5.622286 -2.266962 1.304166	C -3.101433 -2.635274 -1.307966	C 3.37536700 -1.76016000 2.25152400	C 3.374881 -0.150509 2.672891
C 4.647005 -2.855127 0.477751	C -3.191452 -0.689100 -2.829067	C 2.15212700 -0.05446900 3.55517200	C 1.376144 0.909918 3.653188
H 4.526250 -3.951548 0.479686	C -6.738708 -1.388364 -1.498578	C 5.91408000 0.78070500 3.63502800	C 4.372955 3.275756 4.130091
C 3.820023 -2.091743 -0.370891	H -7.253504 -0.872384 -2.333224	H 6.71101800 0.06089100 3.91571200	H 4.263064 4.091716 3.381845
C 5.210506 1.435012 0.379373	H -6.942828 -0.804333 -0.574007	H 5.81040800 1.51719500 4.45687500	H 5.452944 3.033671 4.196093
H 6.136591 1.706420 0.923115	H -7.211964 -2.381845 -1.362538	H 6.28057800 1.33045900 2.74025400	H 4.051677 3.691992 5.107146
H 4.355920 1.959289 0.860487	C -2.451662 -1.690787 -2.146102	C 2.15380500 -1.19112200 2.70284300	C 1.975915 -0.142195 2.906098
H 5.301968 1.819537 -0.658728	C -4.580055 -0.614695 -2.605063	C 3.38374600 0.57141100 3.83742400	C 2.172333 2.005264 4.033972
C 6.458341 -3.107037 2.242906	H -5.159310 0.163368 -3.129924	H 3.38418300 1.46196000 4.48857600	H 1.698713 2.831355 4.589401
H 6.598958 -4.137550 1.857762	C -5.252253 -1.509004 -1.745755	C 4.60686000 0.07821500 3.34283800	C 3.550298 2.067223 3.741807
H 5.965381 -3.193312 3.236442	C -2.363710 -3.798882 -0.681317	C 3.42317800 -3.06582400 1.49090300	C 4.086393 -1.335072 2.061845
H 7.458929 -2.660125 2.413951	H -2.693030 -3.972744 0.363396	H 4.26988000 -3.07666000 0.77576400	H 4.887646 -1.007886 1.368621
C 2.859072 -2.779054 -1.308580	H -1.267254 -3.650557 -0.682397	H 2.49245900 -3.25684000 0.92447200	H 3.397472 -1.998877 1.509214
H 1.848355 -2.328145 -1.275567	H -2.572565 -4.740539 -1.234617	H 3.58796800 -3.91900200 2.18701000	H 4.581608 -1.939466 2.854450
H 2.765764 -3.854040 -1.057893	C -2.521374 0.256878 -3.799845	C 0.87872700 0.45742200 4.18327700	C -0.066013 0.829849 4.086448
H 3.215003 -2.719273 -2.361193	H -1.502862 0.546360 -3.471672	H 0.23220000 0.94760600 3.42692500	H -0.736767 0.845729 3.206200
C -0.411403 1.296857 0.773027	H -3.122560 1.176611 -3.940710	H 1.10310200 1.19869400 4.97451500	H -0.333350 1.684882 4.735747
H -0.197723 2.213459 1.364518	H -2.419422 -0.207578 -4.805998	H 0.29254000 -0.36665900 4.64185200	H -0.267413 -0.106083 4.650623
C -1.829098 1.165097 0.426200	N -2.037015 0.766444 -0.197099	N 2.23613600 0.02022300 -0.17169000	N 2.066717 0.039955 -0.362202
C -2.801147 1.759607 1.309506	C -2.469190 1.855744 -0.841155	C 2.77513800 1.14222300 0.30962400	C 2.401737 1.334693 -0.185694
C -4.176542 1.698364 0.994848	H -1.781699 2.280049 -1.589315	H 2.18736600 1.66925100 1.07591800	H 1.793225 1.880461 0.548542
H -4.923902 2.146632 1.664476	C -3.733573 2.445235 -0.592578	C 4.02367900 1.66242600 -0.11293400	C 3.426827 2.010706 -0.881984
C -4.603495 1.057134 -0.181000	H -4.025412 3.348039 -1.148846	H 4.39743700 2.59770200 0.32881000	H 3.581989 3.083957 -0.699214
H -5.680831 1.018870 -0.410239	C -2.351668 -0.919013 1.473915	C 2.43145300 -1.98708500 -1.51241200	C 2.942458 -2.178937 -1.034324
C -3.671850 0.476561 -1.058726	C -4.163059 0.716220 1.056529	C 4.21539500 -0.22805100 -1.61640600	C 4.014258 -0.103601 -1.890108
H -4.008399 -0.012191 -1.985782	C -2.852027 0.204872 0.755501	C 2.95351200 -0.71064200 -1.09259600	C 2.967851 -0.736527 -1.103952
C -2.306189 0.533130 -0.747318	C -4.585401 1.875100 0.344643	C 4.73057100 0.99193100 -1.09802400	C 4.189106 1.304981 -1.794726
H -1.560779 0.109657 -1.431658	H -5.576379 2.302555 0.556808	H 5.68785200 1.37202000 -1.48517300	H 4.968348 1.786987 -2.402787
C -3.142441 3.016424 3.388480	C -3.131786 -1.526497 2.455081	C 3.14207900 -2.69428400 -2.49996600	C 3.884113 -2.912209 -1.778639
H -4.083023 2.432462 3.523305	C -4.939107 0.040798 2.056557	C 4.87844600 -1.00447700 -2.61845500	C 4.902122 -0.917429 -2.665636
C -3.471314 4.424573 2.882906	C -4.435039 -1.057073 2.748433	C 4.35030400 -2.20904800 -3.06076600	C 4.847102 -2.300374 -2.612537
H -2.538808 5.012337 2.756495	H -5.036256 -1.557600 3.519713	H 4.86835100 -2.80069800 -3.82793100	H 5.545013 -2.919633 -3.191711
H -4.121278 4.953921 3.610021	C 0.577741 2.765910 0.514376	C 0.03063900 2.68497300 -0.69468000	C -0.375749 2.623043 -1.077148
H -3.998118 4.399839 1.907942	C 0.528017 4.012480 2.616014	C 0.23170800 3.79807500 -2.85876300	C -0.416555 3.551682 -3.338944
C -2.370048 3.021011 4.706486	H 0.626535 4.956455 3.172770	H 0.31444500 4.71600000 -3.46010900	H -0.523674 4.409906 -4.019499
H -2.143082 1.986809 5.032697	C 0.235073 2.817961 3.262390	C 0.21787300 2.54061800 -3.44878200	C -0.175651 2.270557 -3.818227
H -2.963051 3.520858 5.499141	H 0.096992 2.782265 4.353003	H 0.28900000 2.42597600 -4.54063300	H -0.081325 2.076664 -4.897198
H -1.408134 3.560977 4.591188	C 0.757806 2.634585 -0.905482	C -0.05199100 2.65169900 0.74111600	C -0.425431 2.706303 0.365228
C -2.454178 -2.503897 2.820877	C 1.143006 5.072212 -0.973073	C 0.02920700 5.12090200 0.69172200	C -0.843168 5.133616 0.080227
H -2.081169 -2.147131 3.792473	H 1.355775 5.968525 -1.572704	H 0.01830900 6.06752900 1.24999500	H -1.034700 6.108869 0.549519
C -3.662589 -3.204087 2.643009	C 0.995532 5.177354 0.411136	C 0.12908000 5.13052400 -0.70006200	C -0.766618 5.030676 -1.309931
H -4.316985 -3.394492 3.505318	C 0.108766 1.624806 2.505917	C 0.09729900 1.37842600 -2.64362400	C -0.056496 1.188688 -2.908577
C -3.984406 -3.670113 1.371092	H -0.122093 0.664414 2.993412	H 0.03079100 0.36021900 -3.06640400	H 0.106548 0.149248 -3.237595
H -4.904736 -4.248360 1.197788	C 1.024017 3.822605 -1.625325	C -0.06184600 3.90279400 1.40210000	C -0.675027 3.997267 0.900426
C -3.128248 -3.427044 0.257059	C 0.706562 4.023876 1.201807	C 0.13255500 3.91064400 -1.44088900	C -0.526894 3.771619 -1.934294
C -1.928515 -2.646272 0.486983	O 2.985332 -1.848501 2.697185	O -3.97086000 0.01930100 -2.08939000	O -4.180380 -0.278869 -1.833917
C -0.980156 -2.267472 -0.559532	C 1.697072 -0.755140 0.492581	C -2.03225000 -0.10672100 -0.16458600	C -1.980104 -0.261924 -0.180372
C -1.297937 -2.860142 -1.832079	H 1.624520 -1.831019 0.775613	H -2.36668500 -1.00360300 -0.72753300	H -2.291886 -1.104167 -0.831962
C -2.442419 -3.641853 -2.073443	C 3.457928 1.027646 0.466787	C -3.13545800 1.89759600 0.93083300	C -3.183816 1.490155 1.213704
H -2.617414 -4.031095 -3.086536	H 2.951079 1.461495 -0.405407	H -2.34150700 1.89768600 1.68909400	H -2.299375 1.556301 1.859932
C -3.362627 -3.911390 -1.057122	C 5.290378 0.986182 2.045652	C -5.20724700 2.78592200 0.04224200	C -5.439966 2.163179 0.651118
Cl 2.229837 1.631732 2.099437	H 6.218957 1.423020 2.446735	H -6.04083100 3.50535300 0.09205000	H -6.339770 2.766551 0.854221
H -0.703022 -1.815463 1.981742	C 3.583552 -0.755270 2.152183	C -4.11654800 0.91913000 -1.09054700	C -4.287864 0.526573 -0.748660
Cl 0.978943 -1.316635 2.720668	C 2.895281 -0.148726 1.036442	C -3.06837500 0.91796000 -0.09585600	C -3.123128 0.594727 0.109578
	C 4.641300 1.591241 0.952958	C -4.19044100 2.81426500 1.01408000	C -4.318287 2.257942 1.494799
	H 5.062332 2.493420 0.484380	H -4.21876900 3.55107700 1.83131400	H -4.324581 2.934998 2.362437
	C 4.774323 -0.177525 2.641267	C -5.17673500 1.84921900 -1.00371500	C -5.430415 1.306951 -0.461622
	H 5.309036 -0.619956 3.492753	H -5.98015400 1.85159100 -1.75328700	H -6.317967 1.258315 -1.107180

	C	3.594292	-2.566336	3.807902	C	-4.93143000	-0.10420600	-3.16641200	C	-5.262847	-0.434014	-2.783672
	H	4.012095	-1.816189	4.516585	H	-5.28504600	0.91632800	-3.44208300	H	-5.734558	0.562755	-2.945427
	C	2.457669	-3.313901	4.500400	C	-4.16167800	-0.70056200	-4.34296500	C	-4.608550	-0.888714	-4.086950
	H	1.674564	-2.612004	4.850530	H	-3.33743000	-0.02890000	-4.65308400	H	-3.863109	-0.144958	-4.431354
	H	2.844269	-3.866147	5.380312	H	-4.83976300	-0.85310200	-5.20744300	H	-5.374634	-1.013304	-4.879071
	H	1.993844	-4.049445	3.810686	H	-3.71018300	-1.67211800	-4.05835500	H	-4.088553	-1.857698	-3.942567
	C	4.705062	-3.493455	3.310483	C	-6.11776200	-0.96450400	-2.72290100	C	-6.303946	-1.426443	-2.258459
	H	4.288507	-4.275106	2.642001	H	-5.76938600	-1.98245600	-2.45342400	H	-5.839104	-2.421415	-2.104280
	H	5.193782	-4.000521	4.167391	H	-6.85942000	-1.05261800	-3.54354700	H	-7.133684	-1.536120	-2.987034
	H	5.485680	-2.939234	2.752732	H	-6.63114600	-0.53082600	-1.84123800	H	-6.737092	-1.095018	-1.293537
	H	-0.808591	-2.091550	1.732204	H	0.52112600	-2.45577400	-1.60118800	H	1.190201	-2.546335	-0.423406
					Cl	-0.83578400	-1.87753400	-2.58370300	Cl	-0.115800	-1.912837	-1.746317

2a”→3b			
-4735.209666			
Ru	-0.392321	-0.177560	0.088176
Cl	-0.117407	4.177291	2.773818
Cl	0.852702	6.395787	-2.106914
Cl	3.835189	-4.832192	-2.402946
Cl	6.877085	-0.385751	-3.005025
O	-0.167190	1.569227	1.222481
O	2.092670	-3.143282	-0.771183
N	0.087864	1.276069	-1.427896
N	0.454863	-1.682985	2.616654
N	-1.418903	-2.534232	1.821074
C	-5.067372	-2.331312	1.008232
H	-5.948678	-1.741823	1.312719
C	-2.681208	-2.770929	1.175482
C	-3.817670	-2.018029	1.578997
C	-4.087231	-4.137748	-0.256343
H	-4.189871	-4.981508	-0.960297
C	-2.812079	-3.861791	0.276051
C	-1.655629	-4.768438	-0.077838
H	-0.685494	-4.365644	0.262989
H	-1.583980	-4.903520	-1.175525
H	-1.801637	-5.776771	0.369170
C	-5.226186	-3.384881	0.087268
C	-3.724580	-0.953001	2.647433
H	-4.583580	-0.256399	2.591193
H	-2.795300	-0.358617	2.565877
H	-3.738713	-1.407642	3.663468
C	-6.578688	-3.718385	-0.503618
H	-6.845526	-4.782384	-0.328224
H	-6.589619	-3.567028	-1.604694
H	-7.382559	-3.091389	-0.069174
C	-0.502843	-1.523012	1.634584
C	0.279167	-2.938365	3.383613
H	0.433935	-2.753651	4.465579
H	1.019499	-3.699856	3.053352
C	-1.153275	-3.338987	3.033468
H	-1.262118	-4.420047	2.814118
H	-1.885447	-3.079372	3.833015
C	3.773562	-0.171857	3.490757
H	4.845279	-0.404800	3.370766
C	2.827988	-1.065763	2.946923
C	1.033302	0.370124	3.850254
C	4.430081	1.961433	4.712715
H	4.642784	2.763760	3.971579
H	5.392585	1.455592	4.930429
H	4.084367	2.463735	5.639246
C	1.450122	-0.754085	3.084549
C	2.018772	1.233880	4.364286
H	1.695057	2.114979	4.942433
C	3.395343	0.994160	4.182020
C	3.299755	-2.341058	2.289918
H	4.319849	-2.218280	1.874774
H	2.632346	-2.668245	1.469495
H	3.358223	-3.169071	3.031984
C	-0.422838	0.611366	4.162444
H	-0.973392	0.895265	3.244340
H	-0.537839	1.432120	4.895940
H	-0.905229	-0.296644	4.583000
N	2.457545	-0.373942	-0.202673
C	2.772485	0.860434	0.188748
H	2.079926	1.330682	0.905242
C	3.916547	1.570255	-0.259681
H	4.092264	2.597647	0.091634
C	3.111640	-2.451596	-1.298184
C	4.536419	-0.402148	-1.526559
C	3.340111	-1.049154	-1.015236
C	4.780669	0.946641	-1.143045
H	5.673880	1.454625	-1.536605
C	4.067559	-3.131836	-2.076253
C	5.438886	-1.153607	-2.342867
C	5.215024	-2.496001	-2.608696
H	5.917999	-3.073308	-3.224628
C	0.186685	2.548694	-0.920251
C	0.642584	3.425360	-3.154672
H	0.853660	4.270618	-3.827151
C	0.559921	2.122048	-3.630727
H	0.707043	1.897912	-4.697795
C	0.024983	2.652104	0.509884
C	0.351025	5.093036	0.239733
H	0.401841	6.085775	0.708762
C	0.530055	4.962813	-1.138646
C	0.271959	1.061507	-2.735938
H	0.173162	0.013405	-3.063190
C	0.104146	3.961853	1.049108
C	0.457925	3.682967	-1.764770
O	-4.126264	0.462539	-2.165129
C	-2.217872	0.053813	-0.246576
H	-2.678558	-0.796375	-0.793370
C	-3.083920	2.143171	0.914511
H	-2.289446	2.035239	1.664774
C	-5.028524	3.298808	0.047852
H	-5.769438	4.112011	0.114040
C	-4.161837	1.346499	-1.135216
C	-3.125006	1.195432	-0.142243
C	-4.022945	3.175576	1.022945
H	-3.964578	3.886605	1.860939
C	-5.102341	2.394590	-1.024541
H	-5.893713	2.519018	-1.776641

3b→4b’			
-3799.8231279			
Ru	-0.714918	-0.196891	0.588565
Cl	2.870001	6.037827	-0.587123
Cl	-0.473754	2.854536	-3.458330
N	-1.062751	-2.705477	-1.069811
N	-2.969045	-1.708653	-0.556648
N	0.314337	2.049310	1.551838
O	-0.619018	1.233148	-0.951777
O	3.291928	-1.143897	1.950899
C	-1.604763	-1.615412	-0.430731
C	-2.089140	-3.644059	-1.568874
H	-2.082591	-4.580770	-0.967740
H	-1.883391	-3.919744	-2.624050
C	-3.389186	-2.847112	-1.396525
H	-3.800912	-2.478129	-2.363369
H	-4.189987	-3.424683	-0.890898
C	0.308751	-2.993915	-1.370336
C	1.013524	-3.948461	-0.591405
C	2.319657	-4.295454	-0.994376
H	2.881055	-5.028435	-0.390617
C	2.923806	-3.741333	-2.139288
C	2.182905	-2.813569	-2.901706
H	2.634278	-2.375775	-3.807905
C	0.877640	-2.431746	-2.545604
C	0.390499	-4.567050	0.637605
H	1.149664	-5.108696	1.235546
H	-0.398103	-5.304821	0.369079
H	-0.087201	-3.799664	1.286113
C	4.332430	-4.115784	-2.543298
H	4.716862	-4.969109	-1.949679
H	5.028976	-3.261866	-2.396795
H	4.387325	-4.393249	-3.617138
C	0.091304	-1.458002	-3.390967
H	-0.840168	-1.920253	-3.784272
H	0.685703	-1.113988	-4.259832
H	-0.223546	-0.564552	-2.810653
C	-3.996844	-0.791649	-0.128972
C	-4.727082	-1.081971	1.052471
C	-5.790984	-0.226669	1.398305
H	-6.354896	-0.436395	2.322830
C	-6.150830	0.881026	0.606670
C	-5.424881	1.114751	-0.576997
H	-5.699921	1.966645	-1.221727
C	-4.351504	0.293335	-0.971470
C	-4.368176	-2.252297	1.933701
H	-5.090609	-2.355949	2.767088
H	-3.349153	-2.121686	2.360987
H	-4.359584	-3.212748	1.374252
C	-7.266555	1.810013	1.030274
H	-7.769308	2.274127	0.157241
H	-6.871162	2.639006	1.658108
H	-8.034292	1.282805	1.632532
C	-3.593925	0.589007	-2.243628
H	-2.567565	0.951406	-2.020881
H	-4.110273	1.369223	-2.836497
H	-3.488105	-0.308777	-2.889443
C	1.020008	-0.908280	0.442116
H	1.200977	-1.820918	1.046266
C	2.275918	-0.315741	-0.023093
C	3.471028	-0.517400	0.758524
C	4.713584	-0.031563	0.295572
H	5.628349	-0.179679	0.886164
C	4.789974	0.655576	-0.928068
H	5.767745	1.029819	-1.272356
C	3.637869	0.866485	-1.705468
H	3.702872	1.397246	-2.667478
C	2.402550	0.391356	-1.246366
H	1.499033	0.525182	-1.854267
C	4.407326	-1.482827	2.809183
H	5.121413	-0.626827	2.816175
C	5.111386	-2.745134	2.302700
H	4.408953	-3.603623	2.313025
H	5.975822	-2.993542	2.952586
H	5.486177	-2.618614	1.267273
C	3.819153	-1.647384	4.209152
H	3.328064	-0.711721	4.541915
H	4.617455	-1.903937	4.934859
H	3.058688	-2.454520	4.218223
C	0.921560	2.263384	2.731788
H	0.563768	1.647186	3.572090
C	1.914202	3.255410	2.867859
H	2.411220	3.401167	3.837786
C	2.206935	4.071927	1.777482
H	2.937249	4.891124	1.863029
C	1.562483	3.872429	0.522222
C	0.635039	2.769019	0.424929
C	0.021637	2.357086	-0.822602
C	0.254405	3.243052	-1.917742
C	1.099564	4.367416	-1.827263
H	1.255146	4.990858	-2.719113
C	1.769625	4.669270	-0.638988
Cl	-0.907383	-1.549049	2.572002
H	-0.919283	1.773759	1.682223
Cl	-2.462793	1.478432	1.797048

4b’→5b			
-3799.8243664			
Ru	1.248002	1.164948	-0.883755
Cl	-8.038500	-2.004028	-0.071862
Cl	-3.822558	-1.480114	3.339792
N	2.026087	0.679585	2.059980
N	1.325631	2.714763	1.535407
N	-3.486944	-0.059838	-1.576662
O	-2.385483	-0.351711	0.875584
O	3.214110	-2.800431	-2.056066
C	1.589663	1.462830	1.025071
C	2.186781	1.458680	3.307831
H	3.267278	1.655812	3.495440
H	1.787548	0.898118	4.176737
C	1.403145	2.743954	3.007298
H	0.382516	2.730638	3.454723
H	1.917871	3.661106	3.358969
C	2.453209	-0.691596	2.035228
C	3.774963	-1.011358	1.632657
C	4.171869	-2.364139	1.680418
H	5.196917	-2.622631	1.365668
C	3.312523	-3.380928	2.136750
C	2.020121	-3.016486	2.565322
H	1.335960	-3.795041	2.942625
C	1.566046	-1.684715	2.530403
C	4.746861	0.057030	1.191337
H	5.729209	-0.386099	0.935200
H	4.921579	0.805754	1.994628
H	4.379922	0.622294	0.307896
C	3.754116	-4.826891	2.183143
H	4.824630	-4.939788	1.919470
H	3.162044	-5.447463	1.475730
H	3.604073	-5.264790	3.192880
C	0.180348	-1.340340	3.025420
H	0.211523	-0.625470	3.876401
H	-0.343576	-2.248010	3.382253
H	-0.465139	-0.880839	2.247263
C	0.766088	3.811872	0.787874
C	1.623133	4.869836	0.376616
C	1.056833	5.943176	-0.337333
H	1.717706	6.761364	-0.669533
C	-0.319059	6.003051	-0.635046
C	-1.145956	4.966342	-0.161381
H	-2.231677	5.011500	-0.350294
C	-0.636748	3.870672	0.563473
C	3.099110	4.855241	0.690991
H	3.577476	5.804778	0.380453
H	3.598847	4.018886	0.156198
H	3.292664	4.716850	1.776039
C	-0.888491	7.140573	-1.452395
H	-1.949460	7.338597	-1.197481
H	-0.852142	6.901229	-2.538236
H	-0.315071	8.078664	-1.306842
C	-1.575164	2.826917	1.120107
H	-1.337627	1.794498	0.790993
H	-2.618026	3.036685	0.812304
H	-1.560839	2.816378	2.231557
C	1.909840	-0.551866	-1.143977
H	2.897417	-0.570900	-1.654002
C	1.296442	-1.859430	-1.003434
C	1.993173	-3.021730	-1.506178
C	1.384214	-4.294248	-1.428042
H	1.906028	-5.187311	-1.799129
C	0.103297	-4.431386	-0.867759
H	-0.352282	-5.433817	-0.815408
C	-0.594606	-3.310324	-0.380234
H	-1.597806	-3.419857	0.058535
C	-0.000830	-2.046999	-0.455945
H	-0.560252	-1.169917	-0.091686
C	3.922452	-3.833988	-2.782072
H	3.173198	-4.487567	-3.285198
C	4.781786	-4.664452	-1.824844
H	5.540596	-4.020101	-1.335437
H	5.311241	-5.467844	-2.377692
H	4.170242	-5.136695	-1.030729
C	4.745672	-3.112650	-3.848661
H	4.090495	-2.518599	-4.516154
H	5.304597	-3.845820	-4.465186
H	5.475851	-2.423737	-3.376485
C	-3.900897	0.130450	-2.837027
H	-3.162358	0.564121	-3.526990
C	-5.220749	-0.228834	-3.183833
H	-5.567851	-0.076760	-4.215534
C	-6.061695	-0.769067	-2.211784
H	-7.095778	-1.055407	-2.459306
C	-5.610878	-0.967714	-0.873843
C	-4.249458	-0.584886	-0.576430
C	-3.588112	-0.710786	0.729051
C	-4.466243	-1.270042	1.728066
C	-5.796610	-1.648920	1.465018
H	-6.397736	-2.068771	2.284812
C	-6.373231	-1.508516	0.194276
Cl	3.297027	2.109013	-1.489195
H	-2.508508	0.212266	-1.308335
Cl	-0.667357	1.271104	-2.302025

2b"H+			
-4274.8440066			
Ru	-0.064659	-0.057504	0.195316
Cl	4.988582	1.396142	-0.538816
Cl	5.004622	-2.964600	-3.807361
Cl	0.517255	6.541661	-2.100088
Cl	-0.450456	4.299155	2.764367
N	-1.359047	-1.667724	2.512381
N	0.830608	-1.598046	2.705702
N	0.856247	-1.609440	-0.911631
N	0.111253	1.387733	-1.391377
O	2.239103	0.470528	0.154412
O	-0.391598	1.698419	1.229912
O	-3.485663	-1.569303	-2.326272
C	-0.217437	-1.194151	1.920831
C	-1.108487	-2.425946	3.761769
H	-1.514426	-3.455564	3.673285
H	-1.628728	-1.936862	4.612590
C	0.420993	-2.391414	3.888565
H	0.772770	-1.899470	4.820103
H	0.882720	-3.400729	3.851366
C	-2.738386	-1.419713	2.184228
C	-3.492312	-2.429727	1.532737
C	-4.865443	-2.200499	1.308899
H	-5.455349	-2.976689	0.793743
C	-5.505752	-1.024460	1.738839
C	-4.734857	-0.064160	2.429806
H	-5.224637	0.850586	2.803009
C	-3.362905	-0.238628	2.678257
C	-2.876023	-3.741506	1.099476
H	-3.226719	-4.031076	0.087655
H	-3.171225	-4.567352	1.783344
H	-1.769390	-3.702054	1.086845
C	-6.977594	-0.790375	1.490697
H	-7.461835	-1.667977	1.019083
H	-7.134062	0.085173	0.824823
H	-7.515344	-0.571662	2.437249
C	-2.586699	0.795718	3.455727
H	-2.027667	0.341004	4.301203
H	-3.264738	1.561671	3.878878
H	-1.838649	1.310036	2.816686
C	2.224933	-1.265365	2.596251
C	3.130094	-2.217227	2.064657
C	4.507052	-1.905324	2.066149
H	5.217010	-2.643277	1.656603
C	5.001244	-0.696560	2.590023
C	4.070739	0.220873	3.129492
H	4.438012	1.161574	3.573564
C	2.687476	-0.043565	3.162802
C	2.664620	-3.559709	1.547630
H	3.303922	-3.911788	0.713608
H	1.615513	-3.528587	1.195540
H	2.727065	-4.336461	2.341635
C	6.479682	-0.383536	2.582718
H	6.730424	0.321409	1.760628
H	7.091743	-1.295316	2.436658
H	6.797738	0.097874	3.529906
C	1.732920	0.940174	3.799196
H	0.987737	1.335890	3.076137
H	2.281478	1.799317	4.230898
H	1.154409	0.470060	4.623019
C	-1.790748	-0.535796	-0.395917
H	-1.993292	-1.620200	-0.527891
C	-2.880597	0.261017	-0.928618
C	-3.784144	-0.314328	-1.899467
C	-4.870494	0.446642	-2.383242
H	-5.563630	0.027573	-3.124894
C	-5.076330	1.757691	-1.923271
H	-5.929002	2.333259	-2.317662
C	-4.213500	2.335556	-0.972954
H	-4.391027	3.357942	-0.607111
C	-3.130464	1.592902	-0.495700
H	-2.464865	2.007046	0.271817
C	-4.293831	-2.235742	-3.338471
H	-4.579322	-1.478349	-4.102664
C	-5.547695	-2.852767	-2.715828
H	-5.270137	-3.634836	-1.979398
H	-6.170670	-3.327143	-3.501432
H	-6.169525	-2.095322	-2.199017
C	-3.381139	-3.270797	-3.991329
H	-2.481976	-2.789899	-4.425260
H	-3.920085	-3.796875	-4.804720
H	-3.055248	-4.029636	-3.249848
C	0.251334	-2.728634	-1.352453
H	-0.778979	-2.892504	-1.008489
C	0.851804	-3.648329	-2.239678
H	0.280797	-4.533723	-2.555013
C	2.134764	-3.413815	-2.713989
H	2.624418	-4.095088	-3.424510
C	2.838154	-2.265156	-2.255645
C	2.171236	-1.390761	-1.319527
C	2.881585	-0.277354	-0.778556
C	4.182957	-0.003010	-1.210042
C	4.836527	-0.826346	-2.154161
H	5.858923	-0.585968	-2.476291
C	4.176782	-1.941733	-2.659589
C	0.291261	1.192137	-2.702872
H	0.281116	0.147687	-3.051512
C	0.480200	2.265204	-3.609877

C	-5.104297	0.499922	-3.232720			H	0.624511	2.049309	-4.678598
H	-5.294521	1.565871	-3.497324			C	0.482968	3.571227	-3.134895
C	-4.447292	-0.194451	-4.423988			H	0.632698	4.426622	-3.810800
H	-3.508180	0.320019	-4.708102			C	0.284509	3.818114	-1.745825
H	-5.130781	-0.188238	-5.297210			C	0.086862	2.672921	-0.895871
H	-4.202780	-1.247179	-4.174613			C	-0.154226	2.787225	0.517500
C	-6.408767	-0.170972	-2.793857			C	-0.158770	4.095217	1.055759
H	-6.221376	-1.232705	-2.534172			C	0.051918	5.234540	0.243537
H	-7.155641	-0.136531	-3.613735			H	0.038166	6.231099	0.707040
H	-6.850646	0.326120	-1.907177			C	0.266190	5.106742	-1.129333
H	1.212231	-2.651899	-0.892002			H	2.870316	1.019740	0.675968
Cl	-0.469591	-1.978439	-1.549772						

**2b"H+→2b”**				**2b”**				**2b”→3b**				**2b→2d”**			
**-4735.1870134**				**-4735.2002086**				**-4735.1907826**				**-4735.1646116**			
Ru	-0.052958	0.117995	0.166158	Ru	0.051060	-0.447976	-0.158643	Ru	-0.600778	-0.309453	-0.174537	Ru	0.07734800	0.22899900	-0.22030800
Cl	4.707870	1.600510	-2.921348	Cl	4.443608	1.240469	3.852679	Cl	4.478889	-2.764855	3.751408	Cl	-4.83785500	0.28333600	-0.10868500
Cl	5.718167	-3.706223	-2.409819	Cl	4.723172	5.285250	0.281059	Cl	7.739611	-0.293669	0.183623	Cl	-5.00024400	-4.45671300	2.54182300
Cl	-2.493941	5.674789	-3.376592	Cl	-1.897464	-2.982051	6.080254	Cl	-4.297497	-1.092248	5.711319	Cl	-2.05381900	4.47241200	4.92320200
Cl	-2.299333	4.318115	1.895561	Cl	-2.664369	-4.574434	0.928578	Cl	-5.571699	-0.864484	0.419482	Cl	0.03183700	5.26036300	-0.04404800
N	-0.708669	-1.394826	2.792205	N	-1.038085	-0.514464	-3.059193	N	-0.889289	0.757156	-3.068452	N	1.81594700	0.20827600	-2.79700900
N	1.300122	-0.488182	2.902682	N	0.808213	-1.711792	-2.935796	N	-0.888215	-1.450645	-3.054080	N	-0.34653500	0.23555500	-3.26539500
N	1.388688	-1.486208	-0.443278	N	1.680300	1.093488	-0.574781	N	2.558129	-0.567193	-0.480189	N	-0.61735100	-2.58793000	0.41079700
N	-0.549333	1.136376	-1.683913	N	-0.163775	-0.245172	1.981131	N	-0.746131	-0.290327	1.965446	N	-0.52547300	0.54494900	1.82616300
O	2.119237	0.931352	-1.605273	O	2.850132	-0.094625	1.924852	O	2.334011	-2.121177	1.885045	O	-1.93411800	-0.39765800	-0.54279400
O	-1.118553	1.729716	0.851461	O	-1.257996	-1.971755	0.260659	O	-2.598381	-0.389967	0.058726	O	0.21850400	2.23494100	-0.09366100
O	-2.473442	-2.906671	-1.982788	O	-1.725955	3.717577	0.107340	O	1.615273	3.536764	0.589209	O	3.40702700	-2.40159700	1.37475900
C	0.201880	-0.614249	2.095322	C	-0.047320	-0.944570	-2.185630	C	-0.793005	-0.352353	-2.240276	C	0.55588300	0.22999800	-2.22869300
C	-0.244340	-1.766491	4.149482	C	-0.895245	-1.048255	-4.432370	C	-1.080710	0.409861	-4.496442	C	1.77519200	0.03856500	-4.26874800
H	-0.283298	-2.868180	4.287275	H	-0.888222	-0.220870	-5.173944	H	-0.267754	0.845659	-5.117431	H	2.11200100	-0.98389300	-4.54552700
H	-0.913346	-1.316989	4.915562	H	-1.760431	-1.701460	-4.679858	H	-2.037941	0.837013	-4.865680	H	2.45379200	0.76673400	-4.75841600
C	1.179125	-1.202807	4.193906	C	0.429410	-1.809591	-4.365405	C	-1.074340	-1.122168	-4.485426	C	0.29858300	0.27153400	-4.59695200
H	1.346619	-0.497377	5.035042	H	0.340964	-2.875497	-4.663037	H	-2.021456	-1.568415	-4.857669	H	0.10797800	1.25571000	-5.08197500
H	1.958397	-1.992290	4.261539	H	1.227499	-1.358387	-4.994823	H	-0.247430	-1.560982	-5.083985	H	-0.13199000	-0.51299200	-5.25182900
C	-2.116970	-1.594075	2.553993	C	-2.312065	0.105974	-2.788512	C	-1.182037	2.129731	-2.734502	C	3.10361900	0.51043900	-2.22072400
C	-2.599656	-2.876292	2.179414	C	-2.527650	1.462960	-3.149587	C	-0.198202	3.133495	-2.929710	C	4.09779800	-0.50071100	-2.13402500
C	-3.990667	-3.058623	2.034218	C	-3.813025	2.013939	-2.968734	C	-0.547194	4.478507	-2.686298	C	5.37596200	-0.15140200	-1.65120300
H	-4.363579	-4.051061	1.728822	H	-3.975975	3.071853	-3.235599	H	0.222880	5.256176	-2.824850	H	6.14114800	-0.94204900	-1.57200600
C	-4.910639	-2.024548	2.278004	C	-4.888882	1.257013	-2.473561	C	-1.839985	4.856815	-2.285940	C	5.70600200	1.16355700	-1.27819600
C	-4.404746	-0.789600	2.733180	C	-4.662550	-0.108626	-2.206011	C	-2.814417	3.843946	-2.173037	C	4.72257900	2.15723300	-1.45045200
H	-5.109028	0.021094	2.984756	H	-5.503506	-0.737120	-1.867877	H	-3.848651	4.118155	-1.904982	H	4.97009500	3.20726000	-1.21973600
C	-3.028684	-0.554266	2.902540	C	-3.403888	-0.713072	-2.375671	C	-2.522935	2.489071	-2.408980	C	3.43403500	1.86703500	-1.93724500
C	-1.689316	-4.059166	1.928824	C	-1.436978	2.345749	-3.714347	C	1.215577	2.817829	-3.361695	C	3.84015700	-1.91944800	-2.58167600
H	-1.590000	-4.262196	0.840299	H	-0.967441	2.959730	-2.915489	H	1.898067	2.783693	-2.484678	H	4.50221500	-2.63049000	-2.04851600
H	-2.107474	-4.978234	2.388720	H	-1.852931	3.049471	-4.463745	H	1.602688	3.601872	-4.044087	H	4.05585100	-2.04111900	-3.66725700
H	-0.671018	-3.908236	2.334314	H	-0.628397	1.767141	-4.201230	H	1.295535	1.842214	-3.878299	H	2.79176400	-2.23691000	-2.42049600
C	-6.392819	-2.220912	2.060175	C	-6.244394	1.880115	-2.233193	C	-2.182695	6.297115	-1.981358	C	7.06467700	1.50478700	-0.70957400
H	-6.672019	-3.293839	2.060446	H	-6.375248	2.817494	-2.810655	H	-1.433595	6.997397	-2.402555	H	7.82367700	0.73896800	-0.96805200
H	-6.700405	-1.796848	1.079054	H	-6.372057	2.131072	-1.157104	H	-2.218284	6.467007	-0.882944	H	7.02348300	1.56954300	0.40014200
H	-6.993230	-1.705965	2.838203	H	-7.068240	1.188068	-2.504071	H	-3.179265	6.574438	-2.383229	H	7.42749300	2.48751200	-1.07581200
C	-2.551773	0.762814	3.460249	C	-3.243352	-2.195157	-2.145647	C	-3.618141	1.456046	-2.324617	C	2.46327800	2.99138000	-2.19449900
H	-1.867001	0.620347	4.323091	H	-2.797978	-2.703016	-3.027906	H	-3.647478	0.809273	-3.227149	H	2.00860200	2.91113900	-3.20429400
H	-3.407235	1.372972	3.809073	H	-4.225000	-2.668839	-1.950495	H	-4.609110	1.940526	-2.226714	H	2.97440300	3.97175200	-2.12921500
H	-2.000574	1.349672	2.695516	H	-2.580157	-2.403898	-1.280077	H	-3.475168	0.781319	-1.454751	H	1.63030800	2.98876600	-1.46295700
C	2.473836	0.346232	2.776888	C	1.919805	-2.567436	-2.583921	C	-0.943052	-2.863718	-2.753405	C	-1.77094500	0.49590100	-3.28020400
C	3.717252	-0.229388	2.418319	C	3.248802	-2.122467	-2.763787	C	0.224049	-3.653181	-2.894766	C	-2.67724900	-0.55354200	-3.57479300
C	4.861234	0.595542	2.424856	C	4.294774	-3.046301	-2.554968	C	0.110407	-5.046608	-2.735601	C	-4.04156600	-0.24002300	-3.72900100
H	5.827825	0.158954	2.120620	H	5.335381	-2.701608	-2.680712	H	1.019749	-5.664058	-2.829869	H	-4.74680300	-1.06073400	-3.94545800
C	4.809222	1.941832	2.826525	C	4.053324	-4.383310	-2.196653	C	-1.118543	-5.668475	-2.443455	C	-4.52942900	1.07303800	-3.60998600
C	3.567381	2.460135	3.241823	C	2.712636	-4.798458	-2.057153	C	-2.260786	-4.853387	-2.330561	C	-3.59895200	2.09624200	-3.34889400
H	3.502492	3.511101	3.568463	H	2.495877	-5.842860	-1.775840	H	-3.237062	-5.317928	-2.111705	H	-3.94993600	3.13942200	-3.27408500
C	2.390425	1.692946	3.222936	C	1.635040	-3.918915	-2.248240	C	-2.204148	-3.455543	-2.491712	C	-2.22236100	1.84081100	-3.19594200
C	3.873013	-1.699667	2.105130	C	3.586479	-0.694306	-3.120075	C	1.576539	-3.025764	-3.120639	C	-2.23910300	-1.99076700	-3.71517800
H	2.913088	-2.177304	1.834888	H	2.771919	-0.188380	-3.673937	H	1.555617	-2.210097	-3.872960	H	-2.59606200	-2.42607200	-4.67262300
H	4.287539	-2.248922	2.980103	H	4.508929	-0.642182	-3.733020	H	2.319190	-3.778429	-3.450984	H	-2.67302100	-2.61034900	-2.90156200
H	4.582241	-1.857493	1.268064	H	3.767365	-0.096602	-2.200982	H	1.936453	-2.580875	-2.168664	H	-1.14326800	-2.11354700	-3.65681000
C	6.037698	2.821351	2.784032	C	5.190088	-5.347698	-1.942417	C	-1.202074	-7.162402	-2.224003	C	-6.00720600	1.36667700	-3.73299000
H	6.044654	3.434837	1.856256	H	5.339912	-5.503539	-0.851445	H	-1.005049	-7.415849	-1.158843	H	-6.53381300	1.11458500	-2.78633000
H	6.973285	2.226327	2.795064	H	6.147695	-4.974312	-2.357784	H	-0.453222	-7.709802	-2.832317	H	-6.48018200	0.76477700	-4.53653600
H	6.065000	3.528318	3.638776	H	4.987199	-6.345270	-2.385001	H	-2.207420	-7.558887	-2.473031	H	-6.19864900	2.43793800	-3.94481500
C	1.076792	2.303079	3.640361	C	0.216394	-4.384693	-2.034501	C	-3.454977	-2.621444	-2.362762	C	-1.27040800	2.99027000	-2.97082100
H	0.419452	2.436242	2.755322	H	-0.241064	-3.854905	-1.171909	H	-3.380747	-1.914889	-1.510417	H	-0.77708700	2.93343200	-1.97957700
H	1.232135	3.298244	4.099795	H	0.182748	-5.471655	-1.826411	H	-4.342937	-3.262188	-2.198774	H	-1.80354400	3.95902200	-3.02849700
H	0.529372	1.671004	4.371722	H	-0.433511	-4.190921	-2.915438	H	-3.647802	-2.010811	-3.271757	H	-0.45935300	3.01092800	-3.73022300
C	-1.455985	-1.055787	-0.231416	C	-1.138870	1.034419	-0.321215	C	-0.187726	1.526382	-0.111886	C	1.77225600	-0.37903400	0.30000400
H	-1.225516	-2.141767	-0.172820	H	-0.790268	1.877387	-0.961036	H	0.722412	1.760371	-0.711479	H	2.14688200	-1.20311400	-0.34612600
C	-2.773398	-0.850752	-0.826402	C	-2.324560	1.434927	0.428239	C	-0.574535	2.625407	0.759924	C	2.57612100	-0.16569600	1.49856000
C	-3.308656	-1.864386	-1.701882	C	-2.634113	2.833178	0.609879	C	0.351735	3.682597	1.079518	C	3.44695600	-1.19223600	2.01320600
C	-4.599844	-1.718781	-2.249474	C	-3.795009	3.216427	1.314953	C	-0.061155	4.763647	1.887664	C	4.25615400	-0.93311100	3.14014300
H	-5.013847	-2.480904	-2.923927	H	-4.034365	4.277569	1.469763	H	0.636379	5.573388	2.142493	H	4.91029100	-1.71472800	3.55071700
C	-5.371823	-0.583063	-1.944624	C	-4.658690	2.239324	1.841111	C	-1.374551	4.812531	2.388204	C	4.23104200	0.32756900	3.76193300
H	-6.375274	-0.483214	-2.389141	H	-5.555745	2.561009	2.394761	H	-1.673824	5.662803	3.022231	H	4.87044900	0.50449500	4.64174400
C	-4.874144	0.412747	-1.087809	C	-4.384584	0.871628	1.668408	C	-2.296968	3.795347	2.086027	C	3.40900300	1.35228500	3.26276500
H	-5.484056	1.295688	-0.843355	H	-5.066893	0.108818	2.073468	H	-3.326689	3.841822	2.472147	H	3.40565000	2.34709100	3.73403200
C	-3.589472	0.275650	-0.545441	C	-3.229530	0.487030	0.976906	C	-1.891535	2.721497	1.284483	C	2.59843500	1.09706700	2.15048500
H	-3.198070	1.027975	0.150422	H	-3.009286	-0.574865	0.808833	H	-2.604112	1.930051	1.014365	H	1.98146400	1.89877500	1.72395700
C	-2.883759	-3.985634	-2.856876	C	-1.919117	5.147125	0.230631	C	2.648212	4.510301	0.877236	C	4.38012800	-3.43085200	1.68336500
H	-3.466348	-3.551068	-3.701093	H	-2.332043	5.360803	1.243202	H	2.529639	4.845247	1.933304	H	4.46118000	-3.52092100	2.79138500
C	-3.745770	-4.999023	-2.097579	C	-2.884529	5.657870	-0.843454	C	2.536422	5.712872	-0.064828	C	5.75042300	-3.08630000	1.09213400
H	-3.160601	-5.464945	-1.277649	H	-2.465116	5.476023	-1.854613	H	2.691386	5.393778	-1.116197	H	5.69407500	-3.05568300	-0.01504700
H	-4.087402	-5.805530	-2.778853	H	-3.049238	6.749143	-0.728302	H	3.306771	6.472024	0.183353	H	6.49837900	-3.85274800	1.38231000
H	-4.643025	-4.523227	-1.653656	H	-3.869650	5.153860	-0.783121	H	1.542199	6.197169	0.005342	H	6.11355400	-2.10052900	1.44396100
C	-1.596791	-4.593204	-3.411518	C	-0.529851	5.773501	0.127956	C	3.978188	3.771310	0.745023	C	3.81373600	-4.73661200	1.12917200
H	-1.006133	-3.832895	-3.959777	H	0.138460	5.386547	0.922168	H	4.024117	2.911332	1.441807	H	2.86889800	-5.01175800	1.63843600
H	-1.832210	-5.423428	-4.107885	H	-0.596511	6.875448	0.232623	H	4.821503	4.453806	0.974857	H	4.53705000	-5.56294500	1.28211100
H	-0.967460	-4.994301	-2.590512	H	-0.068645	5.543607	-0.854475	H	4.112660	3.386960	-0.287043	H	3.61080100	-4.64430800	0.04243200
C	1.090209	-2.706736	0.037152	C	1.465540	1.641613	-1.784626	C	2.714098	0.062159	-1.640741	C	0.08077500	-3.55040700	1.02163800
H	0.204411	-2.770696	0.676650	H	0.725504	1.132038	-2.409387	H	1.789969	0.289855	-2.196293	H	1.16255200	-3.57362900	0.81139300
C	1.839719	-3.878483	-0.202251	C	2.073824	2.814680	-2.277200	C	3.974705	0.444371	-2.172299	C	-0.52336200	-4.51372800	1.86755800
H	1.489384	-4.832424	0.219378	H	1.833977	3.158195	-3.294052	H	4.030792	0.946831	-3.149755	H	0.09503700	-5.28694900	2.34769400
C	3.011158	-3.792256	-0.934060	C	2.915530	3.523716	-1.444557	C	5.115295	0.185131	-1.429075	C	-1.90142300	-4.48257600	2.04497700
H	3.643931	-4.669673	-1.131650	H	3.375583	4.474875	-1.748315	H	6.115348	0.479803	-1.781583	H	-2.41827500	-5.23038600	2.66581000
C	3.424255	-2.520962	-1.414559	C	3.209646	3.003174	-0.155351	C	5.002600	-0.484771	-0.178593	C	-2.67194000	-3.44849400	1.44073800
C	2.570169	-1.367398	-1.173800	C	2.640771	1.714012	0.243015	C	3.673970	-0.889883	0.247889	C	-1.95864500	-2.47315300	0.64154500
C	2.955886	-0.068487	-1.683568	C	3.118660	1.117672	1.479449	C	3.505126	-1.654526	1.464557	C	-2.61324200	-1.28347600	0.10962500
C	4.213820	0.036243	-2.330162	C	3.944003	1.911791	2.321489	C	4.652932	-1.889055	2.251289	C	-4.00467800	-1.17105400	0.38590300
C	5.057085	-1.074047	-2.545593	C	4.410680	3.191605	1.972992	C	5.942765	-1.457404	1.862630	C	-4.72318900	-2.15757500	1.09639500
H	6.014033	-0.938768	-3.068452	H	5.062329	3.742910	2.664273	H	6.801631	-1.680334	2.510576	H	-5.79953600	-2.01461900	1.26805900
C	4.659424	-2.331613	-2.109324	C	4.066757	3.716942	0.737316	C	6.118210	-0.781494	0.662223	C	-4.07652600	-3.27727000	1.61672700
C	-0.343513	0.745862	-2.944302	C	0.219006	0.738683	2.801548	C	0.207231	-0.148316	2.890961	C	-0.80192400	-0.32681600	2.80483000
H	0.127806	-0.238239	-3.083018	H	0.692110	1.615964	2.336450	H	1.224719	0.044719	2.524859	H	-0.59096400	-1.38345400	2.59479100
C	-0.686005	1.559649	-4.051439	C	0.048464	0.667151	4.205851	C	-0.064155	-0.263953	4.278231	C	-1.32070500	0.07619000	4.06109800

H	-0.490324	1.192710	-5.069872	H	0.405053	1.498558	4.831253	H	0.764277	-0.156626	4.993682	H	-1.53613700	-0.69105200	4.81930200
C	-1.242166	2.815250	-3.836133	C	-0.535381	-0.458342	4.771383	C	-1.357091	-0.524529	4.711390	C	-1.55282500	1.42077800	4.31635800
H	-1.501085	3.480782	-4.673354	H	-0.658756	-0.558700	5.860087	H	-1.597339	-0.630282	5.779789	H	-1.96241300	1.76422700	5.27785200
C	-1.487672	3.254918	-2.505082	C	-1.004439	-1.503322	3.924637	C	-2.403437	-0.653854	3.751467	C	-1.23835400	2.38394000	3.31346500
C	-1.133494	2.352336	-1.442280	C	-0.816239	-1.335200	2.508085	C	-2.039759	-0.519148	2.367733	C	-0.71110600	1.88881500	2.07099300
C	-1.378780	2.647341	-0.055989	C	-1.341299	-2.263419	1.541276	C	-3.001244	-0.585237	1.306453	C	-0.30622400	2.75951400	1.00644900
C	-1.963290	3.900142	0.229729	C	-1.998724	-3.406821	2.051613	C	-4.347622	-0.807693	1.669484	C	-0.46679700	4.14805700	1.21016200
C	-2.287642	4.821141	-0.793853	C	-2.144961	-3.622731	3.440514	C	-4.727360	-0.970178	3.021734	C	-1.01152900	4.65706300	2.41150900
H	-2.729980	5.789584	-0.521450	H	-2.655137	-4.530841	3.790960	H	-5.784292	-1.151057	3.262342	H	-1.12757000	5.74363800	2.52794400
C	-2.060689	4.508899	-2.133140	C	-1.666322	-2.690679	4.360814	C	-3.779851	-0.895098	4.043119	C	-1.39000100	3.79771100	3.44281100
H	2.181115	1.773648	-0.832376	H	2.443203	-0.776683	1.262038	H	1.604942	-2.174122	1.174560	H	0.04928800	-2.83494100	-1.23498200
Cl	1.827874	2.835100	0.309759	Cl	1.768530	-2.236371	0.436967	Cl	-0.076331	-2.719199	0.272631	Cl	0.59778000	-3.55329400	-2.24338700

2d”				2d”→2d”bis				2d”bis				2d”bis→3b			
**-4735.206600**				**-4735.1926596**				**-4735.2254131**				**-4735.2116757**			
Ru	-0.257136	0.310571	0.314015	Ru	0.301601	-0.128163	-0.354061	Ru	0.481761	-0.375502	0.091676	Ru	-0.775449	-0.200854	0.300530
Cl	2.035482	2.170049	0.123469	Cl	-3.033247	1.833294	-1.140319	Cl	-3.458996	1.114923	-2.118392	Cl	5.259992	2.120093	0.340493
Cl	6.666148	0.962394	-2.455331	Cl	-6.869549	0.758469	2.547745	Cl	-7.331556	1.106048	1.686343	Cl	8.230156	-1.680616	-2.194187
Cl	-2.287727	5.823272	-3.501182	Cl	-0.332713	6.762918	0.669985	Cl	-1.521838	6.282288	-0.833111	Cl	1.567968	6.168499	-1.455696
Cl	-2.995162	4.353103	1.697213	Cl	1.396569	3.689143	-3.485610	Cl	0.029592	2.298099	-4.213988	Cl	-1.268164	4.227247	2.772751
N	-1.194996	-1.365546	2.738136	N	1.977725	-2.146175	-2.008449	N	2.095615	-2.694038	-1.131377	N	-2.577712	-1.874762	2.189783
N	0.826836	-0.556991	3.058046	N	-0.154047	-2.222993	-2.554117	N	-0.060949	-3.126282	-1.173068	N	-0.492627	-1.707235	2.878596
N	3.166242	-2.666726	-1.232474	N	-2.635513	-2.183372	2.051383	N	-2.690517	-1.026733	2.499288	N	3.152886	-2.235780	-1.368251
N	-0.426101	1.356043	-1.538031	N	-0.167579	1.546349	0.865264	N	-0.052591	1.559817	0.839283	N	0.140923	1.161910	-1.059586
O	1.428203	-0.829148	-0.255347	O	-1.723333	-0.713718	-0.068756	O	-1.828315	-0.410972	-0.049567	O	2.943769	0.159614	-0.130020
O	-1.509685	1.847522	0.857163	O	0.826444	1.386335	-1.591484	O	0.392042	0.710305	-1.666247	O	-1.035158	1.586273	1.307840
O	-1.644342	-2.909511	-2.327768	O	2.680053	-0.962206	3.198660	O	4.077929	0.471707	2.620767	O	-3.615832	-0.870837	-3.060666
C	-0.219640	-0.579221	2.172920	C	0.746785	-1.604801	-1.725678	C	0.860390	-2.171097	-0.814677	C	-1.355869	-1.335647	1.881691
C	-0.769076	-2.000837	4.005400	C	1.901968	-3.272732	-2.966701	C	2.038589	-4.080519	-1.640823	C	-2.559377	-2.697797	3.419922
H	-0.641909	-3.094920	3.861113	H	2.051682	-4.238102	-2.434728	H	2.469698	-4.786665	-0.896622	H	-2.750268	-3.766016	3.174653
H	-1.537047	-1.845520	4.791290	H	2.692930	-3.178519	-3.738162	H	2.629551	-4.174404	-2.575636	H	-3.357024	-2.367449	4.118200
C	0.559037	-1.297459	4.310082	C	0.485443	-3.134532	-3.527892	C	0.537355	-4.297042	-1.850574	C	-1.142161	-2.462873	3.968730
H	0.492003	-0.585794	5.164232	H	0.463177	-2.676913	-4.543777	H	0.246200	-4.302435	-2.925601	H	-1.136365	-1.866117	4.908463
H	1.382636	-2.007236	4.528115	H	-0.060442	-4.097612	-3.581245	H	0.164800	-5.240196	-1.400836	H	-0.594509	-3.406747	4.172061
C	-2.558810	-1.562506	2.322571	C	3.286368	-1.760388	-1.550365	C	3.385920	-2.066395	-1.105839	C	-3.836800	-1.662090	1.537120
C	-2.967916	-2.818281	1.797546	C	3.965116	-2.564155	-0.596307	C	4.312336	-2.415151	-0.088976	C	-4.355441	-2.665223	0.680257
C	-4.325997	-2.984204	1.454827	C	5.282708	-2.207072	-0.243395	C	5.610062	-1.868903	-0.152933	C	-5.643847	-2.476158	0.139167
H	-4.642748	-3.949862	1.025901	H	5.807539	-2.818756	0.509998	H	6.331513	-2.125587	0.641053	H	-6.055377	-3.250472	-0.530270
C	-5.282218	-1.971225	1.649037	C	5.945764	-1.108674	-0.821952	C	6.011085	-1.013820	-1.196547	C	-6.415392	-1.334299	0.427815
C	-4.855906	-0.770699	2.251884	C	5.259719	-0.366161	-1.803989	C	5.073466	-0.712394	-2.205832	C	-5.865742	-0.357597	1.284413
H	-5.595664	0.019033	2.467300	H	5.769592	0.478954	-2.296777	H	5.371575	-0.055607	-3.040351	H	-6.453148	0.545611	1.520800
C	-3.514193	-0.549202	2.615190	C	3.943232	-0.675822	-2.194050	C	3.764443	-1.226977	-2.189709	C	-4.587763	-0.498151	1.855262
C	-2.014947	-3.978173	1.639326	C	3.320288	-3.780969	0.025142	C	3.923225	-3.334139	1.046729	C	-3.541288	-3.890934	0.334084
H	-2.400635	-4.702222	0.895503	H	3.841216	-4.064279	0.960993	H	4.609109	-3.209266	1.907603	H	-3.994021	-4.439383	-0.515223
H	-1.911771	-4.537415	2.596637	H	3.383669	-4.661812	-0.652923	H	3.973882	-4.404925	0.746637	H	-3.481132	-4.603651	1.186487
H	-0.992861	-3.687986	1.317817	H	2.247459	-3.622590	0.267756	H	2.888285	-3.137620	1.396230	H	-2.498624	-3.621407	0.061158
C	-6.719676	-2.158447	1.220386	C	7.344039	-0.723425	-0.394017	C	7.400266	-0.417178	-1.234290	C	-7.791100	-1.141192	-0.171067
H	-7.008118	-3.229131	1.204706	H	7.889419	-1.581308	0.048932	H	8.085664	-0.924907	-0.526296	H	-8.129524	-2.041354	-0.721792
H	-6.877430	-1.760682	0.193361	H	7.314720	0.081372	0.373269	H	7.375781	0.660934	-0.963521	H	-7.800941	-0.286158	-0.881206
H	-7.421640	-1.619724	1.889587	H	7.941070	-0.336766	-1.245628	H	7.844845	-0.483107	-2.249452	H	-8.546565	-0.914917	0.611051
C	-3.125507	0.721156	3.332027	C	3.260803	0.115994	-3.281814	C	2.788484	-0.892999	-3.290327	C	-4.023926	0.558844	2.773425
H	-2.496703	0.512115	4.223505	H	2.842371	-0.547163	-4.069055	H	2.344581	-1.809161	-3.735490	H	-3.726173	0.131937	3.755251
H	-4.026482	1.267793	3.673017	H	3.969945	0.814665	-3.767263	H	3.284365	-0.326170	-4.102318	H	-4.767213	1.357257	2.964281
H	-2.547328	1.395441	2.667424	H	2.413611	0.707712	-2.876694	H	1.941745	-0.283396	-2.907053	H	-3.111833	1.026873	2.344236
C	2.012653	0.252745	3.024928	C	-1.564902	-1.986194	-2.721208	C	-1.498945	-3.066796	-1.203656	C	0.887853	-1.317127	3.020748
C	3.261897	-0.360971	2.754456	C	-2.481866	-2.935724	-2.200304	C	-2.237894	-3.765409	-0.217175	C	1.906981	-2.230298	2.641811
C	4.423756	0.435536	2.838564	C	-3.853554	-2.740554	-2.456140	C	-3.643508	-3.791443	-0.332769	C	3.248434	-1.848250	2.837747
H	5.398989	-0.030351	2.617395	H	-4.574419	-3.471057	-2.050793	H	-4.227587	-4.326697	0.435439	H	4.046330	-2.545578	2.531262
C	4.378778	1.793118	3.206547	C	-4.328782	-1.654111	-3.217228	C	-4.317096	-3.172541	-1.403115	C	3.599690	-0.613873	3.416409
C	3.121371	2.359767	3.500218	C	-3.384665	-0.751220	-3.743061	C	-3.544570	-2.515410	-2.382049	C	2.560613	0.236206	3.842390
H	3.063064	3.418895	3.803891	H	-3.731093	0.096992	-4.357260	H	-4.050243	-2.030753	-3.233677	H	2.812371	1.191658	4.332431
C	1.929585	1.616274	3.419008	C	-2.001419	-0.897677	-3.517833	C	-2.140788	-2.448045	-2.308433	C	1.202669	-0.092619	3.672768
C	3.368943	-1.832985	2.429446	C	-2.013239	-4.126157	-1.398579	C	-1.550309	-4.475991	0.926154	C	1.575462	-3.585099	2.060153
H	4.323255	-2.051892	1.910159	H	-2.877361	-4.698367	-1.005539	H	-2.292967	-4.871109	1.647911	H	2.499587	-4.163701	1.861672
H	2.532699	-2.203549	1.802139	H	-1.365279	-3.830763	-0.543017	H	-0.854060	-3.797427	1.463484	H	1.006536	-3.485475	1.110719
H	3.371432	-2.447485	3.357882	H	-1.416441	-4.831867	-2.018579	H	-0.949084	-5.343077	0.572888	H	0.950931	-4.191594	2.751880
C	5.637077	2.630583	3.269584	C	-5.810279	-1.450190	-3.445616	C	-5.826421	-3.195301	-1.501617	C	5.043329	-0.194690	3.564781
H	6.547170	2.000907	3.332863	H	-6.372308	-2.403284	-3.370995	H	-6.254363	-2.191992	-1.285599	H	5.295584	0.573164	2.801158
H	5.629093	3.314540	4.143490	H	-6.014696	-1.006066	-4.441468	H	-6.275293	-3.911003	-0.783865	H	5.738146	-1.047731	3.430910
H	5.739093	3.267878	2.363604	H	-6.238191	-0.757085	-2.687828	H	-6.164302	-3.474736	-2.521711	H	5.237734	0.259184	4.559097
C	0.601330	2.263845	3.733951	C	-1.022910	0.085428	-4.115996	C	-1.349773	-1.715801	-3.364018	C	0.125626	0.829674	4.191941
H	-0.046159	2.345610	2.834127	H	-0.497832	0.676796	-3.336602	H	-0.906457	-0.782379	-2.954969	H	-0.453255	1.284082	3.360650
H	0.745858	3.282931	4.142674	H	-1.539456	0.793737	-4.792690	H	-1.993019	-1.442297	-4.222663	H	0.566946	1.651924	4.787947
H	0.020475	1.680048	4.478940	H	-0.231454	-0.424603	-4.705771	H	-0.506898	-2.326191	-3.752222	H	-0.599088	0.294555	4.842292
C	-1.342624	-1.052829	-0.349194	C	1.857370	-0.384582	0.645309	C	2.268836	-0.081735	0.603321	C	-2.345109	-0.323151	-0.686115
H	-0.940746	-2.075468	-0.173312	H	1.960850	-1.456648	0.919200	H	2.689898	-0.851349	1.281195	H	-2.626351	-1.355344	-0.980514
C	-2.490605	-0.996397	-1.239747	C	2.749461	0.518029	1.361866	C	3.134369	1.092400	0.528057	C	-3.169361	0.694890	-1.329525
C	-2.680032	-2.045140	-2.209845	C	3.238143	0.154422	2.666804	C	4.100169	1.338690	1.571052	C	-3.861309	0.366463	-2.552236
C	-3.853776	-2.077565	-2.992992	C	4.191581	0.965423	3.318178	C	4.965563	2.451041	1.488243	C	-4.696474	1.323222	-3.169119
H	-4.014824	-2.880261	-3.726309	H	4.573124	0.692271	4.312158	H	5.699414	2.652373	2.281014	H	-5.223714	1.086867	-4.103629
C	-4.818763	-1.064539	-2.851971	C	4.652784	2.142150	2.700846	C	4.891787	3.324589	0.388848	C	-4.854489	2.597802	-2.598224
H	-5.725167	-1.099600	-3.478174	H	5.393983	2.766649	3.225625	H	5.573557	4.189572	0.345711	H	-5.508707	3.330503	-3.097982
C	-4.637567	-0.014941	-1.932283	C	4.179443	2.523128	1.432813	C	3.962934	3.102129	-0.641965	C	-4.188227	2.938890	-1.408529
H	-5.400641	0.771294	-1.826348	H	4.550291	3.440934	0.951361	H	3.912374	3.782543	-1.505794	H	-4.318622	3.935951	-0.960918
C	-3.481317	0.014328	-1.141680	C	3.235343	1.717142	0.781095	C	3.097231	2.003948	-0.559444	C	-3.351816	1.997434	-0.796293
H	-3.338255	0.798868	-0.385399	H	2.883152	1.978100	-0.226358	H	2.382925	1.798827	-1.366937	H	-2.838728	2.236510	0.144211
C	-1.729528	-4.122894	-3.118252	C	3.119663	-1.515535	4.460348	C	4.950278	0.640967	3.760526	C	-4.217278	-1.314622	-4.299184
H	-2.273336	-3.892735	-4.063682	H	3.305919	-0.677359	5.171975	H	5.033851	1.729904	3.983247	H	-4.228976	-0.457630	-5.011751
C	-2.470906	-5.206762	-2.332272	C	4.404529	-2.326700	4.268248	C	6.339532	0.062268	3.473490	C	-5.645351	-1.812996	-4.058677
H	-1.903934	-5.447503	-1.409973	H	4.216536	-3.181980	3.587246	H	6.267140	-1.027677	3.279199	H	-5.636706	-2.666667	-3.350307
H	-2.564213	-6.130221	-2.940368	H	4.759904	-2.726121	5.240675	H	7.010807	0.216201	4.343701	H	-6.103085	-2.152017	-5.010965
H	-3.491609	-4.878169	-2.048593	H	5.215959	-1.708458	3.833917	H	6.808419	0.538077	2.589261	H	-6.290792	-1.020021	-3.631234
C	-0.285545	-4.503638	-3.437341	C	1.948871	-2.355311	4.969335	C	4.250999	-0.047339	4.931997	C	-3.291890	-2.398620	-4.849122
H	0.199821	-3.717847	-4.051271	H	1.066642	-1.714576	5.168307	H	3.254611	0.401804	5.113333	H	-2.269973	-1.999890	-5.004697
H	-0.257220	-5.456611	-4.004469	H	2.225580	-2.875620	5.909074	H	4.857180	0.052374	5.855431	H	-3.675019	-2.776746	-5.818735
H	0.285368	-4.620588	-2.490399	H	1.653856	-3.103234	4.205060	H	4.107792	-1.126710	4.720059	H	-3.225707	-3.250151	-4.141158
C	3.951495	-3.629356	-1.723321	C	-3.030054	-2.986594	3.044766	C	-3.014658	-1.386504	3.748775	C	3.113282	-3.437515	-1.962331
H	3.552984	-4.649294	-1.592370	H	-2.325318	-3.798752	3.285552	H	-2.226940	-1.898646	4.320819	H	2.128746	-3.925515	-1.998815
C	5.189648	-3.317330	-2.323557	C	-4.255560	-2.759030	3.703926	C	-4.299279	-1.092957	4.248655	C	4.305066	-3.980809	-2.488234
H	5.817572	-4.120897	-2.734742	H	-4.573849	-3.429591	4.514862	H	-4.567464	-1.391114	5.272052	H	4.281354	-4.965742	-2.975855
C	5.599681	-1.988080	-2.362469	C	-5.037403	-1.674972	3.312177	C	-5.204306	-0.420899	3.429701	C	5.492382	-3.258274	-2.381303
H	6.567146	-1.705138	-2.804633	H	-5.996398	-1.459945	3.808039	H	-6.213321	-0.170391	3.791996	H	6.434332	-3.661543	-2.784729
C	4.781154	-0.956422	-1.817054	C	-4.615859	-0.811286	2.260875	C	-4.853449	-0.034577	2.103518	C	5.523279	-1.978735	-1.750652
C	3.507421	-1.344991	-1.253353	C	-3.358014	-1.110365	1.611810	C	-3.521038	-0.369168	1.640961	C	4.271669	-1.474921	-1.227510
C	2.561667	-0.389735	-0.684402	C	-2.811035	-0.332114	0.510747	C	-2.994923	-0.058481	0.309529	C	4.079080	-0.182311	-0.544946
C	3.040076	0.953414	-0.651668	C	-3.600191	0.802238	0.147922	C	-3.945559	0.651237	-0.509448	C	5.318210	0.559616	-0.448413
C	4.279617	1.353807	-1.188679	C	-4.825942	1.117603	0.772193	C	-5.241617	0.990786	-0.077486	C	6.544912	0.091235	-0.949667
H	4.571400	2.412484	-1.138694	H	-5.383904	2.001867	0.432454	H	-5.902417	1.536806	-0.766363	H	7.438575	0.721944	-0.833388
C	5.136511	0.424269	-1.778071	C	-5.336658	0.329871	1.805162	C	-5.702662	0.657280	1.201523	C	6.663767	-1.153223	-1.590328
C	0.061109	1.021496	-2.739611	C	-0.629867	1.568729	2.122716	C	-0.218499	1.944754	2.109888	C	0.700187	0.899271	-2.245722
H	0.597112	0.061801	-2.797087	H	-0.755817	0.590641	2.612359	H	0.003258	1.181270	2.873504	H	0.630221	-0.147059	-2.582863
C	-0.101381	1.854391	-3.874894	C	-0.924977	2.782372	2.793103	C	-0.657055	3.251253	2.443789	C	1.343343	1.903161	-3.009447

H	0.318463	1.534443	-4.840190	H	-1.300568	2.746438	3.826567	H	-0.782375	3.523415	3.502726	H	1.790198	1.638324	-3.979307
C	-0.777842	3.062679	-3.754460	C	-0.737311	3.996614	2.143174	C	-0.919795	4.169706	1.434179	C	1.404597	3.204875	-2.526152
H	-0.910298	3.735791	-4.614668	H	-0.961417	4.953991	2.636712	H	-1.261417	5.190918	1.660743	H	1.899784	4.006382	-3.094601
C	-1.319395	3.442970	-2.491037	C	-0.239326	4.007573	0.806703	C	-0.739829	3.791457	0.070666	C	0.816125	3.517235	-1.266973
C	-1.131594	2.528782	-1.396511	C	0.044046	2.733629	0.205903	C	-0.293210	2.445518	-0.181918	C	0.182096	2.440223	-0.556024
C	-1.662964	2.765403	-0.079373	C	0.563104	2.596948	-1.124304	C	-0.053982	1.934833	-1.509484	C	-0.457136	2.618216	0.721221
C	-2.346802	3.989115	0.113788	C	0.774223	3.788957	-1.853518	C	-0.274516	2.840564	-2.576530	C	-0.452456	3.928890	1.251243
C	-2.525145	4.915005	-0.939670	C	0.492282	5.055035	-1.290911	C	-0.726539	4.159443	-2.355407	C	0.173366	5.003633	0.577756
H	-3.067714	5.850369	-0.743196	H	0.669698	5.957741	-1.892221	H	-0.892591	4.820101	-3.218027	H	0.158073	6.003260	1.034170
C	-2.030872	4.649412	-2.217747	C	-0.000960	5.167278	0.010491	C	-0.957874	4.630148	-1.061189	C	0.797376	4.806435	-0.653723
H	2.228477	-3.101279	-0.685562	H	-1.621087	-2.432813	1.640907	H	-1.690393	-1.236302	2.176983	H	2.258627	-1.829615	-0.976860
Cl	1.227932	-4.301990	0.076573	Cl	0.020339	-3.220102	1.516456	Cl	0.273470	-1.597557	2.255637	Cl	0.037215	-2.213151	-0.846338

2b→2d'			
**-4735.1822894**			
Ru	-0.09828800	-0.09430600	0.22202900
Cl	0.11533000	4.02107700	2.73945000
Cl	2.89822200	6.69824300	-1.03217300
O	-0.20892700	1.91338800	0.71332500
N	0.69893400	2.06719100	-2.04691400
N	0.43081900	-1.23665900	2.88359600
N	-1.74855200	-0.91759900	2.66640700
C	-4.86486500	1.06968600	2.11349600
H	-5.21680600	2.10531800	2.25397100
C	-3.07671700	-0.56752500	2.23997800
C	-3.53365100	0.76443900	2.44212200
C	-5.28921700	-1.22001800	1.47703300
H	-5.97756700	-2.00140200	1.11309100
C	-3.96144200	-1.57996800	1.78939800
C	-3.53492800	-3.02344100	1.64098100
H	-4.05915500	-3.67502000	2.37347600
H	-2.44647500	-3.15522900	1.78969900
H	-3.79094100	-3.40667300	0.63113900
C	-5.75923000	0.09684100	1.61886000
C	-2.60956900	1.81924000	2.98797900
H	-3.13612200	2.78453600	3.11652900
H	-1.74206400	1.98374800	2.31490900
H	-2.19226900	1.52937300	3.97677800
C	-7.17532300	0.47330000	1.24650200
H	-7.64521600	1.11129800	2.02397100
H	-7.81545800	-0.42008700	1.10204000
H	-7.19155900	1.05564700	0.29946300
C	-0.54207000	-0.80089700	2.01000800
C	-0.09047100	-1.50672100	4.23748600
H	0.26134900	-0.72328900	4.94599100
H	0.27861700	-2.48468200	4.60886500
C	-1.61085900	-1.47665900	4.02809400
H	-2.07312600	-2.48856000	4.07835100
H	-2.13815900	-0.83409100	4.76327100
C	3.77779700	-2.80281300	2.27070300
H	4.19536400	-3.80129300	2.05767900
C	2.38057700	-2.67187400	2.42188100
C	2.70200100	-0.25632600	2.83296200
C	6.13472600	-1.84400500	2.15955700
H	6.43910500	-1.31955300	1.22798600
H	6.44079200	-2.90512400	2.06867500
H	6.71363900	-1.38710400	2.98984200
C	1.84855600	-1.38069900	2.66637900
C	4.08540900	-0.44229000	2.67409200
H	4.74767400	0.43317700	2.77585800
C	4.64581900	-1.70233900	2.37733400
C	1.51445900	-3.91051500	2.36506800
H	1.81100500	-4.56594600	1.52128200
H	0.44173400	-3.66947500	2.24359900
H	1.62478900	-4.51327400	3.29378000
C	2.14435700	1.09975000	3.18022200
H	1.36684400	1.42217300	2.46034300
H	2.94098600	1.86768900	3.18827100
H	1.66961500	1.10307000	4.18589500
C	0.97265400	3.05233900	-1.12864000
C	2.25785300	4.28113900	-2.84847400
H	2.85638100	5.15628800	-3.14226100
C	1.99620300	3.24873900	-3.72902300
H	2.38011400	3.25446300	-4.76007200
C	0.48044400	2.92788300	0.24581600
C	1.45540900	5.18804500	0.70537800
H	1.61639000	5.99945400	1.42838200
C	1.96876700	5.27187700	-0.58026500
C	1.19580700	2.17049300	-3.27997700
H	0.94614100	1.35524300	-3.97935500
C	0.73162100	4.04384000	1.09503200
C	1.74517700	4.22352300	-1.52216400
O	-3.87011400	-1.34173900	-2.05052400
C	-1.88760900	-0.33709100	-0.38097900
H	-2.25258100	-1.38324900	-0.46823400
C	-2.79876500	1.97229200	-0.89205800
H	-2.00875700	2.38171000	-0.24949600
C	-4.73032500	2.25596400	-2.32379300
H	-5.46711100	2.90439900	-2.82543000
C	-3.86915900	0.00986700	-1.88861800
C	-2.83151600	0.56001900	-1.04592600
C	-3.72946400	2.81463600	-1.50932300
H	-3.67243500	3.90409100	-1.36170600
C	-4.80384200	0.86562100	-2.51168500
H	-5.59612500	0.45542100	-3.15287300
C	-4.70583600	-1.98119800	-3.04334500
H	-4.80773100	-1.29112000	-3.91150000
C	-3.95059300	-3.22971600	-3.49777500
H	-2.95121400	-2.95765300	-3.88951200
H	-4.51537700	-3.75384000	-4.29568400
H	-3.81324000	-3.93443500	-2.65140000
C	-6.08822300	-2.29808100	-2.46421900
H	-5.99623700	-3.00678800	-1.61515000
H	-6.73386500	-2.76636100	-3.23591800
H	-6.59855100	-1.38603100	-2.09577800
H	-0.25979600	0.42842500	-2.57023500
Cl	-0.55905100	-0.54722700	-3.44656700
N	0.44287700	-1.98916400	-0.44746000
O	1.94785900	0.17860900	-0.19023300
Cl	4.86340700	0.52022900	-1.01336400
Cl	4.37611700	-4.65203400	-2.64458900
C	2.52049600	-0.85876100	-0.73868500

2d'			
**-4735.2153304**			
Ru	0.345265	0.063008	-0.258317
Cl	-4.664931	0.895657	-1.208155
Cl	-2.938417	6.032176	-1.773550
Cl	-4.932210	-5.663687	-0.512308
Cl	-0.670960	-3.863082	2.273430
N	1.526617	-0.520228	2.493688
N	-0.534604	0.272119	2.649334
N	0.266878	2.107618	-0.461106
N	-1.887552	-1.925801	-2.367809
O	-1.785927	0.367023	-0.421371
O	-1.976536	-2.075098	-0.122354
O	4.573838	0.922782	-1.720828
C	0.482852	0.010460	1.757261
C	1.143960	-0.817692	3.890776
H	1.922276	-0.461444	4.597084
H	1.037135	-1.916705	4.026707
C	-0.193827	-0.085675	4.042466
H	-0.988612	-0.723286	4.481595
H	-0.120807	0.832798	4.668043
C	2.925506	-0.668686	2.173983
C	3.784371	0.442110	2.408295
C	5.174732	0.270600	2.267192
H	5.835510	1.134188	2.453822
C	5.742249	-0.969958	1.917531
C	4.868885	-2.051647	1.700492
H	5.285911	-3.034315	1.422718
C	3.470750	-1.934885	1.832305
C	3.230487	1.788166	2.808701
H	4.045600	2.513419	2.999343
H	2.608447	1.728781	3.726916
H	2.575085	2.210333	2.018716
C	7.237605	-1.137828	1.766303
H	7.793051	-0.280107	2.195921
H	7.525044	-1.218777	0.695286
H	7.595669	-2.062983	2.264286
C	2.597765	-3.143877	1.629964
H	2.333099	-3.614595	2.602551
H	3.123591	-3.912522	1.030111
H	1.647448	-2.883103	1.126789
C	-1.717631	1.087523	2.497330
C	-1.601327	2.497666	2.625723
C	-2.775172	3.277746	2.594321
H	-2.681965	4.373761	2.674510
C	-4.052537	2.701002	2.475391
C	-4.134810	1.298009	2.396438
H	-5.124651	0.820066	2.307178
C	-2.993328	0.474387	2.406284
C	-0.265257	3.171703	2.832841
H	-0.374762	4.273571	2.824455
H	0.464262	2.895821	2.046928
H	0.190486	2.892249	3.808169
C	-5.301342	3.550575	2.414147
H	-5.765078	3.492114	1.405650
H	-5.085158	4.617155	2.623005
H	-6.066745	3.204563	3.140702
C	-3.154761	-1.015747	2.259903
H	-3.170557	-1.269154	1.179367
H	-4.109175	-1.362520	2.704291
H	-2.321578	-1.586773	2.711545
C	2.231326	-0.007069	-0.537458
H	2.868372	0.818205	-0.149590
C	3.009305	-0.849767	-1.439483
C	4.239528	-0.365799	-2.013841
C	4.999794	-1.197852	-2.864761
H	5.940854	-0.842164	-3.306809
C	4.552965	-2.495502	-3.166174
H	5.160866	-3.128867	-3.832812
C	3.344171	-2.980970	-2.635373
H	2.994710	-3.995262	-2.883559
C	2.584752	-2.157017	-1.797690
H	1.632409	-2.507474	-1.373249
C	5.697823	1.571797	-2.356845
H	5.764518	1.208462	-3.408024
C	6.999072	1.257915	-1.612178
H	6.937408	1.619416	-0.565109
H	7.858921	1.757439	-2.104790
H	7.203748	0.169086	-1.587105
C	5.370409	3.064527	-2.376222
H	4.417703	3.248562	-2.911158
H	6.176318	3.630369	-2.886523
H	5.273613	3.456068	-1.342113
C	1.295593	2.974327	-0.463525
H	2.292544	2.556227	-0.269970
C	1.131707	4.351202	-0.741433
H	2.017846	5.003208	-0.726919
C	-0.126661	4.855274	-1.037924
H	-0.284869	5.919056	-1.268866
C	-1.242541	3.969518	-1.059847
C	-1.004148	2.580616	-0.751725
C	-2.062255	1.591255	-0.742960
C	-3.352118	2.058191	-1.117473
C	-3.605618	3.407815	-1.427807
H	-4.622293	3.716846	-1.709292
C	-2.576680	4.351195	-1.386338
C	-2.649379	-1.791172	-3.455653
H	-2.312780	-1.023093	-4.168826
C	-3.789654	-2.600052	-3.627590

2d'→3c			
**-4735.1821947**			
Ru	0.703379	0.251518	-0.413655
Cl	-3.519980	2.851780	-1.861317
Cl	-0.206370	7.114834	-1.149716
Cl	-7.392951	-3.880808	-1.114357
Cl	-3.279853	-4.048072	2.457263
N	1.048933	-1.217423	2.230252
N	-0.515401	0.351033	2.318307
N	1.268134	2.189411	-0.154617
N	-2.936937	-1.375311	-1.912614
O	-1.153473	1.267935	-0.785530
O	-1.868424	-2.244822	0.428977
O	5.079622	-0.236953	-1.612842
C	0.505415	-0.154833	1.540457
C	0.397353	-1.423945	3.542249
H	1.071284	-1.077918	4.361129
H	0.181858	-2.497925	3.703912
C	-0.874782	-0.586137	3.408095
H	-1.737012	-1.212635	3.091545
H	-1.139771	-0.030226	4.328664
C	2.211450	-2.013061	1.945376
C	3.505331	-1.480079	2.197516
C	4.626606	-2.305568	1.987778
H	5.631441	-1.888172	2.169703
C	4.500907	-3.646109	1.575270
C	3.203060	-4.157446	1.389097
H	3.078544	-5.209646	1.082298
C	2.046450	-3.372624	1.568665
C	3.695920	-0.083540	2.740704
H	4.733613	0.269312	2.578577
H	3.506459	-0.048919	3.836768
H	3.000717	0.641615	2.277253
C	5.717457	-4.505719	1.316632
H	6.617777	-4.110889	1.829411
H	5.944312	-4.546628	0.228468
H	5.559010	-5.551137	1.652904
C	0.682128	-3.992652	1.388610
H	0.321292	-4.443107	2.340987
H	0.722349	-4.819997	0.651766
H	-0.094289	-3.269261	1.065339
C	-1.126587	1.654386	2.293606
C	-0.397476	2.756489	2.823109
C	-1.012531	4.021496	2.852369
H	-0.443002	4.877097	3.253092
C	-2.333158	4.219987	2.401094
C	-3.047586	3.096473	1.948616
H	-4.089114	3.222415	1.609163
C	-2.478030	1.806519	1.895412
C	0.987599	2.581257	3.400779
H	1.337115	3.517383	3.878547
H	1.729433	2.305317	2.624682
H	1.013261	1.777192	4.166911
C	-2.946335	5.602065	2.393702
H	-2.517950	6.217587	1.572418
H	-2.745255	6.144553	3.341391
H	-4.043899	5.563352	2.245056
C	-3.312856	0.635627	1.444205
H	-4.022476	0.948212	0.653532
H	-3.914022	0.230748	2.288903
H	-2.684389	-0.183399	1.048531
C	2.497637	-0.399861	-0.613881
H	3.312240	0.297371	-0.316103
C	3.082535	-1.512180	-1.350939
C	4.434820	-1.412674	-1.851356
C	5.005531	-2.480667	-2.577292
H	6.030632	-2.412131	-2.966548
C	4.259470	-3.646720	-2.820096
H	4.720962	-4.466448	-3.394714
C	2.944322	-3.772284	-2.339376
H	2.365971	-4.692842	-2.529164
C	2.375175	-2.716059	-1.620740
H	1.348645	-2.797968	-1.240513
C	6.405671	0.018899	-2.130880
H	6.480733	-0.432093	-3.146887
C	7.471623	-0.586126	-1.211968
H	7.413755	-0.128289	-0.202758
H	8.486432	-0.398768	-1.620021
H	7.343686	-1.681264	-1.100836
C	6.524028	1.536588	-2.261972
H	5.728221	1.936603	-2.920989
H	7.508543	1.809974	-2.693041
H	6.432660	2.024158	-1.269256
C	2.483396	2.646490	0.210115
H	3.214809	1.895485	0.534583
C	2.822969	4.017483	0.171201
H	3.837561	4.317278	0.473585
C	1.887619	4.958899	-0.238047
H	2.125977	6.031792	-0.279566
C	0.585081	4.522287	-0.615153
C	0.305859	3.110626	-0.552968
C	-0.975558	2.547429	-0.906455
C	-1.953712	3.468238	-1.371944
C	-1.704453	4.853063	-1.445379
H	-2.497049	5.525108	-1.803616
C	-0.466759	5.374573	-1.063855
C	-3.355239	-0.860252	-3.074577
H	-2.628316	-0.229254	-3.605364
C	-4.655720	-1.162248	-3.530972

3c			
**-3339.0358833**			
Ru	0.248736	-0.458410	0.626616
Cl	3.831624	1.909423	3.401028
Cl	4.746913	4.540097	-1.280210
N	-1.446380	-2.309535	-1.226424
N	0.708727	-2.788492	-1.111110
N	1.104340	0.814996	-0.770776
O	1.681175	0.480666	1.796049
O	-2.986800	2.619223	-0.347777
C	-0.258885	-1.921512	-0.654697
C	-1.303322	-3.477200	-2.125446
H	-1.583527	-3.200716	-3.166342
H	-1.986371	-4.293102	-1.807817
C	0.183630	-3.853117	-1.986866
H	0.333787	-4.852185	-1.521335
H	0.720804	-3.856023	-2.959442
C	-2.778221	-1.846675	-0.938000
C	-3.405813	-0.914709	-1.801510
C	-4.735812	-0.532763	-1.527849
H	-5.224859	0.196234	-2.195727
C	-5.452833	-1.055779	-0.438174
C	-4.816550	-2.020685	0.369970
H	-5.371526	-2.468581	1.211179
C	-3.496599	-2.445222	0.136334
C	-2.702412	-0.313797	-2.996951
H	-2.426181	0.743915	-2.796621
H	-3.363105	-0.313058	-3.888444
H	-1.775720	-0.860674	-3.256519
C	-6.858206	-0.593868	-0.126511
H	-7.343358	-0.125750	-1.006702
H	-6.848475	0.160673	0.690661
H	-7.498857	-1.432423	0.216368
C	-2.871496	-3.505710	1.006603
H	-2.465312	-4.348558	0.406967
H	-3.614764	-3.924852	1.712681
H	-2.022671	-3.090404	1.595785
C	2.113819	-2.749511	-0.784271
C	3.017660	-2.083255	-1.656041
C	4.382214	-2.040655	-1.307989
H	5.080458	-1.503098	-1.971887
C	4.878854	-2.675203	-0.154283
C	3.973487	-3.414864	0.631308
H	4.348403	-3.968089	1.508573
C	2.598372	-3.487179	0.334570
C	2.584416	-1.499393	-2.981467
H	3.007863	-0.487138	-3.138334
H	1.485723	-1.423982	-3.069760
H	2.950236	-2.134883	-3.818114
C	6.336187	-2.567269	0.232048
H	6.495438	-1.711812	0.924928
H	6.985198	-2.398502	-0.651109
H	6.690134	-3.478756	0.755312
C	1.693843	-4.345778	1.181525
H	1.043129	-3.717111	1.829856
H	2.288836	-5.016156	1.832121
H	1.021410	-4.977987	0.564921
C	-1.312243	0.556936	0.394943
H	-1.369723	1.121787	-0.561547
C	-2.385916	0.977416	1.293200
C	-3.246678	2.062731	0.871482
C	-4.283230	2.516145	1.716049
H	-4.945319	3.333030	1.397588
C	-4.481338	1.930656	2.977443
H	-5.293225	2.305648	3.621797
C	-3.648564	0.886072	3.414315
H	-3.793717	0.434014	4.407595
C	-2.621085	0.428559	2.583089
H	-1.949859	-0.375220	2.921110
C	-3.616915	3.854990	-0.760079
H	-3.787718	4.480094	0.145899
C	-4.951384	3.576157	-1.458885
H	-4.786468	2.976741	-2.378056
H	-5.441951	4.527837	-1.750936
H	-5.648751	3.016201	-0.804541
C	-2.611875	4.568240	-1.664112
H	-1.659659	4.749163	-1.127456
H	-3.018681	5.544814	-1.997285
H	-2.392751	3.958935	-2.565396
C	0.729226	1.021752	-2.044731
H	-0.096454	0.399640	-2.414770
C	1.354344	1.980919	-2.877752
H	1.004330	2.093379	-3.914943
C	2.398061	2.755927	-2.385550
H	2.909069	3.502751	-3.011219
C	2.808511	2.588358	-1.031186
C	2.111826	1.608187	-0.242983
C	2.389399	1.374420	1.148134
C	3.429158	2.147452	1.721004
C	4.144157	3.105087	0.962598
H	4.942074	3.683163	1.449926
C	3.843382	3.327124	-0.380741
Cl	-0.193306	-1.829212	2.543338

C	3.86243500	-0.90263400	-1.19332900	H	-4.412813	-2.486933	-4.525636	H	-5.005758	-0.739073	-4.483012	
C	4.41432500	-2.06637400	-1.77256400	C	-4.097962	-3.528574	-2.639569	C	-5.470802	-1.996492	-2.768500	
C	3.64947800	-3.22432300	-1.91496700	H	-4.977983	-4.183092	-2.733089	H	-6.487204	-2.252329	-3.106055	
C	2.28943900	-3.26496100	-1.48278400	C	-3.290189	-3.658288	-1.473207	C	-5.015668	-2.540303	-1.531570	
C	1.75006600	-2.06495000	-0.90038500	C	-2.133240	-2.795903	-1.346814	C	-3.680655	-2.187668	-1.109226	
C	1.42797800	-4.39461200	-1.58459200	C	-1.217375	-2.807805	-0.205384	C	-3.038115	-2.627791	0.135463	
C	0.12645800	-4.29684600	-1.10827000	C	-1.604098	-3.759569	0.796090	C	-3.900352	-3.472227	0.921905	
C	-0.33035200	-3.08542900	-0.54078300	C	-2.715702	-4.620517	0.689253	C	-5.201001	-3.842245	0.528673	
H	5.45997600	-2.05383300	-2.11108200	H	-2.925887	-5.321115	1.509659	H	-5.790645	-4.495129	1.188705	
H	1.81194800	-5.32162300	-2.03554800	C	-3.553402	-4.580904	-0.422338	C	-5.761950	-3.392891	-0.673534	
H	-0.56481100	-5.15096500	-1.16456600	Cl	0.183178	0.096683	-2.825211	Cl	0.092928	-0.764538	-2.530205	
H	-1.35826700	-2.99346300	-0.16587700	H	-1.040275	-1.250066	-2.330034	H	-1.953409	-1.165805	-1.601155	

3c→4c	4c	4c→5b	2b'H+
**-3799.8051594**	**-3799.8498625**	**-3799.8345064**	**-4274.8328261**
Ru -0.843410 -0.119006 0.621480	Ru 0.616047 -0.661336 -0.275674	Ru -0.686415 -0.341209 0.300333	Ru 0.141023 -0.058640 -0.168491
Cl 2.351425 -2.937191 3.417428	Cl -2.915013 -1.878202 -3.173387	Cl 4.284613 3.643468 2.446018	Cl -4.519074 2.139853 -0.429490
Cl 4.446958 -4.720411 -1.277322	Cl -7.410390 -0.809205 -0.315336	Cl 8.452851 1.476187 -0.325109	Cl -5.641120 -1.528107 3.431542
N -1.655635 2.641015 -0.438957	N 3.498563 0.401758 -0.396472	N -3.006325 -2.272512 0.469156	Cl -0.196959 6.771907 1.551522
N -3.390345 1.289393 -0.310483	N 3.311406 -1.772840 -0.056233	N -1.001260 -3.213034 0.413071	Cl 0.860808 4.115398 -3.097859
N -0.059930 -2.126148 -1.038139	N -2.698728 -0.674544 1.816848	N 3.524076 0.254417 -1.318110	N 1.248782 -1.913834 -2.355057
O 0.299112 -1.632745 1.665703	O -1.471691 -1.278201 -0.603828	O 2.579368 1.886935 0.615579	N -0.944597 -2.065758 -2.283114
O 3.306961 0.838691 -0.926125	O -0.714967 3.376798 1.251284	O -3.646523 2.936105 -1.112237	N -0.976108 -1.238808 1.092056
C -2.020778 1.337912 -0.164868	C 2.586072 -0.601847 -0.165530	C -1.663672 -1.998209 0.331579	N 0.144601 1.572253 1.253052
C -2.789072 3.494322 -0.854642	C 4.897404 -0.075847 -0.362151	C -3.281692 -3.722956 0.540167	O -1.816665 0.779815 -0.479693
H -2.707683 3.748385 -1.937179	H 5.405300 0.277680 0.565389	H -3.724574 -4.080160 -0.418764	O 0.769453 1.675468 -1.336840
H -2.782492 4.449035 -0.289707	H 5.467731 0.321275 -1.226554	H -4.010236 -3.941420 1.347305	O 3.288561 -1.966775 2.522293
C -4.001188 2.616911 -0.537391	C 4.734392 -1.601274 -0.400957	C -1.893568 -4.318051 0.804650	C 0.127468 -1.453934 -1.697562
H -4.539392 2.951496 0.377054	H 4.942211 -2.020846 -1.411496	H -1.747515 -4.577621 1.877956	C 0.943110 -2.822605 -3.483256
H -4.737888 2.568465 -1.365323	H 5.385095 -2.129273 0.326433	H -1.684678 -5.226028 0.202218	H 1.524719 -3.763234 -3.395172
C -0.362845 3.265610 -0.501340	C 3.297857 1.824240 -0.416290	C -4.146250 -1.406962 0.348623	H 1.230968 -2.344318 -4.445532
C 0.347295 3.257656 -1.730323	C 3.261334 2.529719 0.814664	C -4.686750 -1.149122 -0.938370	C -0.569211 -3.031232 -3.344944
C 1.529837 4.017554 -1.828812	C 3.203393 3.937464 0.778549	C -5.897249 -0.433666 -1.020735	H -1.132453 -2.811435 -4.275088
H 2.081265 4.016278 -2.784139	H 3.185826 4.490619 1.733191	H -6.328871 -0.241729 -2.017613	H -0.837394 -4.062082 -3.028655
C 2.012485 4.788332 -0.754230	C 3.187007 4.652655 -0.434962	C -6.573199 0.027102 0.127642	C 2.623133 -1.531881 -2.183721
C 1.277256 4.781282 0.448554	C 3.225972 3.918959 -1.637273	C -5.990932 -0.223212 1.385686	C 3.504530 -2.405173 -1.489281
H 1.634887 5.380528 1.302815	H 3.217260 4.459085 -2.599253	H -6.494743 0.142386 2.296296	C 4.861583 -2.043818 -1.384161
C 0.087005 4.045541 0.598705	C 3.292915 2.511827 -1.658666	C -4.787686 -0.994239 1.526145	H 5.544519 -2.712429 -0.834002
C -0.142152 2.447794 -2.909000	C 3.274514 1.791899 2.134033	C -3.968235 -1.599610 -2.189841	C 5.373603 -0.866333 -1.962203
H 0.499585 2.608825 -3.797193	H 3.250055 2.499935 2.985520	H -2.975570 -1.108138 -2.280610	C 4.485985 -0.055080 -2.698386
H -1.183895 2.707335 -3.193261	H 4.182813 1.162356 2.249208	H -4.556633 -1.356136 -3.095835	H 4.874153 0.848513 -3.198372
H -0.141893 1.359579 -2.681857	H 2.405165 1.105329 2.228552	H -3.779339 -2.694186 -2.194143	C 3.123148 -0.375345 -2.841002
C 3.276489 5.609935 -0.876490	C 3.119577 6.163950 -0.456639	C -7.905394 0.735358 0.014227	C 3.033599 -3.716854 -0.902217
H 3.714576 5.543537 -1.892168	H 3.352404 6.599854 0.535779	H -7.981684 1.324180 -0.922599	H 3.512714 -3.906416 0.078515
H 4.047287 5.271985 -0.150977	H 2.105948 6.517313 -0.747186	H -8.080804 1.420138 0.868518	H 3.308261 -4.567760 -1.563868
H 3.083911 6.682435 -0.659678	H 3.830501 6.592676 -1.193553	H -8.744409 0.004574 0.005392	H 1.936269 -3.744613 -0.760270
C -0.691577 4.099736 1.889024	C 3.375435 1.766010 -2.968105	C -4.201404 -1.211566 2.891434	C 6.823267 -0.477060 -1.793662
H -1.688142 4.572792 1.745459	H 4.357336 1.256361 -3.085846	H -4.123012 -2.300802 3.100027	H 7.470569 -1.362228 -1.633345
H -0.150740 4.692167 2.652537	H 3.258961 2.458422 -3.824880	H -4.828982 -0.763912 3.686566	H 6.949279 0.187397 -0.910694
H -0.880803 3.081444 2.291553	H 2.595386 0.977176 -3.034750	H -3.172536 -0.799989 2.977180	H 7.204896 0.076319 -2.675191
C -4.277567 0.157323 -0.374018	C 2.815631 -3.069162 0.333035	C 0.399740 -3.467178 0.200581	C 2.258588 0.469534 -3.752588
C -4.396816 -0.530634 -1.608872	C 2.826416 -3.402689 1.716607	C 0.833540 -3.771597 -1.119892	H 1.174362 0.291682 -3.599848
C -5.330812 -1.576533 -1.710828	C 2.360596 -4.671774 2.104910	C 2.191049 -4.080881 -1.324314	H 2.460951 0.236144 -4.820833
H -5.416437 -2.122748 -2.665713	H 2.353725 -4.925940 3.178363	H 2.529586 -4.322034 -2.346492	H 2.481900 1.551658 -3.640323
C -6.152999 -1.945031 -0.627682	C 1.911034 -5.622508 1.166515	C 3.119457 -4.114262 -0.263074	C -2.359769 -1.888123 -2.069138
C -6.033839 -1.215979 0.570265	C 1.988100 -5.289226 -0.197929	C 2.644552 -3.863019 1.037484	C -3.075435 -2.844791 -1.308869
H -6.675322 -1.481346 1.427575	H 1.686768 -6.034499 -0.953346	H 3.344526 -3.920697 1.887947	C -4.476648 -2.704361 -1.210105
C -5.116481 -0.156699 0.723526	C 2.457276 -4.035509 -0.645447	C 1.291981 -3.560170 1.302790	H -5.040009 -3.433675 -0.604603
C -3.546190 -0.141280 -2.795763	C 3.350870 -2.434993 2.751127	C -0.137222 -3.788909 -2.276478	C -5.173000 -1.675987 -1.869032
H -3.706444 -0.833445 -3.646143	H 3.357929 -2.900522 3.756340	H -0.497124 -2.762905 -2.499238	C -4.429643 -0.768197 -2.653856
H -2.465384 -0.139705 -2.540701	H 2.716528 -1.526340 2.799414	H -1.028488 -4.414935 -2.059003	H -4.956875 0.038979 -3.187963
H -3.779867 0.885178 -3.155330	H 4.388776 -2.110115 2.522613	H 0.343996 -4.190182 -3.190153	C -3.032852 -0.853602 -2.774552
C -7.110092 -3.110607 -0.737936	C 1.359683 -6.957478 1.615248	C 4.580856 -4.409623 -0.516033	C -2.398945 -4.026306 -0.649974
H -6.615846 -4.056913 -0.425038	H 0.272297 -6.882326 1.838471	H 5.069404 -4.854928 0.373998	H -2.861155 -4.253145 0.331429
H -7.463621 -3.255735 -1.779223	H 1.856246 -7.316631 2.540026	H 5.137757 -3.478106 -0.761122	H -1.317168 -3.857645 -0.486360
H -7.997372 -2.976582 -0.086264	H 1.478854 -7.734868 0.833526	H 4.716902 -5.104733 -1.369869	H -2.508254 -4.943418 -1.270275
C -5.049946 0.610362 2.022588	C 2.633688 -3.787521 -2.124437	C 0.822780 -3.415225 2.730857	C -6.672735 -1.535146 -1.753045
H -4.001274 0.804941 2.329154	H 2.266981 -2.786640 -2.432068	H 0.128758 -2.560237 2.860713	H -6.945236 -0.538917 -1.344300
H -5.549705 0.048515 2.836037	H 2.087960 -4.547739 -2.717375	H 1.682137 -3.260952 3.412479	H -7.110695 -2.308318 -1.092093
H -5.567762 1.592863 1.942922	H 3.707054 -3.861519 -2.410402	H 0.300001 -4.337401 3.070624	H -7.163017 -1.617332 -2.746473
C 0.791445 0.705007 0.268114	C 0.272353 1.148816 -0.046195	C -1.979672 0.934416 -0.071636	C -2.278861 0.147567 -3.616889
H 0.969203 0.820689 -0.828009	H 0.813581 1.685138 0.758664	H -3.016725 0.689050 -0.367191	H -1.758079 0.874697 -2.959094
C 1.988324 0.913456 1.062028	C -0.784002 1.949600 -0.656205	C -1.615966 2.338600 -0.025899	H -2.969114 0.717042 -4.268569
C 3.280520 1.015966 0.424010	C -1.255922 3.133159 0.023940	C -2.492861 3.364470 -0.531971	H -1.518365 -0.334815 -4.266354
C 4.432637 1.250105 1.204862	C -2.256596 3.935507 -0.564658	C -2.110754 4.720226 -0.431928	C 1.802807 -0.740383 0.513376
H 5.421393 1.338045 0.734501	H -2.623318 4.837775 -0.056249	H -2.778058 5.517744 -0.786568	H 1.866301 -1.835428 0.693066
C 4.329603 1.360970 2.602170	C -2.805021 3.581677 -1.810001	C -0.863092 5.069208 0.114564	C 2.955758 -0.078565 1.107514
H 5.244024 1.538332 3.191558	H -3.587446 4.221051 -2.250485	H -0.587502 6.134752 0.174239	H 3.734360 -0.745275 2.128387
C 3.086181 1.232408 3.245646	C -2.368891 2.428037 -2.483758	C 0.026548 4.082384 0.576395	C 4.860826 -0.103057 2.689363
H 3.015424 1.298438 4.341983	H -2.801364 2.151894 -3.457586	H 1.013328 4.337685 0.988768	H 5.456219 -0.593571 3.471193
C 1.938732 1.001395 2.480933	C -1.379397 1.623912 -1.905750	C -0.359679 2.741988 0.510371	C 5.235266 1.178655 2.256234
H 0.955418 0.879386 2.958729	H -1.017768 0.720156 -2.419144	H 0.347146 1.986500 0.895194	H 6.116659 1.659671 2.709583
C 4.561876 0.759065 -1.645394	C -1.113859 4.524466 2.033800	C -4.599521 3.878427 -1.657563	C 4.501818 1.845056 1.256135
H 5.302383 0.237057 -0.997603	H -2.218783 4.645292 1.944808	H -4.038235 4.719402 -2.124963	H 4.809751 2.843714 0.911274
C 5.076649 2.156478 -2.002178	C -0.410438 5.793038 1.542092	C -5.511560 4.412258 -0.549103	C 3.379617 1.219437 0.706234
H 4.360508 2.669901 -2.675740	H 0.688878 5.676920 1.631043	H -6.078517 3.578259 -0.086876	H 2.815072 1.717876 -0.092001
H 6.054447 2.085807 -2.522055	H -0.723027 6.669055 2.147334	H -6.236576 5.145029 -0.960021	C 3.974884 -2.724849 3.561340
H 5.211830 2.787692 -1.101641	H -0.646429 6.007130 0.480729	H -4.929673 4.915514 0.248880	H 4.292056 -2.011014 4.354492
C 4.294077 -0.105752 -2.875723	C -0.774008 4.181981 3.483669	C -5.356849 3.132173 -2.753876	C 5.194729 -3.448543 2.987426
H 3.958672 -1.119011 -2.579279	H -1.294878 3.256892 3.800864	H -4.659550 2.769440 -3.535156	H 4.882884 -4.200364 2.233023
H 5.217446 -0.206625 -3.481692	H -1.080212 5.007721 4.157638	H -6.102438 3.800565 -3.230492	H 5.738500 -3.981033 3.794213
H 3.508662 0.353678 -3.510800	H 0.317312 4.021025 3.600234	H -5.887220 2.256179 -2.328240	H 5.904698 -2.747032 2.505834
C -0.206289 -2.234366 -2.369032	C -3.196431 -0.357489 3.018882	C 3.851617 -0.588400 -2.307694	C 2.935168 -3.676946 4.146260
H -1.161461 -1.874743 -2.782222	H -2.462036 -0.257527 3.831890	H 3.014812 -1.096430 -2.807025	H 2.069151 -3.117109 4.551977
C 0.787910 -2.831459 -3.174192	C -4.585974 -0.184903 3.179076	C 5.215541 -0.766746 -2.626724	H 3.381429 -4.268604 4.970497
H 0.645589 -2.891429 -4.263063	H -4.992933 0.079991 4.165111	H 5.494211 -1.451447 -3.440243	H 2.571362 -4.385193 3.373280
C 1.909971 -3.382559 -2.561290	C -5.419296 -0.372297 2.077771	C 6.186212 -0.068649 -1.908904	C -0.551064 -2.264592 1.851536
H 2.682628 -3.898515 -3.152088	H -6.510540 -0.260351 2.171398	H 7.255279 -0.191378 -2.142773	H 0.494015 -2.575992 1.720261
C 2.076514 -3.310573 -1.147863	C -4.889052 -0.721632 0.803270	C 5.826125 0.823234 -0.855587	C -1.384152 -2.903802 2.800204
C 1.060300 -2.602851 -0.395376	C -3.449766 -0.855547 0.690678	C 4.414251 0.966403 -0.575071	H -0.973940 -3.736128 3.390480
C 1.137416 -2.383592 1.041360	C -2.742031 -1.190204 -0.541383	C 3.825587 1.831526 0.458339	C -2.692115 -2.472320 2.975539
C 2.223144 -3.061877 1.685136	C -3.626763 -1.434435 -1.649162	C 4.836909 2.559929 1.191804	H -3.361001 -2.942844 3.711565
C 3.205521 -3.775034 0.971388	C -5.028478 -1.319388 -1.559807	C 6.214475 2.436022 0.939469	C -3.184734 -1.393411 2.187500
H 4.036032 -4.234638 1.526228	H -5.632867 -1.509416 -2.458449	H 6.917735 3.026037 1.545488	C -2.285691 -0.795278 1.229090
C 3.156089 -3.882145 -0.422402	C -5.660494 -0.960177 -0.365118	C 6.716828 1.586514 -0.060483	C -2.676153 0.298961 0.371373
Cl -1.465292 0.687030 2.720422	Cl 0.852255 -0.823454 -2.634338	Cl -0.612509 -0.186220 2.631066	C -3.998217 0.789508 0.547698
H -1.186074 -2.234242 -0.347570	H -1.638834 -0.831554 1.752241	H 2.513189 0.402809 -1.072840	C -4.888644 0.226208 1.484575
Cl -2.455128 -2.368200 0.497641	Cl 0.284528 -1.085534 2.113661	Cl 0.377395 -0.375488 -1.818912	H -5.900782 0.643655 1.582889
			C -4.499907 -0.850588 2.284429
			C -0.074623 1.495993 2.570083
			H -0.114465 0.483126 2.999374
			C -0.260486 2.641119 3.383871

			H -0.442906 2.511562 4.460513<br>C -0.231309 3.904851 2.808644<br>H -0.392719 4.813160 3.407542<br>C 0.011242 4.030898 1.410998<br>C 0.213384 2.817155 0.668197<br>C 0.496984 2.875006 -0.724327<br>C 0.533571 4.103178 -1.379188<br>C 0.306880 5.310929 -0.673593<br>H 0.333403 6.268355 -1.211662<br>C 0.060487 5.274716 0.696000<br>H 0.922273 1.771785 -2.306981

2b'H+→2b'			
-4735.1873824			
Ru	0.30054800	-0.18673900	-0.26965700
Cl	-4.71336800	0.65717700	-1.17016700
Cl	-5.17445200	-1.34113400	3.87288400
Cl	-3.46353000	6.12586400	0.13389600
Cl	-0.91172300	3.73013800	-4.03364600
N	1.97637700	-2.54887200	-1.34610300
N	-0.20281200	-2.89438800	-1.53231000
N	-0.57294900	-0.90790100	1.40913800
N	-0.35562500	1.96922700	0.72597600
O	-1.76914600	0.02190900	-0.79323800
O	0.36530400	2.03687300	-1.80258400
O	3.53012800	1.33568500	2.50355400
C	0.73778500	-2.01643200	-1.04498100
C	1.87110500	-3.85029800	-2.04020800
H	2.14304500	-4.67729200	-1.34261400
H	2.56339900	-3.89125600	-2.90378900
C	0.40166100	-3.87817600	-2.45583200
H	0.26020200	-3.53315500	-3.50412900
H	-0.07545900	-4.87118700	-2.33888300
C	3.29337200	-2.16664500	-0.90379100
C	3.71750000	-2.50572600	0.41150300
C	5.02454100	-2.16390200	0.81385000
H	5.34197900	-2.41024100	1.84146100
C	5.93893400	-1.54922600	-0.05974200
C	5.52126500	-1.31641800	-1.38391200
H	6.23109100	-0.87474000	-2.10321500
C	4.22439600	-1.62687900	-1.83646100
C	2.85023300	-3.30530100	1.35464500
H	3.07845900	-3.06678500	2.41327100
H	3.03840200	-4.39495400	1.22817100
H	1.77228100	-3.14209300	1.18053300
C	7.32619000	-1.15636000	0.39551800
H	7.59955600	-1.64454100	1.35251800
H	7.39615700	-0.05681000	0.54717600
H	8.09443500	-1.42431800	-0.35942800
C	3.88558200	-1.43757100	-3.29396400
H	3.99644500	-2.39706200	-3.84940200
H	4.57939200	-0.71599400	-3.76913200
H	2.84506300	-1.08091000	-3.44364200
C	-1.60078800	-3.05881600	-1.20866300
C	-1.92516000	-3.82852300	-0.05460300
C	-3.27839000	-4.08214600	0.23413300
H	-3.52645400	-4.66756000	1.13559600
C	-4.31591900	-3.62476500	-0.60240100
C	-3.95759400	-2.92257800	-1.76564600
H	-4.75014900	-2.56960200	-2.44587800
C	-2.61608100	-2.63821400	-2.10436000
C	-0.85314100	-4.42487300	0.82851800
H	-1.30038600	-5.08604600	1.59637300
H	-0.26772800	-3.64802300	1.36008600
H	-0.12629800	-5.02922600	0.24515600
C	-5.76254300	-3.88538900	-0.24813500
H	-6.44669500	-3.57323600	-1.06196500
H	-6.05193300	-3.32862800	0.66959200
H	-5.94401700	-4.96146900	-0.04043300
C	-2.31451600	-1.93914100	-3.40481600
H	-3.14699100	-1.26308000	-3.68211500
H	-2.20595600	-2.67947400	-4.22997800
H	-1.38375900	-1.33943000	-3.35964200
C	2.00079200	0.10755100	0.53554600
H	2.21080900	-0.31647400	1.54472800
C	3.01381300	1.09990700	0.19123200
C	3.82403000	1.70116200	1.22105700
C	4.81851900	2.64345400	0.88189900
H	5.44198000	3.10878900	1.65783700
C	5.01346600	3.00778900	-0.46218500
H	5.79094600	3.74983200	-0.70696600
C	4.23263100	2.43733900	-1.48304400
H	4.39292200	2.72199900	-2.53415700
C	3.24906600	1.49888700	-1.15205800
H	2.63963000	1.03212400	-1.94117500
C	4.29193900	1.84495500	3.62199800
H	4.50249400	2.92484600	3.44544400
C	5.60808900	1.07666900	3.77608100
H	5.40580600	0.00636900	3.98759400
H	6.20429600	1.49106000	4.61523200
H	6.22339300	1.13451100	2.85629700
C	3.38389000	1.72022900	4.84432500
H	2.44771500	2.29488400	4.69869300
H	3.89678300	2.10979600	5.74701100
H	3.11799400	0.65837100	5.02675000
C	0.03891800	-1.39461100	2.50735200
H	1.13092900	-1.47584400	2.46240700
C	-0.67046300	-1.76925000	3.67257600
H	-0.10413300	-2.15897300	4.53173400
C	-2.05219200	-1.64031500	3.72075600
H	-2.62919800	-1.92023900	4.61443200
C	-2.74053400	-1.12556500	2.58438400
C	-1.95737300	-0.77099900	1.42641500
C	-2.54032900	-0.24071800	0.21598100
C	-3.94760600	-0.03266800	0.24025500
C	-4.73239200	-0.36843400	1.36129400
H	-5.81765300	-0.19710500	1.32740600
C	-4.14777400	-0.91615500	2.50568400
C	-0.51774000	2.05820100	2.04548600
H	-0.03629100	1.27509900	2.65343300
C	-1.28024500	3.07929600	2.67053300

2b'			
-4735.2186817			
Ru	0.569132	-0.342380	-0.537991
Cl	-4.550268	-0.923173	-1.120801
Cl	-4.442690	-1.258632	4.311003
Cl	-5.222848	4.882733	0.719584
Cl	-4.279621	2.475701	-4.047899
N	2.844910	-2.360000	-0.846037
N	0.852963	-3.301809	-0.671114
N	-0.118830	-0.655622	1.427678
N	-0.857872	2.144592	-0.205074
O	-1.524450	-0.698686	-0.828862
O	-1.772361	1.636541	-2.878747
O	3.477900	2.039056	2.015324
C	1.502657	-2.087950	-0.654618
C	3.135529	-3.808165	-0.902223
H	3.684921	-4.123814	0.013837
H	3.781713	-4.041169	-1.773501
C	1.742189	-4.433631	-0.999645
H	1.509292	-4.814417	-2.019751
H	1.586842	-5.269850	-0.287881
C	4.001208	-1.501504	-0.845217
C	4.708614	-1.287964	0.367128
C	5.904058	-0.541988	0.329467
H	6.448122	-0.366173	1.272878
C	6.427088	-0.030231	-0.871329
C	5.735772	-0.317074	-2.065870
H	6.147864	0.038246	-3.025273
C	4.541709	-1.061789	-2.086449
C	4.242310	-1.867644	1.684491
H	4.369811	-1.134091	2.506615
H	4.840206	-2.764316	1.960827
H	3.178508	-2.171951	1.657566
C	7.686441	0.806456	-0.887180
H	8.305045	0.636984	0.017158
H	7.439597	1.890374	-0.922649
H	8.310471	0.589402	-1.778414
C	3.888474	-1.409066	-3.400436
H	3.784085	-2.508461	-3.527970
H	4.487326	-1.028453	-4.250688
H	2.866704	-0.980434	-3.465970
C	-0.542180	-3.620659	-0.496837
C	-0.977661	-4.076552	0.773768
C	-2.319778	-4.485505	0.917624
H	-2.670179	-4.816640	1.909560
C	-3.216272	-4.484185	-0.166069
C	-2.733979	-4.073400	-1.425314
H	-3.417649	-4.074221	-2.290188
C	-1.409812	-3.641563	-1.622007
C	-0.033039	-4.185959	1.950004
H	-0.578909	-4.088691	2.909279
H	0.758794	-3.413144	1.924128
H	0.473248	-5.177657	1.968752
C	-4.663189	-4.887062	0.002825
H	-5.329923	-4.015940	-0.176109
H	-4.869636	-5.271467	1.021381
H	-4.956088	-5.670605	-0.727882
C	-0.957061	-3.193697	-2.988391
H	-0.924330	-2.084792	-3.044123
H	-1.649400	-3.558020	-3.772314
H	0.063946	-3.549419	-3.235872
C	2.120379	0.560332	0.087296
H	2.629314	0.171670	0.995420
C	2.635727	1.897181	-0.216508
C	3.382103	2.626293	0.783591
C	3.920674	3.895744	0.485943
H	4.489930	4.456610	1.239793
C	3.723764	4.468003	-0.783198
H	4.149174	5.462681	-0.993925
C	2.985525	3.787881	-1.766494
H	2.826485	4.239974	-2.757608
C	2.444058	2.530822	-1.474679
H	1.864266	1.982979	-2.231844
C	4.131212	2.714961	3.115521
H	3.864842	3.796094	3.069474
C	5.652358	2.552067	3.038836
H	5.931138	1.481850	3.127838
H	6.142180	3.107046	3.865566
H	6.059288	2.932943	2.081354
C	3.538619	2.121252	4.392824
H	2.438182	2.248992	4.415278
H	3.967418	2.621793	5.284603
H	3.766088	1.037207	4.463626
C	0.613993	-0.655203	2.555835
H	1.696462	-0.512171	2.438418
C	0.038794	-0.798594	3.840311
H	0.697289	-0.787521	4.721736
C	-1.336046	-0.945603	3.972318
H	-1.815116	-1.053879	4.956849
C	-2.149431	-0.953593	2.803269
C	-1.489324	-0.810290	1.528805
C	-2.201281	-0.810744	0.272130
C	-3.616357	-0.927552	0.356170
C	-4.281825	-1.062746	1.591952
H	-5.377253	-1.153106	1.604290
C	-3.568344	-1.084906	2.792304
C	-0.441269	2.503935	1.007496
H	0.542654	2.119996	1.318816
C	-1.186619	3.318541	1.898064

2b'→3c			
-4735.2151938			
Ru	-0.642543	0.432382	-0.530588
Cl	4.441198	1.149982	-1.085300
Cl	4.224693	2.012523	4.284588
Cl	5.752879	-4.775701	0.794372
Cl	4.583720	-2.501857	-4.000789
N	-3.182013	2.089211	-0.921252
N	-1.324277	3.285645	-0.798399
N	-0.018239	0.966220	1.406114
N	1.262297	-2.247442	-0.103240
O	1.426247	0.785921	-0.807098
O	2.064342	-1.700211	-2.726811
O	-3.205246	-2.130719	2.226084
C	-1.818048	2.001275	-0.732328
C	-3.656109	3.485065	-1.025915
H	-4.240706	3.759864	-0.118305
H	-4.327711	3.603066	-1.901077
C	-2.352461	4.282436	-1.155041
H	-2.176909	4.657945	-2.188580
H	-2.304361	5.153703	-0.470207
C	-4.186170	1.060197	-0.969379
C	-4.896820	0.715680	0.208971
C	-5.926704	-0.243285	0.118895
H	-6.469052	-0.525278	1.037139
C	-6.289309	-0.836286	-1.102923
C	-5.611041	-0.417517	-2.266440
H	-5.904638	-0.839510	-3.242298
C	-4.575611	0.534023	-2.233861
C	-4.610338	1.363216	1.545747
H	-4.531546	0.597107	2.344200
H	-5.431773	2.053508	1.838127
H	-3.669488	1.945482	1.536445
C	-7.364918	-1.896168	-1.175571
H	-8.025656	-1.875010	-0.285520
H	-6.913782	-2.911136	-1.231140
H	-7.997937	-1.774747	-2.078726
C	-3.925592	0.992584	-3.515938
H	-3.955778	2.098477	-3.621203
H	-4.440728	0.557623	-4.394613
H	-2.856685	0.692070	-3.552061
C	0.037168	3.745949	-0.679491
C	0.454244	4.333333	0.542969
C	1.770122	4.832562	0.633629
H	2.107772	5.266063	1.589976
C	2.658541	4.790322	-0.455972
C	2.189102	4.257119	-1.673323
H	2.862392	4.231940	-2.546057
C	0.889452	3.739127	-1.818208
C	-0.480089	4.485656	1.722693
H	0.062269	4.356934	2.680704
H	-1.309334	3.753376	1.698648
H	-0.930655	5.503445	1.744267
C	4.082083	5.284543	-0.337297
H	4.795430	4.441764	-0.463598
H	4.276946	5.752004	0.648203
H	4.321876	6.028993	-1.125964
C	0.443633	3.187947	-3.148695
H	0.487502	2.077569	-3.156019
H	1.092173	3.561410	-3.965091
H	-0.604877	3.459676	-3.388469
C	-2.029839	-0.635388	0.199220
H	-2.509943	-0.280197	1.136156
C	-2.404011	-2.033425	-0.028051
C	-3.055373	-2.776328	1.028793
C	-3.465116	-4.108957	0.814826
H	-3.964279	-4.678215	1.610821
C	-3.229965	-4.733581	-0.422524
H	-3.553043	-5.777575	-0.565614
C	-2.583415	-4.039769	-1.459046
H	-2.392745	-4.530469	-2.425885
C	-2.173440	-2.717957	-1.252600
H	-1.665305	-2.161481	-2.053497
C	-3.738256	-2.816990	3.383027
H	-3.359165	-3.864572	3.373964
C	-5.270391	-2.819759	3.363869
H	-5.659578	-1.782251	3.425035
H	-5.666379	-3.386604	4.231874
H	-5.669811	-3.283789	2.440424
C	-3.165986	-2.102112	4.606628
H	-2.058231	-2.111784	4.588949
H	-3.505948	-2.600435	5.537187
H	-3.505145	-1.045758	4.638300
C	-0.776396	1.076947	2.511689
H	-1.855330	0.914217	2.386916
C	-0.233853	1.367928	3.784925
H	-0.912316	1.438111	4.648262
C	1.134670	1.560906	3.929275
H	1.587881	1.785803	4.906263
C	1.972783	1.468601	2.781967
C	1.345905	1.166860	1.520218
C	2.081696	1.052288	0.287109
C	3.484986	1.246304	0.372444
C	4.122666	1.537221	1.598481
H	5.212570	1.678700	1.614821
C	3.387914	1.648961	2.779456
C	0.885446	-2.533938	1.138439
H	-0.114227	-2.177427	1.442115
C	1.691947	-3.260536	2.056593

3c→4c'			
-3799.8422569			
Ru	0.679814	-0.081821	0.531205
Cl	-0.462253	4.439804	2.855137
Cl	-1.426363	6.017520	-2.255679
N	0.439901	-2.611301	-1.208014
N	2.520552	-1.879879	-1.017577
N	0.284933	1.220776	-1.061163
O	0.354122	1.835413	1.587415
O	-3.858528	-0.104886	-0.123507
C	1.225800	-1.640470	-0.599904
C	1.239251	-3.612034	-1.952272
H	0.799493	-3.804753	-2.953089
H	1.241752	-4.579730	-1.400656
C	2.623509	-2.965341	-2.013573
H	3.445734	-3.661295	-1.748820
H	2.857379	-2.542538	-3.018094
C	-0.905090	-3.037465	-0.908654
C	-1.912603	-2.868496	-1.895576
C	-3.177729	-3.450048	-1.678081
H	-3.953925	-3.329446	-2.452785
C	-3.469369	-4.194377	-0.520107
C	-2.445003	-4.355941	0.433045
H	-2.640841	-4.953679	1.339266
C	-1.157813	-3.810866	0.260319
C	-1.668256	-2.066033	-3.152173
H	-1.696408	-0.975112	-2.938016
H	-2.445739	-2.273728	-3.913862
H	-0.679046	-2.275428	-3.607535
C	-4.834856	-4.804908	-0.297190
H	-5.433841	-4.826270	-1.229690
H	-5.412700	-4.227723	0.457481
H	-4.758520	-5.843970	0.085533
C	-0.087677	-4.071236	1.288029
H	0.849086	-4.448047	0.825971
H	-0.426414	-4.822526	2.027638
H	0.193904	-3.143148	1.833565
C	3.767245	-1.290807	-0.584662
C	4.354353	-0.230393	-1.319208
C	5.625300	0.234798	-0.928292
H	6.076665	1.069545	-1.490845
C	6.326438	-0.323786	0.155301
C	5.735108	-1.406279	0.834523
H	6.277506	-1.880042	1.669903
C	4.473413	-1.918083	0.478609
C	3.650815	0.443536	-2.472094
H	3.051732	1.303410	-2.103217
H	2.958986	-0.237456	-3.004913
H	4.380759	0.838615	-3.206778
C	7.662413	0.233463	0.591175
H	7.523822	1.030930	1.354257
H	8.216262	0.685911	-0.256579
H	8.304036	-0.547184	1.048385
C	3.903919	-3.102318	1.219686
H	3.017001	-2.790961	1.813498
H	4.654221	-3.531873	1.911974
H	3.576646	-3.912047	0.532554
C	-1.172850	-0.412187	0.494712
H	-1.647205	-0.517855	-0.502532
C	-2.160205	-0.280648	1.552483
C	-3.558276	-0.129923	1.202670
C	-4.526994	0.020385	2.218529
H	-5.590518	0.128723	1.966398
C	-4.141601	0.038826	3.569078
H	-4.916517	0.158596	4.343857
C	-2.788548	-0.082276	3.931620
H	-2.488984	-0.053484	4.990350
C	-1.819996	-0.228039	2.934833
H	-0.755494	-0.316801	3.197797
C	-5.183323	0.252985	-0.587613
H	-5.592141	1.031977	0.096289
C	-6.107880	-0.967702	-0.599947
H	-5.708882	-1.743497	-1.284354
H	-7.121277	-0.679799	-0.948476
H	-6.207257	-1.419634	0.406850
C	-4.993883	0.865765	-1.974532
H	-4.323000	1.746271	-1.928293
H	-5.970263	1.189887	-2.388708
H	-4.550181	0.123750	-2.669991
C	0.282807	0.921506	-2.368122
H	0.575579	-0.105183	-2.624469
C	-0.080523	1.851534	-3.371009
H	-0.049335	1.539735	-4.425531
C	-0.478271	3.132328	-3.010627
H	-0.775445	3.878673	-3.761925
C	-0.505604	3.490154	-1.632829
C	-0.103191	2.497113	-0.668992
C	-0.081667	2.769414	0.741824
C	-0.488046	4.054518	1.157459
C	-0.903921	5.040829	0.230222
H	-1.214995	6.027913	0.599522
C	-0.909041	4.767799	-1.133987
Cl	1.352080	-1.132759	2.567116
H	1.712612	1.902504	1.578549
Cl	3.054333	1.689771	1.066042

H	-1.37089700	3.09506400	3.76669700	H	-0.774331	3.567110	2.887287	H	1.322002	-3.461337	3.073513
C	-1.93648300	4.01172300	1.87926400	C	-2.433394	3.768322	1.499564	C	2.939651	-3.703766	1.645255
H	-2.57763500	4.79182400	2.31674800	H	-3.063834	4.386475	2.156183	H	3.603402	-4.268443	2.317450
C	-1.77320300	3.96619000	0.46731500	C	-2.916667	3.439959	0.203098	C	3.380904	-3.431538	0.319832
C	-0.91345100	2.93910800	-0.06608900	C	-2.070974	2.618645	-0.648708	C	2.484918	-2.679234	-0.535578
C	-0.59261100	2.89261000	-1.46502300	C	-2.496847	2.319118	-1.998222	C	2.858657	-2.374625	-1.897792
C	-1.24710200	3.78991500	-2.32205400	C	-3.747087	2.817211	-2.421072	C	4.105614	-2.838581	-2.355231
C	-2.14856100	4.76577800	-1.82254600	C	-4.582014	3.591349	-1.585116	C	4.986592	-3.571871	-1.524182
H	-2.63242700	5.45810600	-2.52543500	H	-5.547269	3.947923	-1.969895	H	5.950808	-3.906539	-1.931056
C	-2.39013700	4.86673900	-0.45798400	C	-4.173260	3.899642	-0.295421	C	4.634372	-3.861604	-0.212097
H	0.29992900	1.48373000	-2.68102200	H	-0.962144	1.202931	-2.495821	H	1.265437	-1.342794	-2.258058
Cl	0.69053500	-0.13879800	-3.43872200	Cl	0.750654	-0.011155	-2.901118	Cl	-0.700345	-0.167692	-2.834377

4c'→5b	2b	5b	4c'
**-3799.8326341**	**-4274.4098975**	**-2403.6708311**	**-3799.8496541**

4c'→5b (-3799.8326341)

Ru	-1.275090	-0.184288	0.751958
Cl	4.767770	-2.176085	2.887190
Cl	5.564529	-4.037299	-2.149089
N	-1.695231	1.222193	-1.956785
N	-3.273454	-0.237475	-1.390281
N	1.028257	-1.921529	-0.630969
O	2.043365	-1.621817	2.001134
O	2.729456	1.955888	0.577838
C	-2.116071	0.374537	-0.949856
C	-2.661610	1.265608	-3.080354
H	-2.139660	1.216162	-4.057911
H	-3.228617	2.224245	-3.050855
C	-3.551707	0.047946	-2.810270
H	-4.630514	0.246710	-2.972043
H	-3.274377	-0.828333	-3.441919
C	-0.794630	2.343503	-1.861393
C	0.388575	2.340445	-2.643359
C	1.176746	3.509070	-2.676830
H	2.089418	3.511041	-3.296485
C	0.825115	4.669743	-1.963514
C	-0.354867	4.637789	-1.193786
H	-0.658177	5.537900	-0.632942
C	-1.187280	3.504230	-1.136709
C	0.821430	1.119471	-3.420188
H	1.129585	0.300217	-2.737615
H	1.682557	1.352152	-4.076869
H	0.010700	0.710173	-4.058405
C	1.671459	5.921690	-2.023295
H	2.507180	5.819290	-2.743807
H	2.108205	6.161259	-1.030060
H	1.067205	6.802998	-2.326582
C	-2.464679	3.547590	-0.335978
H	-3.340208	3.213795	-0.931952
H	-2.671495	4.575784	0.019340
H	-2.421608	2.876239	0.548637
C	-4.171725	-1.096702	-0.653150
C	-3.928797	-2.489880	-0.542619
C	-4.871501	-3.282485	0.143617
H	-4.676795	-4.363784	0.239454
C	-6.045812	-2.742340	0.697417
C	-6.276772	-1.361951	0.533623
H	-7.196911	-0.915770	0.946938
C	-5.365371	-0.523076	-0.132216
C	-2.695225	-3.138840	-1.121083
H	-1.816282	-2.979253	-0.458778
H	-2.442252	-2.731770	-2.121139
H	-2.836082	-4.232667	-1.224841
C	-7.017737	-3.608473	1.465906
H	-6.774734	-3.603021	2.551391
H	-6.982396	-4.664327	1.129158
H	-8.060858	-3.245679	1.364001
C	-5.647175	0.955727	-0.246212
H	-4.902869	1.525833	0.349770
H	-6.657040	1.195646	0.139980
H	-5.591632	1.324329	-1.292515
C	0.147158	0.996668	0.787672
H	0.852748	0.784774	-0.050412
C	0.668084	1.922643	1.780972
C	2.026361	2.400144	1.653032
C	2.560472	3.269192	2.630100
H	3.592507	3.635658	2.546857
C	1.781146	3.671871	3.726240
H	2.222389	4.347117	4.477285
C	0.459227	3.214910	3.871886
H	-0.146424	3.522716	4.737959
C	-0.077391	2.348438	2.915087
H	-1.101561	1.962324	3.024933
C	4.141346	2.247828	0.430962
H	4.611010	2.210517	1.440481
C	4.348279	3.630021	-0.195492
H	3.890151	3.662905	-1.204945
H	5.431636	3.849043	-0.294147
H	3.889573	4.433407	0.414364
C	4.721830	1.121587	-0.421247
H	4.552017	0.138223	0.058961
H	5.813923	1.263640	-0.551551
H	4.250556	1.109080	-1.425557
C	0.570738	-2.122527	-1.862859
H	-0.445647	-1.751561	-2.070411
C	1.312065	-2.765004	-2.890544
H	0.860528	-2.917624	-3.882490
C	2.608071	-3.171391	-2.617827
H	3.234683	-3.656771	-3.381276
C	3.156252	-2.953375	-1.322422
C	2.297793	-2.338732	-0.324544
C	2.783877	-2.135580	1.023350
C	4.128266	-2.468746	1.289723
C	4.976367	-3.042551	0.313301
H	6.010209	-3.297697	0.584010
C	4.496528	-3.294353	-0.965236
Cl	-2.947118	0.796831	2.025356
H	1.053769	-1.706424	1.837041
Cl	-0.937711	-1.981835	2.283710

2b (-4274.4098975)

Ru	0.087051	-0.052250	-0.166373
Cl	-4.691726	1.937373	0.218974
Cl	-5.399635	-2.234913	3.646579
Cl	-0.058809	6.712063	1.659862
Cl	0.697689	4.087550	-3.054069
N	1.188995	-1.858565	-2.417656
N	-1.009226	-1.812273	-2.452576
N	-0.916805	-1.434015	1.064272
N	0.089389	1.505771	1.311686
O	-1.970427	0.668319	-0.234078
O	0.554016	1.603354	-1.326599
O	3.400336	-1.692820	2.461494
C	0.085463	-1.334654	-1.777573
C	0.848994	-2.784151	-3.519863
H	1.131138	-3.826441	-3.249230
H	1.409115	-2.515530	-4.439574
C	-0.664956	-2.599306	-3.657503
H	-0.951350	-2.032031	-4.571577
H	-1.224247	-3.556986	-3.671520
C	2.587164	-1.621767	-2.187521
C	3.356028	-2.584642	-1.484270
C	4.741782	-2.365681	-1.343953
H	5.340168	-3.100870	-0.779701
C	5.379950	-1.243765	-1.902369
C	4.595517	-0.338975	-2.647824
H	5.081316	0.528860	-3.124974
C	3.209899	-0.509034	-2.819633
C	2.733539	-3.833555	-0.899985
H	3.193350	-4.084461	0.077134
H	2.891466	-4.711894	-1.564792
H	1.642103	-3.721868	-0.748920
C	6.858857	-1.000209	-1.703969
H	7.385918	-1.915842	-1.367958
H	7.028300	-0.215978	-0.934017
H	7.343784	-0.646085	-2.637353
C	2.417173	0.455756	-3.666424
H	1.808165	-0.075841	-4.428466
H	3.089667	1.156259	-4.198968
H	1.711781	1.052863	-3.049852
C	-2.405331	-1.496019	-2.292873
C	-3.258836	-2.453445	-1.691823
C	-4.642573	-2.183460	-1.651237
H	-5.311302	-2.916088	-1.169103
C	-5.187537	-1.009491	-2.202509
C	-4.309372	-0.095004	-2.819481
H	-4.716250	0.831000	-3.258408
C	-2.921686	-0.312339	-2.882808
C	-2.722440	-3.749889	-1.127164
H	-3.478914	-4.244617	-0.487132
H	-1.807913	-3.592534	-0.520927
H	-2.457212	-4.472001	-1.932080
C	-6.665594	-0.706813	-2.109450
H	-6.843393	0.125346	-1.393861
H	-7.246364	-1.583993	-1.760771
H	-7.079753	-0.384638	-3.088055
C	-2.014513	0.698062	-3.540574
H	-1.336129	1.170650	-2.799630
H	-2.603544	1.500081	-4.025872
H	-1.368355	0.237059	-4.318308
C	1.795782	-0.644187	0.431508
H	1.928943	-1.731127	0.622838
C	2.940080	0.095960	0.958683
C	3.785254	-0.474155	1.977899
C	4.908090	0.238123	2.449819
H	5.551549	-0.181516	3.235609
C	5.214543	1.505594	1.922052
H	6.093816	2.046954	2.307318
C	4.414597	2.076129	0.917257
H	4.664126	3.062266	0.496598
C	3.292807	1.376097	0.455323
H	2.670158	1.787352	-0.350224
C	4.163078	-2.362465	3.491633
H	4.489836	-1.602753	4.238567
C	5.387334	-3.065303	2.896150
H	5.070083	-3.853562	2.182255
H	5.986583	-3.545622	3.697081
H	6.043884	-2.355706	2.354628
C	3.197150	-3.330253	4.173272
H	2.322854	-2.788795	4.585733
H	3.705223	-3.861585	5.003485
H	2.829331	-4.088569	3.451153
C	-0.404435	-2.530667	1.653457
H	0.641932	-2.770949	1.419734
C	-1.145194	-3.329701	2.556458
H	-0.663744	-4.212927	3.002448
C	-2.452390	-2.987562	2.874015
H	-3.050541	-3.580132	3.582106
C	-3.037781	-1.836869	2.269407
C	-2.228727	-1.084719	1.337743
C	-2.727124	0.080010	0.630864
C	-4.048602	0.493658	0.972185
C	-4.843622	-0.216943	1.890242
H	-5.859244	0.140771	2.111677
C	-4.359165	-1.368252	2.518226
C	-0.092038	1.404113	2.632087
H	-0.137667	0.384268	3.044386
C	-0.221164	2.545286	3.464130

5b (-2403.6708311)

Ru	0.625211	-0.753351	-0.448582
N	0.428630	2.238676	-0.091365
N	2.473862	1.412807	0.124021
O	-3.505642	-1.368586	1.036066
C	1.148691	1.063479	-0.067826
C	1.267036	3.422187	0.188293
H	1.059101	3.814127	1.211027
H	1.044671	4.235273	-0.532858
C	2.691448	2.868043	0.048571
H	3.153531	3.143743	-0.926723
H	3.373284	3.205664	0.856082
C	-0.992888	2.433041	-0.145029
C	-1.739167	2.412529	1.060586
C	-3.116887	2.705714	0.996861
H	-3.703693	2.695529	1.930961
C	-3.759514	3.011928	-0.218495
C	-2.985168	3.017290	-1.396903
H	-3.469495	3.250837	-2.360229
C	-1.604367	2.740120	-1.388374
C	-1.087250	2.042266	2.373385
H	-1.819963	2.071343	3.203202
H	-0.255812	2.729930	2.638473
H	-0.654279	1.019693	2.331221
C	-5.239806	3.319888	-0.270003
H	-5.683069	3.377096	0.744174
H	-5.793503	2.541111	-0.837825
H	-5.435361	4.286347	-0.781214
C	-0.801746	2.761556	-2.667123
H	0.007320	3.523189	-2.631531
H	-1.446786	3.000642	-3.535115
H	-0.309949	1.782753	-2.852795
C	3.592064	0.530616	0.338705
C	3.892161	0.135306	1.671650
C	5.003932	-0.698938	1.886659
H	5.232600	-1.018755	2.917239
C	5.829234	-1.132783	0.829523
C	5.540752	-0.669626	-0.467607
H	6.196092	-0.965227	-1.304313
C	4.445142	0.175977	-0.740171
C	3.049094	0.599511	2.835204
H	3.549707	0.377944	3.798209
H	2.068034	0.078718	2.830921
H	2.848717	1.690872	2.793748
C	6.982436	-2.077251	1.083644
H	7.753367	-2.008308	0.289856
H	6.629887	-3.132015	1.109357
H	7.470175	-1.875958	2.059609
C	4.247539	0.724579	-2.133851
H	3.182220	0.715092	-2.439407
H	4.816841	0.129378	-2.874810
H	4.618944	1.771667	-2.205334
C	-1.216058	-0.702049	-0.336234
H	-1.761602	0.109832	0.179611
C	-2.016699	-1.855740	-0.744891
C	-3.222452	-2.173878	-0.023909
C	-4.002019	-3.283867	-0.412170
H	-4.926354	-3.536156	0.125759
C	-3.599560	-4.090556	-1.491497
H	-4.222992	-4.953709	-1.775455
C	-2.423685	-3.801620	-2.203834
H	-2.117782	-4.426973	-3.056391
C	-1.647066	-2.697693	-1.828741
H	-0.744267	-2.435109	-2.403368
C	-4.679095	-1.589425	1.852584
H	-4.819286	-2.687541	1.979501
C	-5.917280	-0.977258	1.190662
H	-5.785780	0.118311	1.075545
H	-6.819197	-1.158186	1.811335
H	-6.100200	-1.408868	0.186218
C	-4.364138	-0.977673	3.216220
H	-3.459494	-1.443199	3.655005
H	-5.213476	-1.130067	3.912813
H	-4.182145	0.112394	3.118908
Cl	1.111336	-1.928172	1.502141
Cl	1.023386	-0.570634	-2.751332

4c' (-3799.8496541)

Ru	0.719838	-0.004917	0.575342
Cl	-1.339837	4.220487	2.993596
Cl	-2.327299	5.753488	-2.116248
N	0.854506	-2.476073	-1.216977
N	2.828890	-1.504622	-0.967445
N	0.134179	1.287398	-0.957685
O	-0.026010	1.844017	1.746024
O	-3.755133	-0.383629	-0.221582
C	1.505366	-1.421905	-0.583847
C	1.779651	-3.361123	-1.958987
H	1.389292	-3.576539	-2.975778
H	1.877772	-4.336040	-1.429970
C	3.082325	-2.561732	-1.967122
H	3.968926	-3.163975	-1.681303
H	3.300940	-2.102080	-2.958560
C	-0.437927	-3.063466	-0.963880
C	-1.440339	-2.973763	-1.966006
C	-2.637919	-3.696813	-1.792297
H	-3.411318	-3.634805	-2.576733
C	-2.866196	-4.504551	-0.663311
C	-1.841117	-4.595359	0.299086
H	-1.983961	-5.243741	1.179954
C	-0.617477	-3.911485	0.166936
C	-1.265630	-2.112203	-3.194754
H	-1.463149	-1.042936	-2.960851
H	-1.973362	-2.411444	-3.993194
H	-0.238657	-2.162626	-3.609668
C	-4.162957	-5.261338	-0.482735
H	-4.776237	-5.253790	-1.405942
H	-4.775462	-4.815570	0.331058
H	-3.980355	-6.319624	-0.201115
C	0.466601	-4.103979	1.195229
H	1.427241	-4.412930	0.730330
H	0.180359	-4.885432	1.925662
H	0.682143	-3.164428	1.751144
C	3.997242	-0.759572	-0.553192
C	4.436218	0.357452	-1.306322
C	5.647151	0.977794	-0.943236
H	5.980847	1.858561	-1.517437
C	6.431876	0.517550	0.128906
C	5.990782	-0.624000	0.825170
H	6.602390	-1.022061	1.652432
C	4.793601	-1.288351	0.498306
C	3.627479	0.932219	-2.442462
H	2.868191	1.636486	-2.041149
H	3.089120	0.154966	-3.021327
H	4.272480	1.496890	-3.144916
C	7.697758	1.236260	0.537802
H	7.490551	1.960224	1.356548
H	8.135537	1.810262	-0.303974
H	8.467490	0.531727	0.914708
C	4.388445	-2.528258	1.257453
H	3.467050	-2.337984	1.849826
H	5.192151	-2.842971	1.951559
H	4.174837	-3.384045	0.580940
C	-1.078125	-0.621660	0.471073
H	-1.523594	-0.679317	-0.543886
C	-2.115970	-0.702454	1.489057
C	-3.507112	-0.602307	1.099025
C	-4.522621	-0.688568	2.075956
H	-5.581643	-0.618124	1.792764
C	-4.189793	-0.856010	3.430601
H	-4.999962	-0.916070	4.175721
C	-2.845582	-0.934584	3.835910
H	-2.589204	-1.054005	4.899757
C	-1.832676	-0.846796	2.876433
H	-0.772906	-0.895538	3.170878
C	-5.096418	-0.131436	-0.704192
H	-5.631105	0.484521	0.055055
C	-5.850319	-1.444234	-0.935813
H	-5.328080	-2.054970	-1.700021
H	-6.880408	-1.239696	-1.294156
H	-5.923171	-2.047487	-0.009262
C	-4.939132	0.693013	-1.980997
H	-4.387595	1.632278	-1.778598
H	-5.934385	0.952812	-2.395558
H	-4.381820	0.119288	-2.750008
C	0.230170	1.043382	-2.273645
H	0.700252	0.089968	-2.547782
C	-0.239730	1.933996	-3.265815
H	-0.111879	1.668089	-4.325520
C	-0.861259	3.117004	-2.889561
H	-1.249611	3.829676	-3.631250
C	-1.004006	3.411176	-1.504206
C	-0.474765	2.470393	-0.547098
C	-0.573339	2.722563	0.858376
C	-1.218359	3.890201	1.290600
C	-1.764108	4.817785	0.367508
H	-2.264327	5.721773	0.740675
C	-1.650476	4.583775	-0.996770
Cl	1.491143	-1.048768	2.584194
H	1.032589	2.037536	1.768882
Cl	2.615252	1.802311	0.993231

	H -0.373554 2.408950 4.545110<br>C -0.163030 3.814472 2.902728<br>H -0.268767 4.720548 3.518041<br>C 0.033724 3.955997 1.497688<br>C 0.163693 2.746606 0.728336<br>C 0.380828 2.743976 -0.697746<br>C 0.437436 4.012836 -1.324308<br>C 0.299039 5.213617 -0.592537<br>H 0.348415 6.173402 -1.125784<br>C 0.104861 5.192126 0.789248		

[i] Instead of a normal ^{13}C measurment an APT measurment was performed

[ii] Gaussian 09, Revision A.1, M.J. Frisch, G.W. Trucks, H.B. Schlegel, G.E. Scuseria, M.A. Robb, J.R. Cheeseman, G. Scalmani, V. Barone, B. Mennucci, G.A. Petersson, H. Nakatsuji, M. Caricato, X. Li, H.P. Hratchian, A.F. Izmaylov, J. Bloino, G. Zheng, J.L. Sonnenberg, M. Hada, M. Ehara, K. Toyota, R. Fukuda, J. Hasegawa, M. Ishida, T. Nakajima, Y. Honda, O. Kitao, H. Nakai, T. Vreven, J.A. Montgomery, Jr., J.E. Peralta, F. Ogliaro, M. Bearpark, J.J. Heyd, E. Brothers, K.N. Kudin, V.N. Staroverov, R. Kobayashi, J. Normand, K. Raghavachari, A. Rendell, J.C. Burant, S.S. Iyengar, J. Tomasi, M. Cossi, N. Rega, J.M. Millam, M. Klene, J.E. Knox, J.B. Cross, V. Bakken, C. Adamo, J. Jaramillo, R. Gomperts, R.E. Stratmann, O. Yazyev, A.J. Austin, R. Cammi, C. Pomelli, J.W. Ochterski, R.L. Martin, K. Morokuma, V.G. Zakrzewski, G.A. Voth, P. Salvador, J.J. Dannenberg, S. Dapprich, A.D. Daniels, Ö. Farkas, J.B. Foresman, J.V. Ortiz, J. Cioslowski, and D.J. Fox, Gaussian, Inc., Wallingford CT, 2009.

[iii] (*a*) A. Becke, *Phys. Rev. ,A* 1988, **38**, 3098-3100; (*b*) J.P. Perdew, *Phys. Rev. B*, 1986, **33**, 8822-8824; (*c*) J.P. Perdew, *Phys. Rev. B*, 1986, **34**, 7406-7406.

[iv] (*a*) U. Haeusermann, M. Dolg, H. Stoll and H. Preuss, *Mol. Phys.*, 1993, ***78***, 1211-1224; (*b*) W. Kuechle, M. Dolg, H. Stoll and H. Preuss, *J. Chem. Phys*,. 1994, **100**, 7535-7542; (*c*) T. Leininger, A. Nicklass, H. Stoll, M. Dolg and P. Schwerdtfeger, *J. Chem. Phys.*, 1996, **105**, 1052-1059.

[v] A. Schaefer, H. Horn and R. Ahlrichs, *J. Chem. Phys*,. 1992, **97**, 2571-2577.

[vi] D. Rappoport and F. Furche, *J. Chem. Phys.*, 2010, **133**, 134105.

[vii] Y. Zhao and D.G. Truhlar, *Theor. Chem. Acc.*, 2008, **120**, 215-241.

[viii] (*a*) V. Barone and M. Cossi, *J. Phys. Chem. A*, 1998, **102**, 1995-2001; (*b*) J. Tomasi and M. Persico, *Chem. Rev.*, 1994, **94**, 2027-2094.
